# Tailoring of energetic groups in acroyloyl polymers

**DOI:** 10.1080/15685551.2016.1258977

**Published:** 2016-11-24

**Authors:** Deepak Kumar, K. Durga Bhaskar Yamajala, Asit B. Samui, Shaibal Banerjee

**Affiliations:** ^a^ Organic Synthesis Laboratory, Department of Applied Chemistry, Defence Institute of Advanced Technology (DU), Girinagar, India

**Keywords:** Acrylamide polymers, click chemistry, triazole polymers

## Abstract

Acryloyl based novel energetic monomers having nitro acrylates and nitro triazole acrylates were synthesized and further used for polymerization. Due to scavanging properties of nitro groups, syntheses of nitro aromatic polymers are not facile at normal conditions. In this regard, we report a simple protocol to synthesize these energetic group embeded acroloyl polymers. These polymers were characterized by FTIR, and NMR spectroscopic techniques. gel permeation chromatography (GPC) technique was employed in order to understand molecular mass of these polymers along with average molecular weight, number average weight and poly dispersity index. Glass transition temperature (*T*_g_) was determined by using DSC analysis. It was observed that with increase in nitro groups in polymers there is a decrease in glass transition temperature. Two steps degradation were depicted in the TGA thermograph in nitro containing polymers. Heat release during this reaction was found up to 951 J/g. Increase in nitrogen content in polymer unit enhanced the heat release of polymers.

## 1. Introduction

Acrylate polymers have ubiquitous applications in areas such as leather,[1] textile,[2] pharmacological drugs,[3] fiber optics,[4] metal complexes, polymer supports and building materials.[5] Studies of homopolymers, copolymers and blends acrylate have been reported in literature.[6] Introduction of phenyl ring into acrylate polymers, enhances the thermal stability which is useful in industrial applications such as peptide synthesis. Copolymer of Methyl methacrylates and phenyl acrylates exhibit photoluminscent properties.[7] Acrylamides are another class of acryloyl based polymers which are readily synthesized using primary amines.[8] Amine is an important functional group having multiple applications. Controlled radical polymerization of primary amines containing homopolymers and copolymers have been tolerant to many functional groups and variety of polar monomers.[9–11] Various types of controlled radical polymerization methods such as atom transfer radical polymerization (ATRP),[12] initiator-transfer-terminator polymerization,[13–15] nitroxide-mediated polymerization,[16,17] and reversible addition–fragmentation transfer polymerization (RAFT) [18] are reported in the literature. These acrylamide based polymers are useful in thermite application to bind the surface of nanoparticles.[19] However, energetic groups tailored aromatic acrylamide are not reported in the literature.

In addition to this another class of GAP based energetic polymers containing triazole and tetrazole ring are well known energetic materials.[20] Due to their sparing solubility in organic solvents, polymers containing tetrazoles as compared to triazoles are endeavored to a lesser extend in energetic applications. 1,2,3-triazole and 1,2,4-triazole compounds have directed recent attention as an energetic materials due to their high energy and nitrogen content. The heat of formation of 1,2,4-triazole and 1,2,3-triazole are 109 kJ/mol and 272 kJ/mol, respectively.[21]

Copper catalyzed 13-cycloaddition reaction is a powerful tool for the formation of triazole based polymers which involves the mechanism of Hüisgen cycloaddition.[22,23] Presently, few monomers have been synthesized in well-organized manner from organic azides and propargyl acrylate via copper catalyzed dipolar cyclo additions and further, polymerized using AIBN as a free radical initiator.

Energetic polymers are known to be useful in the field of nanoenergetic composites. Nanoenergetic materials are of great interest in combustion studies such as igniters and primers which are very useful for defence applications.[24] However, these materials are sensitive to impact, friction and electrostatic discharge (ESD). Among various performance parameters, ESD is the most important tool for safe handling of energetic materials. To reduce ESD sensitivity, the nanoenergetic composite is embedded in polymer matrix. Polymer matrix also reduces the surface charge on the oxidizer surface which will contribute in decreasing the ESD ignition sensitivity of the energetic composites.[24]

Polymer matrix not only lowers the sensitivity of nanoenergetic materials but also reduces the burn rate which is useful for controlled combustion. Incorporation of energetic groups like azido, nitro, N-nitro and tri fluoro in the polymer enhances the energetic properties of the nanocomposites.

Herein we describe the short-step synthesis of energetic groups tailored acrylamide/acrylate based polymers. These polymers are cost effective, low vulnerable with high amount of energy. The energetic groups introduced are nitro, trifloromethyl benzene & triazoles (TrZ) in the polymer backbone. Radical polymerization techniques were applied in this regard. In–CF_3_ containing polymer, when –H present on the backbone of the polymer is replaced by fluorine containing moiety increases its density which in turn raises the oxygen balance and enhances the performance parameter of energetic molecules.[25] However, there are few reports on synthesis of energetic groups having acrylamide or acrylate based polymers. These polymers were characterized by spectroscopic techniques and detailed thermal characterization was also performed. Our aim is to incorporate energetic groups containing aromatic ring in the acrylamide backbone to enhance the heat content of the polymers. Nitro and triazole containing polymers synthesized by us can be further explored in the area of nanoenergetic composites. The monomer (1a–1e) unit was prepared by condensation of aniline derivatives with acryol chloride.

## 2. Experimental

### 2.1. Materials and methods

4-Nitroaniline (Sigma Aldrich), 3-Nitroaniline(Alfa Aesar), 3-(trifluoromethyl) aniline, 4-Nitro-2-(trifluoromethyl) aniline(Alfa Aesar), 3,5-dinitro aniline (Sigma Aldrich), 2,4-dinitro aniline (Sigma Aldrich), Sodium Azide (Sigma Aldrich), Copper Sulphate and Sodium ascorbate were used as received. Dichloromethane (Merck) was distilled. Triethyl amine was also distilled. Acryloyl chloride(Alfa Aesar) were used under inert conditions. AIBN (Sigma Aldrich) as obtained. All the solvents were purified by distillation prior to their use.

### 2.2. Instruments

^1^H-NMR and ^13^C-NMR spectra were recorded on a Bruker-500 and 125 MHz instrument, respectively using tetramethylsilane (TMS) as an internal reference and DMSO-*d*6 as solvent. Coupling constants (J values) are given in Hertz (Hz). Chemical shifts are expressed in parts per million (ppm) downfield from internal reference, tetramethylsilane. The standard abbreviations s, d, t, q, quin, m and dd refer to singlet, doublet, triplet, quartet, quintet, multiplet and doublet of doublet respectively. IR spectra were recorded on a BRUKER ATR FT-IR spectrometer as a neat sample in the range of 500 to 4000 cm^−^^1^. High resolution mass 35 spectra measurements were carried out on Micromass Q-Tof Mass spectrometer. Melting points were recorded on BUCHI M560. Differential Scanning Calorimetry (DSC) studies were carried out on a Perkin Elmer DSC-7 instrument operating at a heating rate of 10 °C/min in nitrogen atmosphere with 1 to 2 mg of sample. Thermal decomposition was also undertaken on a Perkin Elmer operating at a heating rate range from 10 °C/min.

### 2.3. General procedure for synthesis of *N*-(phenyl) acrylamide (1a–1e)

Substituted nitro aniline (36 mmol, 1 eq.) was dissolved in 100 ml of DCM in a two necked R.B. flask. Then the reaction mixture was kept for stirring 10–15 min under N_2_ atmosphere. Triethylamine (108 mmol, 3 eq.) was added dropwise at 0°–5 °C. Acroyl chloride (54.33 mmol, 1.5 eq.) was dissolved in 50 ml of dry DCM and was added dropwise with constant stirring. The reaction was continued for 1 h in cold condition and room temperature for 5 h. TLC was checked and the consumption of starting material was observed. Then the reaction mixture was diluted with the ethyl acetate (3 × 25 ml) and then washed with distilled water. Organic layer washed with brine solution and dried with anhydrous Na_2_SO_4_. The organic layer was concentrated under reduced pressure. The crude product was purified by column chromatography by using ethyl acetate/ hexanes mixture as eluent.

#### 2.3.1. *N*-(3-nitrophenyl) acrylamide (1a)

5.31 g, 75% yield, (20% EA+ n-Hexanes),m.p. 145 °C ^1^H NMR (500 MHz,DMSO-*d*_6_) *δ* = 10.64 (s, 1 H), 8.71 (t, *J* = 2.1 Hz, 1 H), 8.05–7.91 (m, 2 H), 7.64 (t, *J* = 8.2 Hz, 1 H), 6.54–6.25 (m, 2 H), 5.85 (dd, *J* = 10.11.8 Hz, 1 H). ^13^C NMR (125 MHz, DMSO-*d*_6_) *δ* = 164.2, 148.4, 140.6, 131.7, 130.7, 125.8, 118.6, 114.0. HRMS (Q-Tof) for C_9_H_8_N_2_O_3_ [M+H]^+^ calc. 192.0532 found 192.0535.

#### 2.3.2. *N*-(4-nitrophenyl) acrylamide (1b)

4.58 g, 65% yield, (20% EA+ n-Hexanes), m.p. 172 °C ^1^H NMR (500 MHz, DMSO-*d*_6_) *δ* = 10.74 (br. s., 1 H), 8.24 (d, *J* = 8.5 Hz, 2 H), 7.91 (d, *J* = 9.2 Hz, 2 H), 6.56–6.25 (m, 2 H), 5.87 (d, *J* = 9.8 Hz, 1 H). ^13^C NMR (125 MHz, DMSO-*d*_6_) *δ* = 164.0, 145.7, 142.8, 131.7, 129.0, 125.5, 120.0. HRMS (Q-Tof) for C_9_H_8_N_2_O_3_ [M+H]^+^ calc. 192.0532 found 192.0535.

#### 2.3.3. *N*-(3-(trifluoromethyl) phenyl) acrylamide) (1c)

5.21 g, 78% yield, (23% EA+ n-Hexanes)m.p. 87 °C, ^1^H NMR (500 MHz, DMSO-*d*_6_) *δ* = 10.46 (s, 1 H), 8.19 (s, 1 H), 7.87 (d, *J* = 8.9 Hz, 1 H), 7.54 (t, *J* = 7.9 Hz, 1 H), 7.42–7.34 (d, *J* = 10 Hz, 1 H), 6.52–6.40 (m, 1 H), 6.37–6.24 (m, 1 H), 5.79 (dd, *J* = 2.1, 10.1 Hz, 1 H). ^13^C NMR (125 MHz, DMSO-*d*_6_) *δ* = 164.0, 140.3, 131.9, 130.4, 130.3, 130.16, 130.03 (q, *J* = 31.25 Hz), 129.66, 127.9, 125.6, 122.4(*J*_C-F_ = 271.25 Hz), 121.27, 120.1, 120.7115.9. ^19^F NMR (CDCl_3_, 376 MHz) *δ* −62.7 (3F,s, CF_3_). HRMS (Q-Tof) for C_10_H_8_F_3_NO [M+H]^+^ calc. 215.0558 found 215.0559.

#### 2.3.4. *N*-(4-nitrophenyl)-3-(trifluoromethyl) phenyl) acrylamide) (1d)

5.16 g, 80% yield, (40% EA+ n-Hexanes)m.p. 153 °C, ^1^H NMR (500 MHz,DMSO-*d*_6_) *δ* = 10.88 (br. s., 1 H), 8.28 (s, 1 H), 8.19–8.01 (m, 2 H), 6.48–6.28 (m, 2 H), 5.86 (d, *J* = 9.8 Hz, 1 H). ^13^C NMR (125 MHz, DMSO-*d*_6_) *δ* = 164.5, 144.1, 141.8, 131.3, 129.3, 128.01, 125.7, 123.9, 123.46 (q, *J* = 33.7 Hz,) 123.53, 123.46 (q, *J* = 33.7 Hz), 123.07, 122.73, 122.31(q, *J*_C-F_ = 253.3 Hz), 121.4, 119.2, 117.8. ^19^F NMR (CDCl_3_, 376 MHz) δ −60.0 (3F, s, CF_3_). HRMS (Q-Tof) for C_10_H_7_F_3_N_2_O_3_ [M+H]^+^ calc. 260.0432 found 260.0409.

#### 2.3.5. *N*-(35-dinitro phenyl acrylamide) (1e)

1.71 g, 65% yield, (52% EA+ n-Hexane)m.p. 179 °C, ^1^H NMR (500 MHz,DMSO-*d*_6_) *δ* = 11.04 (s, 1 H), 8.92 (d, *J* = 2.1 Hz, 2 H), 8.52 (t, *J* = 2.1 Hz, 1 H), 6.53–6.24 (m, 2 H), 6.05–5.75 (m, 1 H). ^13^C NMR (125 MHz, DMSO-*d*_6_) *δ* = 164.7, 148.7, 141.5, 131.2, 129.7, 119.1, 113.0. HRMS (Q-Tof) for C_9_H_7_N_3_O_5_ [M+H]^+^ calc. 237.0398 found 237.0386.

### 2.4. General procedure for synthesis of 1(1-(phenyl)-1*H*-1,2,3- triazol-4-yl) acrylate (2a–2c)

1-azido-3-nitrobenzene (5.44 mmol, 1 eq.) was taken in 30% *t*-butanol-water mixture. In this reaction mixture of prop-2-yn-1-yl acrylate (6.53 mmol, 1.2 eq.) and copper sulphate (1.08 mmol, 0.2 eq.), and sodium ascorbate (0.54 mmol, 0.1 eq.) were added. Then the reaction mixture was stirring at room temperature for overnight. TLC was analyzed and it showed consumption of starting material. The reaction mixture was diluted with ethyl acetate (3 × 25 ml) and washed with distilled water, brine solution and dried using anhydrous Na_2_SO_4_. The organic layer was concentrate under reduced pressure. The crude products were purified by column chromatography by using ethyl acetate/hexanes mixture as the eluent.

#### 2.4.1. (1-(3-nitrophenyl)-1*H*-1,2,3-triazol-4-yl) acrylate (2a)

1.72 g, 68% yield, (57% EA+ n-Hexanes) m.p. 88 °C, ^1^H NMR (500 MHz, CHLOROFORM-d) *δ* = 8.63 (t, *J* = 2.1 Hz, 1 H), 8.33 (ddd, *J* = 0.9, 2.1, 8.2 Hz, 1 H), 8.24 (s, 1 H), 8.20 (ddd, *J* = 0.9, 2.1, 8.2 Hz, 1 H), 7.78 (t, *J* = 8.2 Hz, 1 H), 6.49 (dd, *J* = 1.4, 17.2 Hz, 1 H), 6.18 (dd, *J* = 10.4, 17.4 Hz, 1 H), 5.91 (dd, *J* = 1.410.5 Hz, 1H), 5.43(s, 2H).^13^CNMR(125 MHz,CDCl_3_) 165.9, 148.9, 144.3, 137.6, 131.9, 131.1, 127.8, 126.0, 123.4, 122.2, 115.4, 57.4 .HRMS (Q-Tof) for C_12_H_10_N_4_O_4_ [M+H]^+^ calc. 274.0702 found 274.0692.

#### 2.4.2. (1-(4-nitrophenyl)-1*H*-1,2,3-triazol-4-yl) acrylate (2b)

1.77 g, 71% yield, (55% EA+ n-Hexanes) ^1^H NMR (500 MHz,CHLOROFORM-d) *δ* = 8.48–8.42 (m, 2 H), 8.22 (s, 1 H), 8.04–7.96 (m, 2 H), 6.47 (d, *J* = 5 Hz, 1 H), 6.23–6.13 (m, 1 H), 5.92(d, *J* = 10 Hz, 1 H), 5.43 (s, 2 H).

^13^C NMR (125 MHz CDCl_3_) *δ* = 166.0, 147.4, 144.4, 141.0, 131.9, 127.8, 125.6, 122.1, 120.6, 57.5. HRMS (Q-Tof) for C_12_H_10_N_4_O_4_ [M+H]^+^ calc. 274.0702 found 274.0688.

#### 2.4.3. (1-(3-trifloromethyl phenyl)-1*H*-1,2,3-triazol-4-yl) acrylate (2c)

1.35 g, 66% yield, (51% EA+ n-Hexanes) m.p. 110 °C, ^1^H NMR (500 MHz,CHLOROFORM-d) *δ* = 8.18 (s, 1 H), 8.03 (s, 1 H), 7.97 (d, *J* = 5.0 Hz, 1 H), 7.76–7.65 (m, 2 H), 6.46 (dd, *J* = 1.4, 17.2 Hz, 1 H), 6.16 (dd, *J* = 10.4, 17.4 Hz, 1 H), 5.89 (dd, *J* = 1.4, 10.5 Hz, 1 H), 5.41 (s, 2 H).

^13^ C NMR (125 MHz CDCl_3_) *δ* = 165.9, 143.9, 137.2, 132.4 (q, J = 33.7 Hz,) 131.8, 130.8, 127.8 (q, *J*_*C*-*F*_ = 281.25 Hz), 125.7, 124.4, 123.6, 122.2, 117.5, 57.6. ^19^F NMR (CDCl_3_, 376 MHz) *δ* −62.81 (3F, s, CF_3_). HRMS (Q-Tof) for C_13_H_10_F_3_N_3_O_2_ [M+H]^+^ calc. 297.0725 found 297.0722.

### 2.5. General procedure for polymerization (3a–3h)

(5.25 mmol, 1 eq.) of N-(phenyl) acrylamide and (0.105 mmol, 0.2 eq.) of AIBN were dissolved in 1,4-dioxane, flushed with nitrogen gas for 20 min and then reaction mixture was heated to 100 °C and reaction continued for 24 h under inert atmosphere. The color of the solution changed from grey to brown. During monitoring of reaction mixture by TLC, it was observed that starting material was consumed and a new spot was noticed at the bottom of the TLC. Methanol was slowly added to the reaction mixture to precipitate the polymer. The precipitate was further washed 2–3 times with methanol and then dried in oven at 50 °C.

#### 2.5.1. Poly (*N*-(3-nitrophenyl) acrylamide) (3a)

0.310 g, 62% yield, ^1^H NMR (500 MHz, DMSO-*d*_6_) *δ* = 10.63–9.96 (m, 1 H), 8.39 (br. s., 1 H), 7.97–7.50 (m, 2 H), 7.46–7.10 (m, 1 H), 2.58–2.22 (m, 1 H), 2.05–1.49 (m, 2 H), 1.36–1.06 (m, 1 H). ^13^C NMR (125 MHz, DMSO-*d*_6_) *δ* = 173.7, 147.9, 140.6, 129.9, 125.8, 117.7, 113.9, 35.9, 26.9.

#### 2.5.2. Poly (*N*-(4-nitrophenyl) acrylamide) (3b)

0.321 g, 64% yield, ^1^H NMR (500 MHz, DMSO-*d*_6_) *δ* = 10.77–10.10 (m, 1 H), 8.27–7.32 (m, 3 H), 2.51 (d, *J* = 1.7 Hz, 1 H), 2.12–1.53 (m, 1 H), 1.38–0.76 (m, 1 H). ^13^C NMR (125 MHz, DMSO-*d*_6_) *δ* = 174.1, 145.6, 142.4, 124.9, 119.6, 35.6, 26.9.

#### 2.5.3. Poly (*N*-(3-(trifluoromethyl) phenyl) acrylamide) (3c)

0.354 g, 75% yield, ^1^H NMR (400 MHz, DMSO-*d*_6_) *δ* = 10.16–9.71 (m, 1 H), 8.14–7.43 (m, 2 H), 7.36–7.02 (m, 1 H), 2.61–2.17 (m, 1 H), 2.06–1.55 (m, 1 H), 1.45–1.13 (m, 1 H). ^13^CNMR (125 MHz, DMSO-*d*_6_) *δ* = 173.6, 140.2, 129.5, 125.8, 123.5, 123.09, 120.4, 119.6, 116.3, 35.4, 26.9.

#### 2.5.4. Poly(*N*-(4-nitrophenyl)-3-(trifluoromethyl) phenyl) acrylamide) (3d)

0.278 g, 58% yield, ^1^H NMR (500 MHz, DMSO-*d*_6_) *δ* = 10.84–10.33 (m, 1 H), 8.35–7.49 (m, 2 H), 2.61–2.20 (m, 1 H), 2.04–1.35 (m, 2 H), 1.29–0.90 (m, 1 H). ^13^C NMR (125 MHz, DMSO-*d*_6_) *δ* = 178.8, 148.8, 146.2, 132.3, 128.1, 127.4, 125.9, 123.8, 122.5, 41.1, 29.1.

#### 2.5.5. Poly (*N*-(35-dinitro phenyl acrylamide) (3e)

0.256 g, 51% yield, ^1^H NMR (500 MHz, DMSO-*d*_6_) *δ* = 11.17–10.65 (m, 1 H), 9.18–8.08 (m, 2 H), 2.23–1.90 (m, 1 H), 1.87–0.90(m, 3H). ^13^C NMR (125 MHz, DMSO-*d*6) *δ* = 172.7, 140.4, 132.1, 130.5, 123.5, 119.91, 116.06, 29.51, 21.6.

#### 2.5.6. Poly (1-(3-nitrophenyl)-1*H*-123-triazol-4-yl) acrylate (3f)

0.362 g, 62% yield, ^1^H NMR (500 MHz, DMSO-*d*_6_) *δ* = 9.14–8.62 (m, 1 H), 8.57–7.50 (m, 3 H), 5.45–4.79 (m, 2 H), 2.43–2.06 (m, 1 H), 1.92–1.42 (m, 2 H), 1.34–0.97 (m, 1 H). ^13^C NMR (125 MHz, DMSO-*d*_6_) *δ* = 174.1, 148.6, 143.6, 137.2, 131.8, 126.1, 123.4, 114.9, 57.635.025.8.

#### 2.5.7. Poly (1-(4-nitrophenyl)-1*H*-123-triazol-4-yl) acrylate (3g)

0.367 g, 63% yield^1^H NMR (500 MHz, DMSO-*d*_6_) *δ* = 9.08–8.62 (m, 1 H), 8.44–7.85 (m, 2 H), 5.44–4.96 (m, 1 H), 2.60–2.23 (m, 1 H), 2.07–1.56 (m, 1 H), 1.45–0.99 (m, 1 H). ^13^C NMR (125 MHz,DMSO-*d*_6_) *δ* = 174.2, 147.02, 143.78, 140.83, 125.83, 123.3, 120.9, 57.8, 34.3, 25.9.

#### 2.5.8. Poly (1-(3-trifloromethyl phenyl)-1*H*-123- triazol-4-yl) acrylate (3h)

0.421 g, 80% yield, ^1^H NMR (500 MHz, DMSO-*d*_6_) *δ* = 9.13–8.56 (m, 1 H), 8.36–7.92 (m, 2 H), 7.88–7.50 (m, 2 H), 5.38–4.92 (m, 2 H), 2.48–2.11 (m, 1 H), 1.94–1.50 (m, 2 H), 1.37–0.95 (m, 2 H). ^13^C NMR (125 MHz, DMSO-*d*_6_) *δ* = 174.1, 143.4, 137.3, 131.6, 127.2, 125.6, 124.2, 123.6, 122.8, 120.6, 117.1, 57.6, 35.2, 26.6.

## 3. Results and discussion

**Scheme 1.**
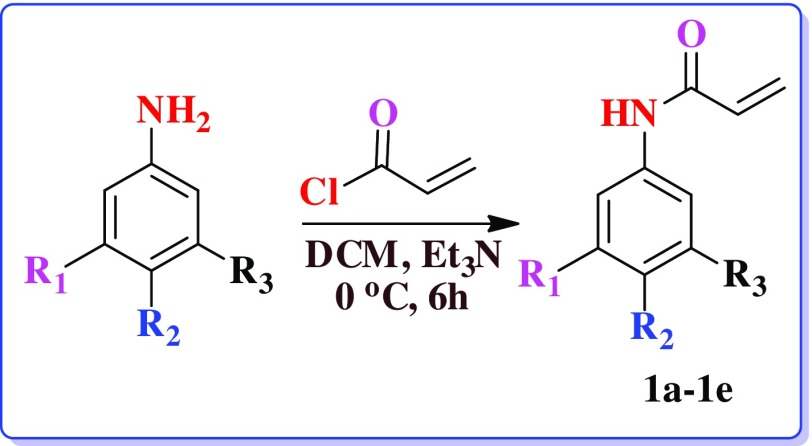
Synthesis of *N*–phenyl acrylamide.

Attempt was made in this study to synthesize acrylate monomers having various substituents. Initially, various reactions parameters were examined using different kinds of reaction conditions for synthesis of acrylate monomers (Table 1). For the reaction between aromatic amine and acryloyl chloride, we attempted reaction with triethyl amine as a base and DCM as solvent to get the product as substituted acrylamide (Scheme 1). By using the above mentioned optimized method, five aromatic substituted acrylamide were synthesized. The substitution was done with energetic groups such as –NO_2_, and –CF_3._ The structure of the aromatic acrylamide monomers was characterized. Yield of the nitro containing polymers are observed to be very less due to their electron withdrawing nature. In–CF_3_ molecule when –H is replaced by fluorine, density of the hydrocarbon will be increased. While –CF_3_ possessing monomers yield is high. Finally, the desired products of monomers were obtained after optimizing the conditions with good amount of yield. The structure and the yield of various monomers are described below in Table 2.

**Table 1.** Different conditions to optimized the reaction.

Compound	Reaction mixture	Temperature (°C)	Time (h)	Yield (%)
1(b)	Triethylamine/ethyl methyl ketone	0 for 1 h and 25 for 5 h	6	57
	K_2_CO_3_/acetone	0 for 1 h and 25 for 5 h	6	55
	Triethylamine/dichloromethane	0 for 1 h and 25 for 5 h	6	65

**Table 2.** Structure and yield of compounds (1a–1e).

S. No.	Compound	Yield (%)
1a	R_2_=H, R_1_=NO_2_, R_3_=H	75
1b	R_2_=NO_2_, R_1_=H, R_3_=H	65
1c	R_2_=H, R_1_=CF_3_, R_3_=H	78
1d	R_2_=NO_2_, R_3_=CF_3_, R_1_=H	80
1e	R_2_=NO_2_, R_3_=NO_2_, R_1_=H	65

**Scheme 2.**
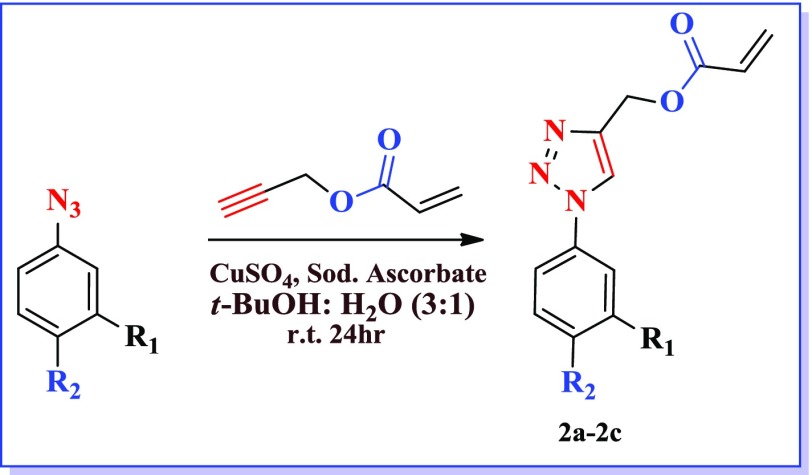
Synthesis of (phenyl-1*H*-1,2,3-triazol-4-yl) acrylate.

After synthesizing energetic group embedded acrylamide monomers, we ventured upon to synthesize triazole based acrylesters. The formation of the acrylic monomers (2a–2c) was accomplished by using copper-catalyzed 13-dipolar cycloaddition of the organic azides [26] with propargyl acrylate. The reaction was carried out under mild conditions for 8–10 h at room temperature in water and *t*-butanol mixture (Scheme 2) and structure of different triazole acrylate monomers are mentioned in Table 3.

**Table 3.** Structure and yield of compounds (2a–2c).

S. No.	Compound	Yield (%)
2a	R_2_=H, R_1_=NO_2_	68
2b	R_2_=NO_2_, R_1_=H	71
2c	R_2_=H, R_1_=CF_3_	66

### 3.1. Polymerization

**Scheme 3.**
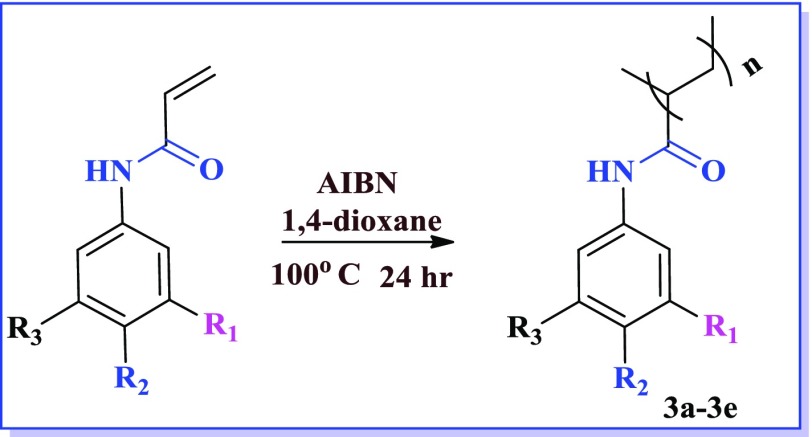
Synthesis of poly (*N*-phenyl) acrylamide.

**Scheme 4.**
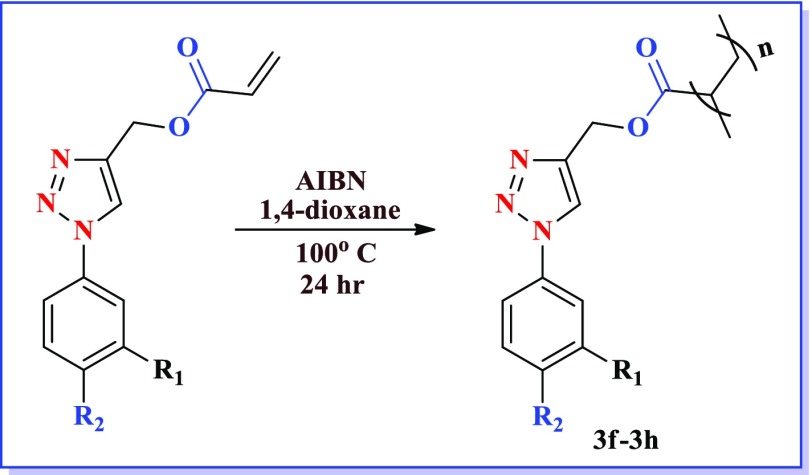
Synthesis of poly (phenyl-1*H*-1,2,3-triazol-4-yl) acrylate.

After accomplishing acrylamide (1a–1e) and acrylate monomers (2a–2c), we attempted radical polymerization reaction by various methods. In polymerization step, different conditions were endeavoured (Table 4), but the yield of the desired polymer was low. After several efforts, changing the solvent to dioxane & AIBN as radical initiator, better yields were obtained as listed in Table 5. After 12 h the color of the reaction mixture turned brown & then product formed were precipitated in the methanol. The yield of the polymerization ranged from good to better. The good yield of the –CF_3_ possessing polymer was observed as mentioned above. Reactions of the acrylamide and acrylate polymers are shown in Schemes 3 and 4.

**Table 4.** Different conditions to optimized polymer reaction.

Compound	Solvent	Free radical initiator	Temperature (°C) & Time (h)	Yield (%)
3(b)	Toluene	Benzoyl peroxide	110 & 24	20
	Tetrahydrofuran	AIBN	70 & 24	–
	2-Butanone	AIBN	75 & 24	30
	14-Dioxane	AIBN	100 & 24	64

**Table 5.** Structure and yield of compounds (3a–3e).

S. No.	Compound	Yield (%)
3a	R_2_=H,R_1_=NO_2_, R_3_=H	62
3b	R_2_=NO_2_, R_1_=H, R_3_=H	64
3c	R_2_=H, R_1_=CF_3_, R_3_=H	75
3d	R_2_=NO_2_, R_3_=CF_3_, R_1_=H	58
3e	R_2_=NO_2_, R_3_=NO_2_, R_1_=H	51

The triazole based polymers (3f–3h) were synthesized by the same procedure and the yields of the polymers are mentioned in the Table 6.

**Table 6.** Structure and yield of compounds (3f–3h).

S. No.	Compound	Yield (%)
3f	R_2_=H, R_1_=NO_2_	62
3g	R_2_=NO_2_, R_1_=H	63
3h	R_2_=H, R_1_=CF_3_	80

Polymerization reaction for substrate (1b) was also attempted using microwave. Wherein, monomer was dissolved in 14 dioxane, followed by addition of AIBN to the reaction mixture under inert atmosphere at 80 °C, 50 W varying the heating duration from 10, 15, 30 and 60 min. After 60 min. the polymerization reaction was completed, but the yield obtained was not equivalent with that of conventional heating method as described above and also scaling up of this reaction was difficult. Hence we concluded that conventional heating gave better yield of poly nitro acrylamide as compared to microwave heating.

Attempts to do polymerization at lower temperature (less than 70 °C) showed lower yield because fewer radicals were generated at low temperature leading to incomplete conversion to polymer. Generally, polymerization step is reported to be carried out at 70 °C, but in our case presence of nitro and di nitro polymerization took place at relatively higher temperature ~100 °C. Increasing the number of nitro group in aromatic ring retards the reactivity of monomer. In 35 dinitro acrylamide (3e) presence of two nitro groups lowers the yield of the polymer. On the other hand, 246 trinitro acrylamide did not show polymerization reaction due to the presence of three nitro groups which act as scavenger to free radicals thereby inhibiting the polymerization reaction.[27]

**Figure 1.**
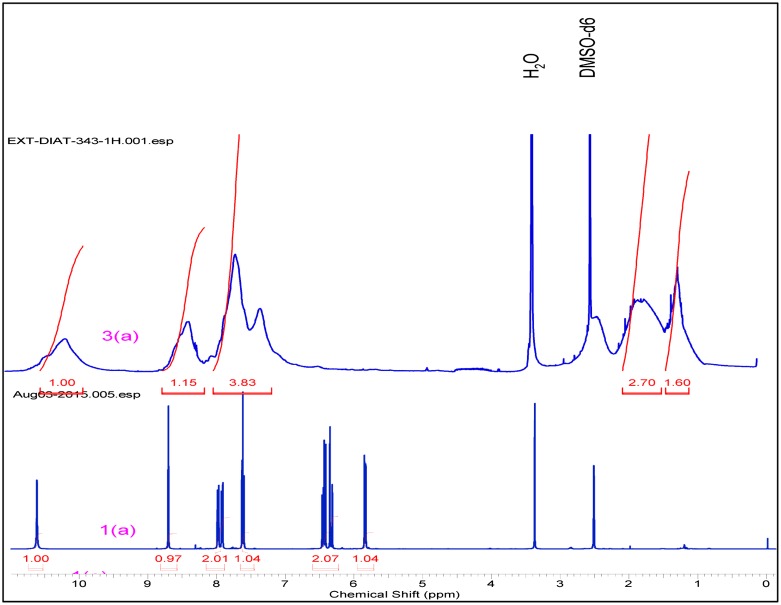
^1^H NMR of monomer 1(a) & polymer 3(a).

**Figure 2.**
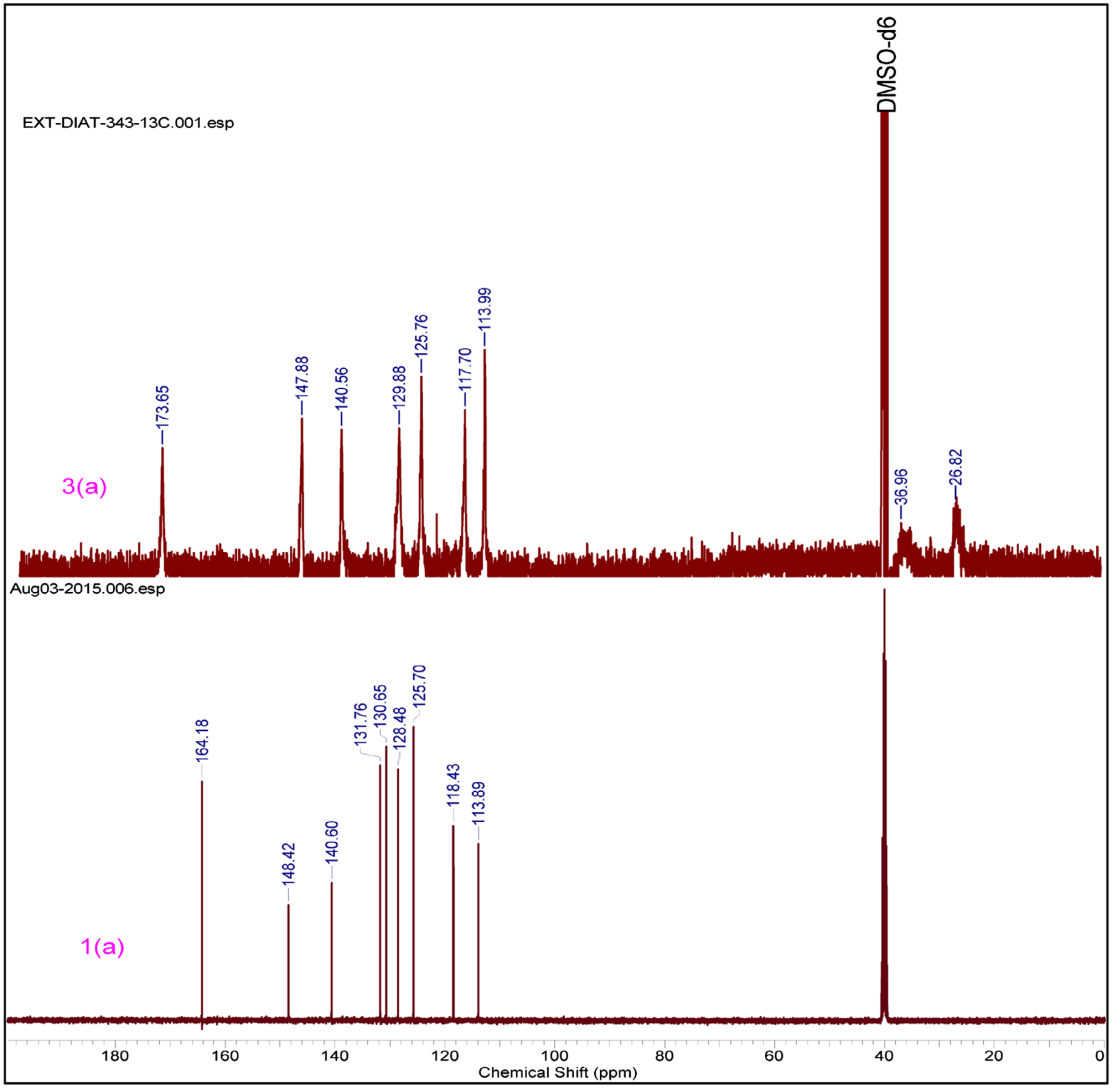
^13^C NMR of monomer 1(a) & polymer 3(a).

The structure of compounds (3a–3h) was confirmed by different techniques. FTIR spectra of compound (1a–1e) exhibit the presence of peaks in the range cm^−^^1^, 1665–1690 (–CONH), 1493–1595(Aromatic –C=C–), 1327& 1520(–NO_2_), ~1240 (–C=C). During polymerization a new peak was generated at ~2940 cm^−^^1^, which confirms that the double bond peaks has been reduced to as happening during the polymerization step was successfully done. (Supporting Information) In similar way, ^1^H NMR spectra of polymer (3a–3h) ~6.50–6.25(s, 1H) and ~5.8(s, 1H) peaks disappeared and a new peak ~1.26–2.55(–CH_2_) was observed, this peak indicates that –CH = CH_2_ changes into methylene group. In carbon NMR, two additional new peaks were observed around 25 and 37 ppm and corresponding to saturated carbon atoms. (Figure 1). In Figure 2, carbon NMR depicts two additional new peaks around 26 and 37 ppm which are assigned to saturated carbon atoms. (Supporting Information)

**Figure 3.**
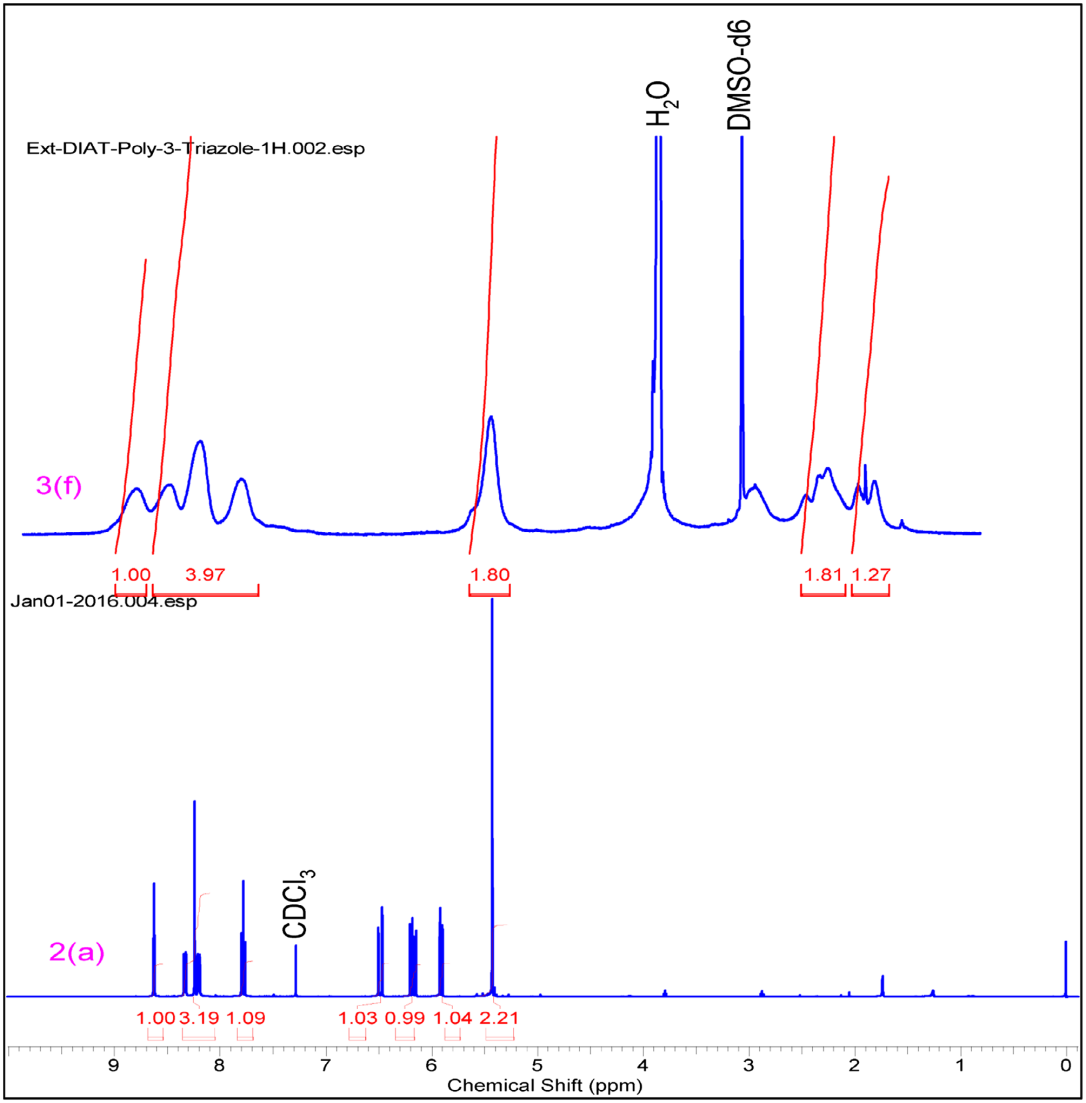
^1^H NMR of triazole monomer 2(a) & polymer 3(f).

**Figure 4.**
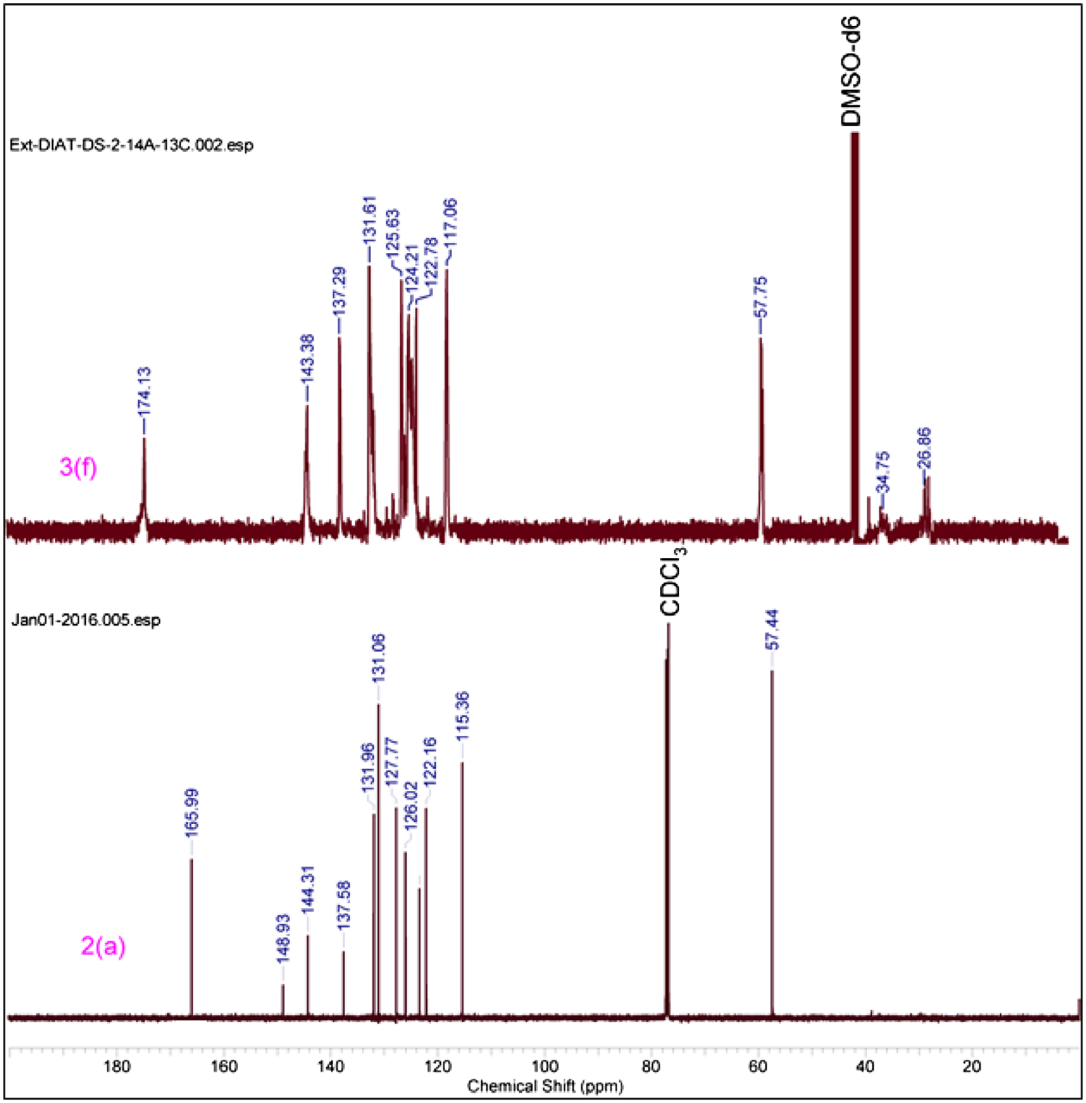
^13^C NMR of triazole monomer 2(a) & polymer 3(f).

In IR spectrum formation of triazole based monomer (3f–3h), 2100 cm^−^^1^ strong absorption peak of azide –N_3_ disappeared when it reacted with propargyl acrylate and the triazole and propargyl acrylate peak appeared at ~1647 and ~1728 cm^−^^1^ respectively. Cu catalyzed [2+3] cycloadditions reaction mechanism is involved in this reaction. Similar results were obtained from triazole polymers (Figures 3 and 4).

### 3.2. Molecular weight

Molecular weight of polymers was determined by GPC. Number and weight average molecular weight, polydispersity index were determined and are represented in Table 7. The average polydispersity indexes of the polymers are in the range of 1.00 to 1.74. Molecular weights of nitro based polymers (3a–3h) (2700–9000) are in good agreement with the reports present in the literature.[28] Nitro group acts as a radical scavenger and hence the presence of nitro groups in polymer backbone retards the growth of polymer chain. Further, the decrease in polymer weight in 3(c) and 3 (d) was observed. The presence of bulky and electron withdrawing group (–CF_3_) in both the monomers prevents attaining high molecular weight, which may be due to reduced approach of monomers and also the electron withdrawing effect weakening –NH bond for favored chain transfer to monomer. In the case of polymer (3 h) even in presence of –CF_3_ group, the molecular weight is enhanced. The presence of –CF_3_ group being far away from the double bond may not interfere by reducing the reactivity. Further, the hydrogen bonding increases the viscosity. Under this condition, the rate of termination of polymer radicals becomes diffusion controlled. As highly viscous medium is present the termination rate becomes very low making the overall rate of increase of polymerization higher (Equation 1). Further, the monomer being a small molecule, the mobility is not much affected and molecular weight continues to increase. Moreover it reduces the rate of termination of polymer radicals which also leads to increase of molecular weight. The rate of polymerization *R*_p_ is expressed as:

(1) $$R_{\text{p}}=k_{\text{p}}\left[M\right]\left(\frac{fk_{\text{d}}I}{k_{\text{t}}}\right)\frac{1}{2}$$

**Table 7.** Molecular weight of polymers.

Polymer	Average *M*_w_	Average *M*_n_	PDI *M*_w_/*M*_n_
3(a)	5448	4097	1.33
3(b)	8314	8314	1.00
3(c)	2791	2791	1.00
3(d)	2791	2791	1.00
3(f)	8985	5146	1.74
3(g)	3877	2216	1.74
3(h)	48,938	31,798	1.53

Where M is the monomer, *f* is the mole fraction of free radical initiator which initiate the polymerization, I is the initiator, *k*_p_, *k*_d_, and *k*_t_ are the constants for chain propagation, initiator dissociation, and termination.

### 3.3. Thermal studies

**Figure 5.**
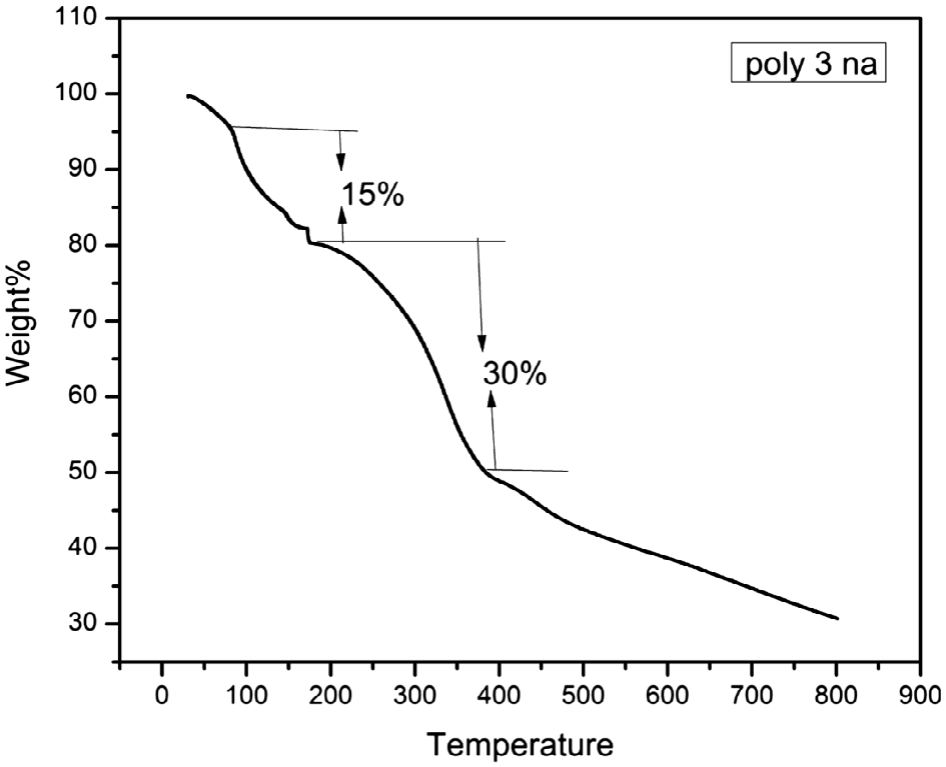
TGA diffractogram of poly 3NA (3a).

**Figure 6.**
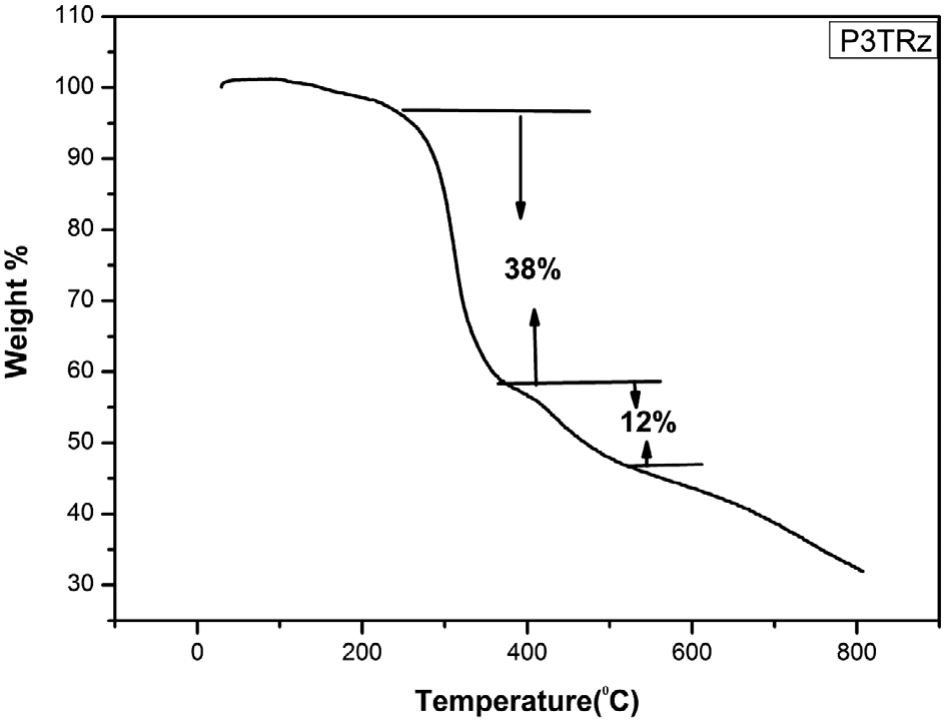
TGA diffractogram of poly 3TrZ (3f).

The thermograms of all the synthesized polymers except poly 3NA (3a), poly 3TrZ(3f) and poly 4 TrZ (3 g) clearly indicates that they undergo single stage decomposition. The two stage decomposition of poly 3NA, poly 3TrZ and poly 4TrZ can be explained (Figures 5 and 6) by taking into consideration the following mechanism. The first step of decomposition implicates the loss of CO_2_, breaking of the weak linkage in the backbone of the polymer thereby leading the low molecular moiety to volatilize. The second step is mainly due to loss of the benzene ring and rupture of the pendant group (–NO_2_) from the backbone leading to the cleavage of the main chain.[29] TGA curves for these polymer samples are depicted in supporting information.

**Scheme 5.**
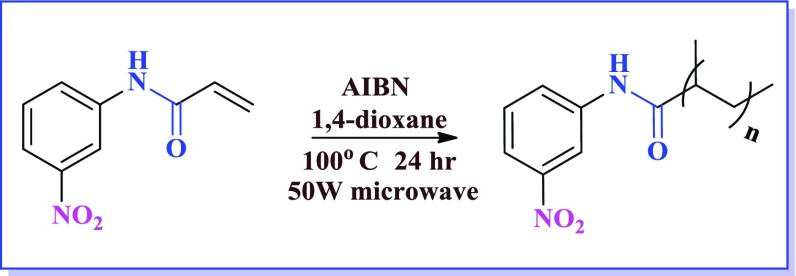
Synthesis of poly (3 nitro-phenyl) acrylamide 3(a).

TGA curves shows the variation of enthalpy (∆H) (3a–3h) from 81 to 951.59 J/g. Nitrogen containing (–NO_2_, N_3_ and triazole) compounds are found to possess high amount of energy, so incorporation of nitro and triazole ring in polymer backbone will led to high amount of heat in the form of exothermic reaction. Poly 4TrZ (3g) showed maximum enthalpy (∆H) i.e. 951.59 J/g, which is mainly due to presence of both nitro group and triazole ring.

The glass transition temperatures (*T*_g_) of the synthesized polymers are evaluated using DSC and represented in the Table 8. *T*_g_ values varies in the range from 78 to 125 °C. The observed suppression of *T*_*g*_ values is due to presence of electron withdrawing groups (–NO_2_, –CF_3_) in the polymer backbone.[18]

**Table 8.** Thermal studies of polymers.

Polymer	*Tg* (°C)	∆H (J/g)	Weight loss (%)	Temperature range (°C)
3(a)	83.66	93.18	15	95–174
			30	174–377
3(b)	96.53	149.47	38	94.77–411
3(c)	86.49	237.09	87	156–235
			10	235–417
3(d)	90.33	81.37	37	94–436
3(e)	78.15	298.93	41	212–399
3(f)	78.10	779.60	38	244.73–369.9
			12	369.9–525.99
3(g)	125	951.59	33	256–362
			12	362–501
3(h)	90.5	420.41	80	288.4–452

### 3.4. Oxygen balance (O.B.)

Oxygen balance is an important parameter to explain the sensitivity and degree of explosion, where an explosive material can be oxidized. Oxygen balance (O.B.) is expressed as,

(2) $$\text{O}\text{.B}\text{.}\left(\Omega \right)\%=\frac{-1600}{\text{Molecular weight of compound}}\left(2X+\frac{Y}{2}+M-Z\right)$$

Where *X* = number of atoms of carbon, *Y* = number of atoms of hydrogen, *Z* = number of atoms of oxygen and *M* = number of atoms of metal (if metallic oxide produced).

Mostly energetic molecules possess negative oxygen balance, it means these materials require oxygen to combust. Oxygen is needed to convert all C into CO_2_ and H into H_2_O. O.B. of the polymers which we have described in the present work lies in the range of −111.37 to −174.95% (Table 9) which is almost similar to energetic binders which are used in propellant formulations such as GAP(−121%), Poly-BAMO(−124%), Poly-AMMO(−170%), and Poly-NIMMO (−114%).[30] But polymers which we are describing here possess explosophores groups such as nitro and triazole, hence these types of polymers are comparable to other energetic materials.

**Table 9.** O.B. of polymers.

Polymer	%Nitrogen content	O.B. (%)
3(a)	14.57	−174.95
3(b)	14.57	−174.95
3(c)	6.50	−171.12
3(d)	10.76	−126.13
3(e)	17.72	−111.37
3(f)	20.40	−145.94
3(g)	20.40	−145.94
3(h)	14.14	−156.18

## 4. Conclusions

Herein, we report the synthesis of novel energetic acrylamide/acrylate polymers which consist of energetic groups like –NO_2_,–CF_3_, and nitropheyl substituted triazole. Generally, the synthesis of these polymers may be difficult under normal conditions because of electron withdrawing nature. We have adopted a simple methodology to synthesize these molecules. These molecules demonstrate high heat release and better thermal stability. In addition, low value of glass transition temperature and increase in enthalpy of formation were found due to the increase of nitrogen content in polymer backbone. Molecular weights of the polymers were reduced due to the nitro group present in polymer network. Nitro group behaved like a scavenger in free radical polymerization. Nitro group containing polymers decomposed in two steps whereas others are found to be single step decomposition. All these properties of the nitro and triflouro containing polymers make then a good candidates for energetic applications.

## Disclosure statement

No potential conflict of interest was reported by the authors.

## Supplemental data

Supplemental data for this article can be accessed at http://dx.doi.org/10.1080/15685551.2016.1258977.

## Supplementary Material

TDMP_1258977_Supplementary_Material.pdf
