# Supplementary material for: Tailoring of energetic groups in acroyloyl polymers
Source: Des Monomers Polym. 2016 Nov 24;20(1):332–43. doi: 10.1080/15685551.2016.1258977 (PMC5812170; doi:10.1080/15685551.2016.1258977)
Supplement: TDMP_1258977_Supplementary_Material.pdf [file TDMP_A_1258977_SM7222.pdf]

Electronic supporting information

Tailoring of Energetic Groups in Acryloyl Polymers

Deepak Kumar, K. Durga Bhaskar Yamajala, Asit B.Samui and Shaibal Banerjee*

Organic Synthesis Lab, Department of Applied Chemistry,
Defence Institute of Advanced Technology (DU),
Girinagar, Pune-411025, India.

AUTHOR INFORMATION

Dr. Shaibal Banerjee

***Email:** shaibal.b2001@gmail.com

Tel: +9120 24304164

Contents

1. **FTIR, ^{1}H and ^{13}C NMR, spectra of 1(a) 3-4**
2. **FTIR, ^{1}H, ^{13}C NMR and DSC-TGA spectra of 3(a) 5-8**
3. **FTIR, ^{1}H and ^{13}C NMR, spectra of 1(b) 8-9**
4. **FTIR, ^{1}H, ^{13}C NMR and DSC-TGA spectra of 3(b) 9-12**
5. **FTIR, ^{1}H and ^{13}C NMR, spectra of 1(c) 12-13**
6. **FTIR, ^{1}H, ^{13}C NMR, and DSC-TGA spectra of 3(c) 14-16**
7. **FTIR, ^{1}H, and ^{13}C NMR, spectra of 1(d) 17-18**
8. **FTIR, ^{1}H, ^{13}C NMR, and DSC-TGA spectra of 3(d) 18-21**
9. **FTIR, ^{1}H and ^{13}C NMR, spectra of 1(e) 22-23**

10. FTIR, ^{1}H, ^{13}C NMR and DSC-TGA spectra of 3(e) **24-26**

11. FTIR, ^{1}H and ^{13}C NMR, spectra of 2(a) **27-28**

12. FTIR, ^{1}H, ^{13}C NMR and DSC-TGA spectra of 3(f) **29-31**

13. FTIR, ^{1}H, and ^{13}C NMR, spectra of 2(b) **32-33**

14. FTIR, ^{1}H, ^{13}C NMR, and DSC-TGA spectra of 3(g) **33-35**

15. FTIR, ^{1}H and ^{13}C NMR, spectra of 2(c) **36-37**

16. FTIR, ^{1}H, ^{13}C NMR and DSC-TGA spectra of 3(h) **38-41**

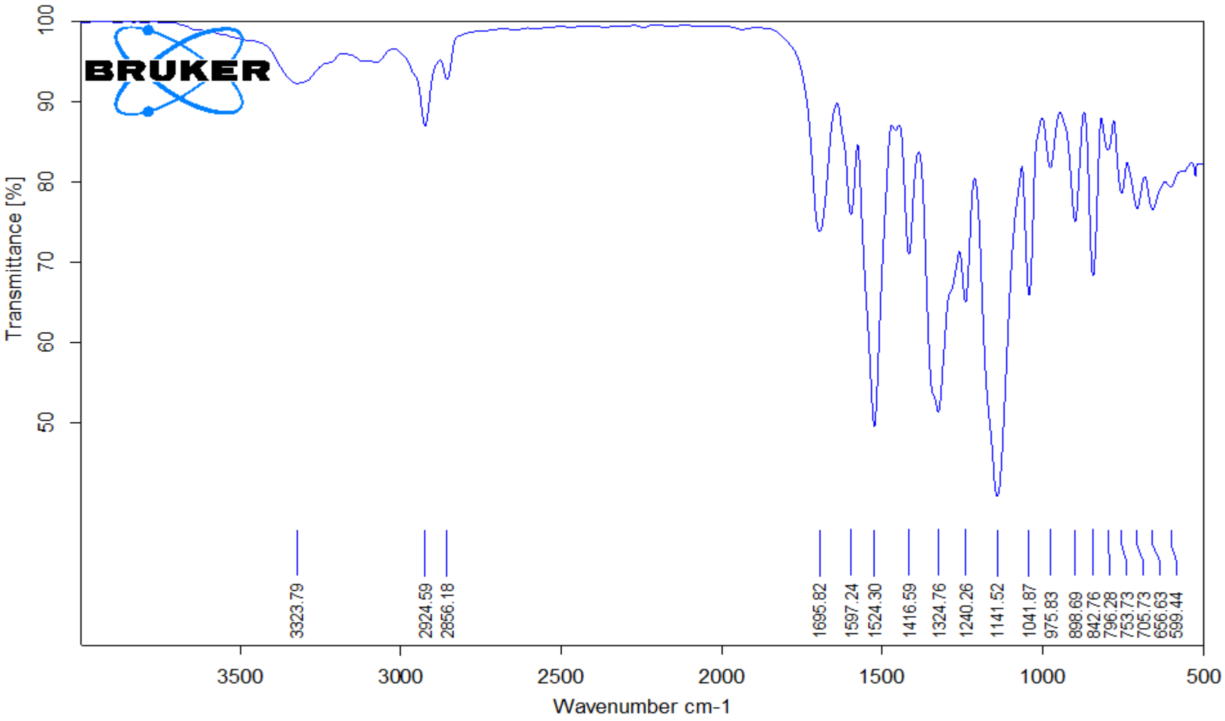

Figure S1. FTIR spectrum of 1(a)

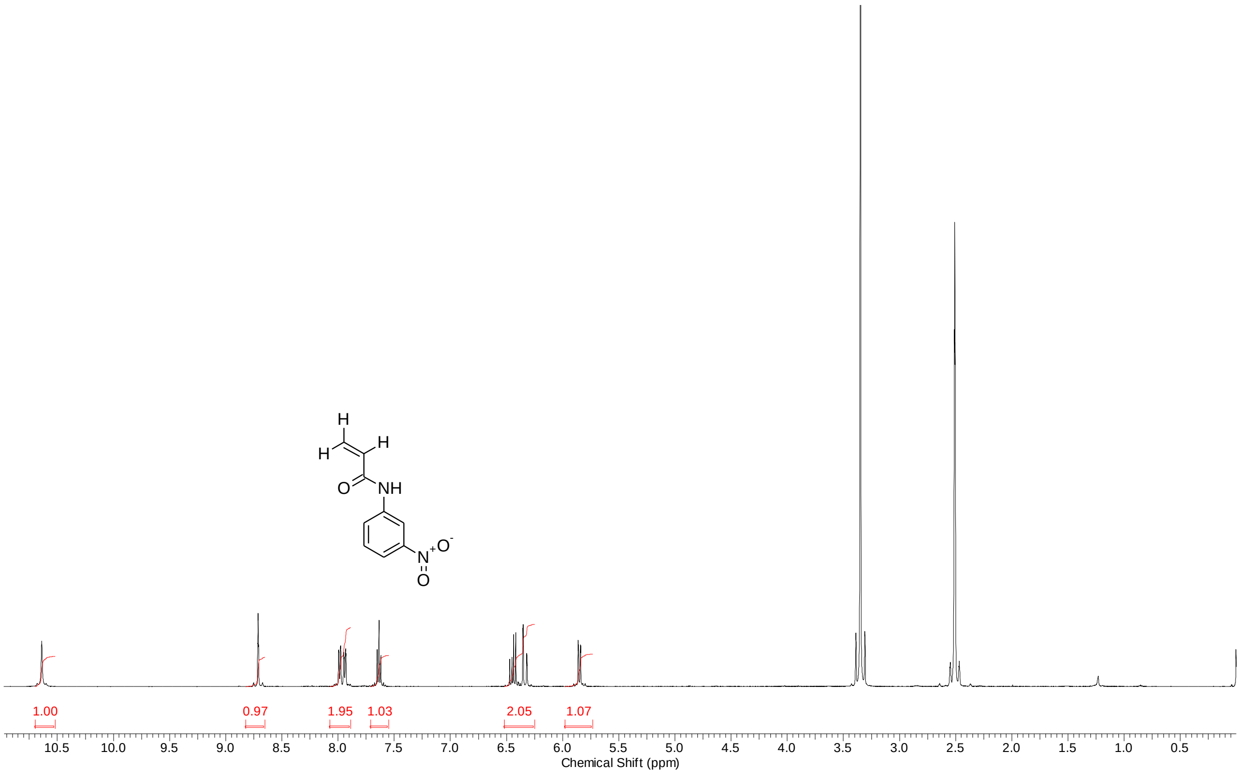

Figure S2. 1H NMR spectrum of 1(a)

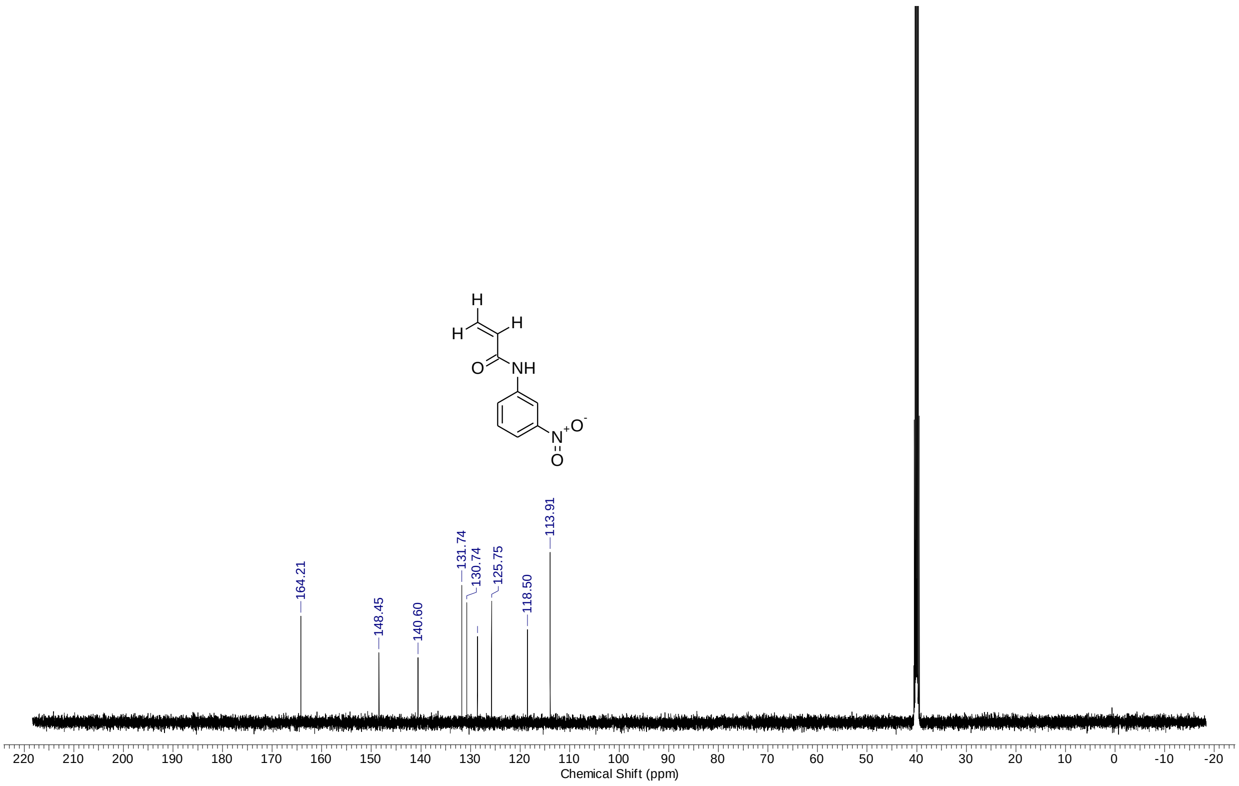

Figure S3. ^{13}C NMR spectrum of 1(a)

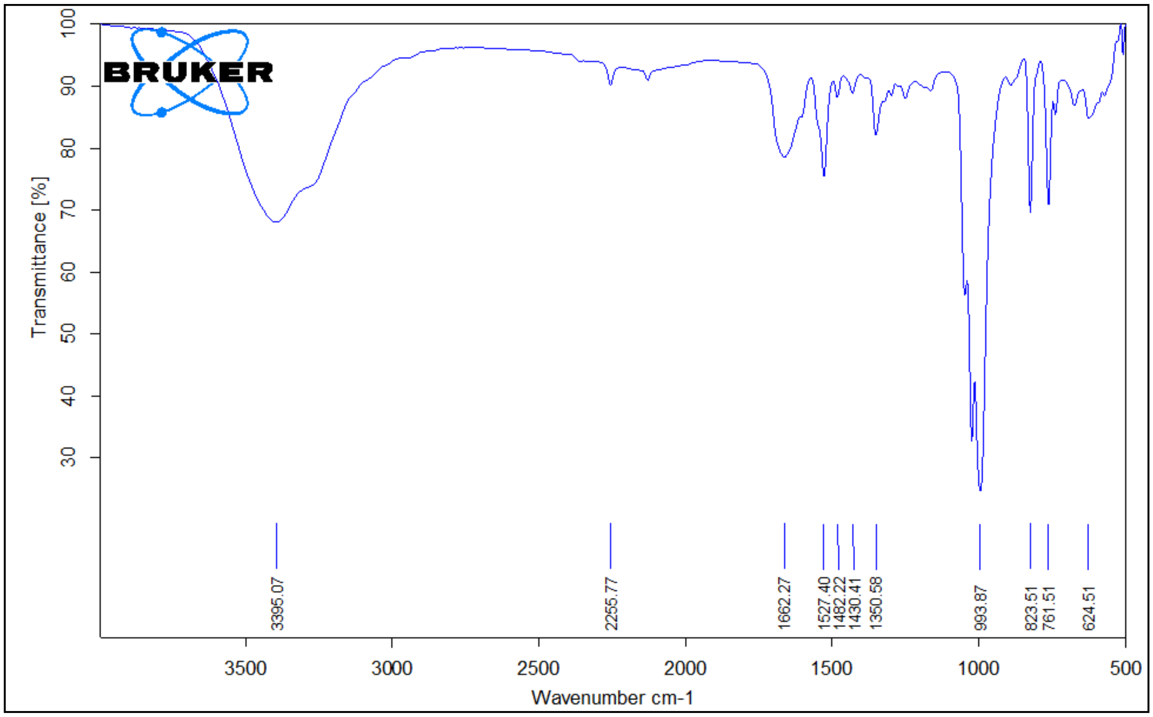

Figure S4. FTIR spectrum of 3(a)

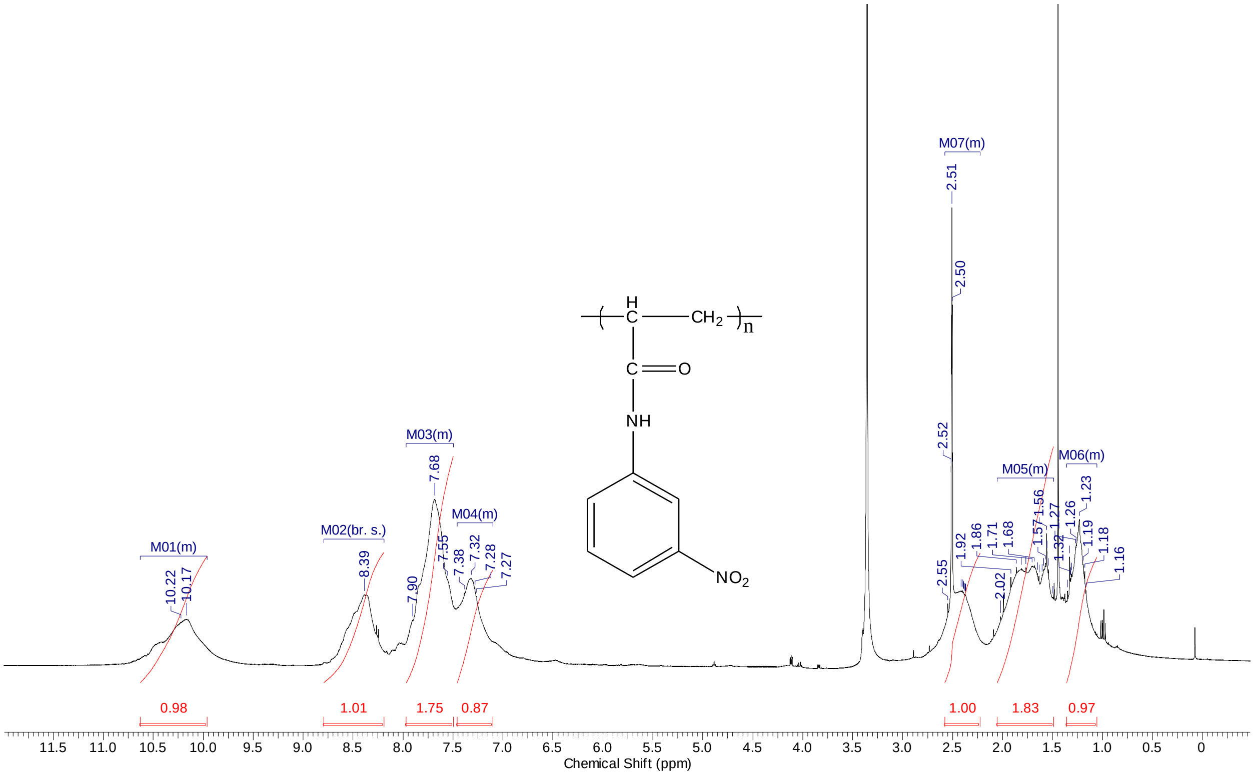

Figure S5. ^{1}H NMR of spectrum of 3(a)

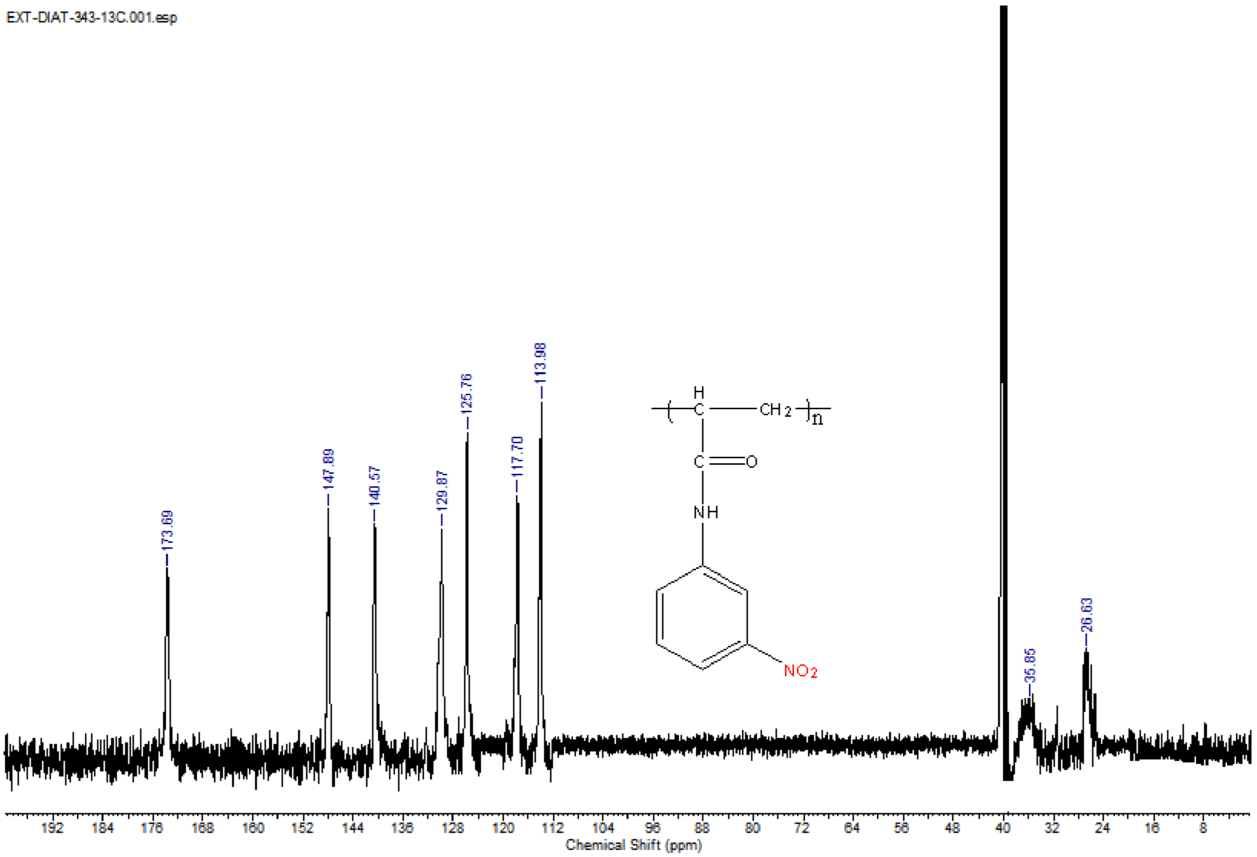

Figure S6. ^{13}**C NMR of spectrum of 3(a)**

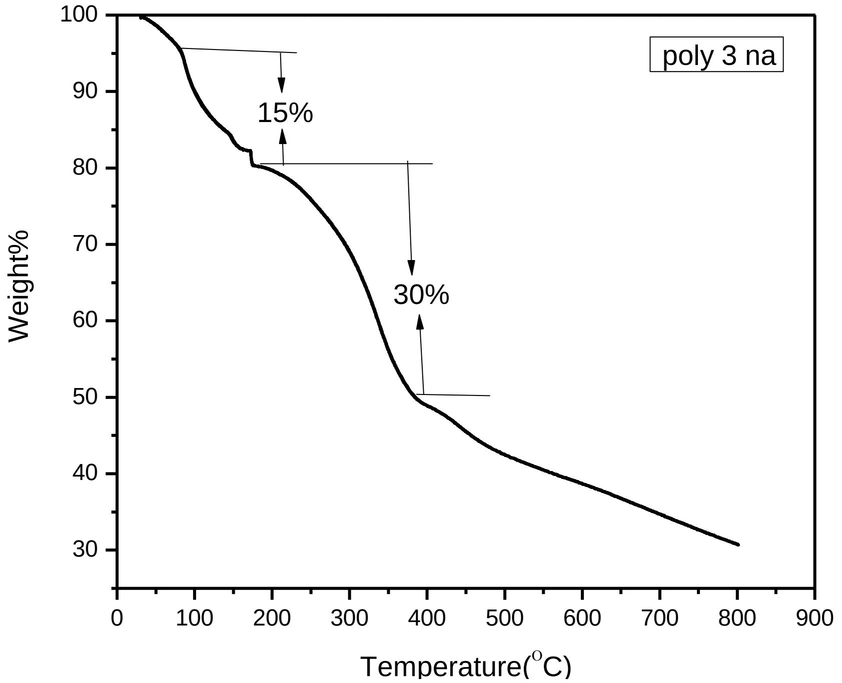

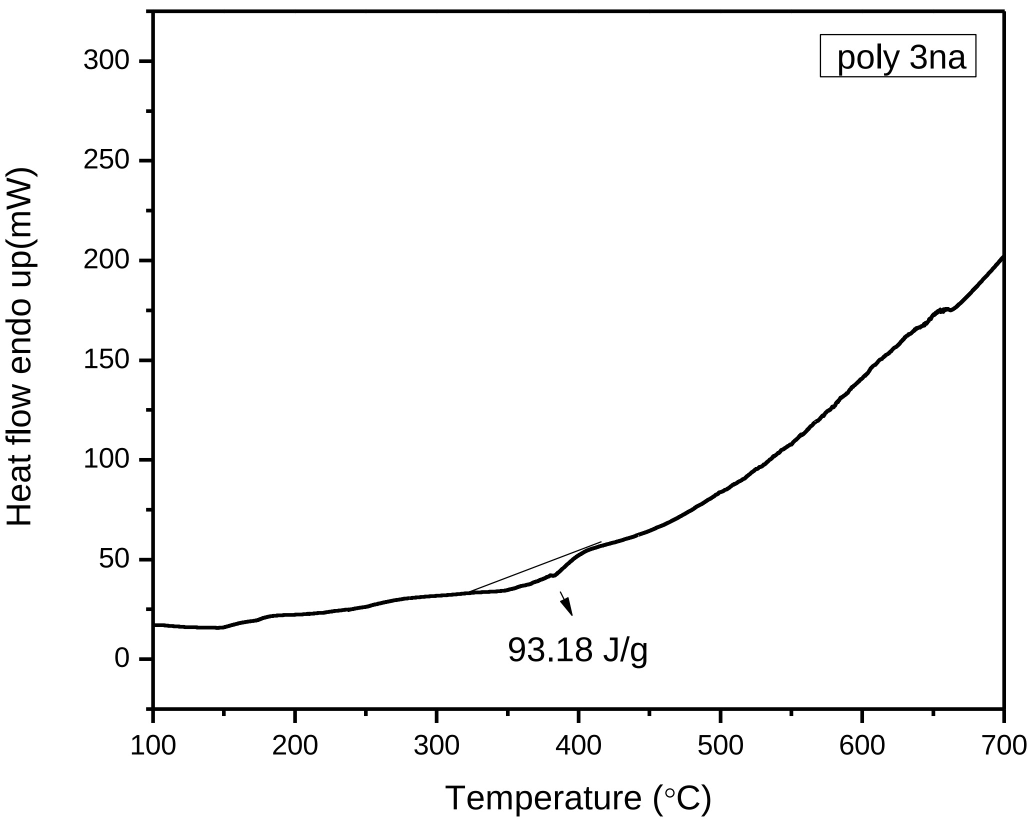

Figure S7 & Figure S8 . Thermal Studies of 3(a)

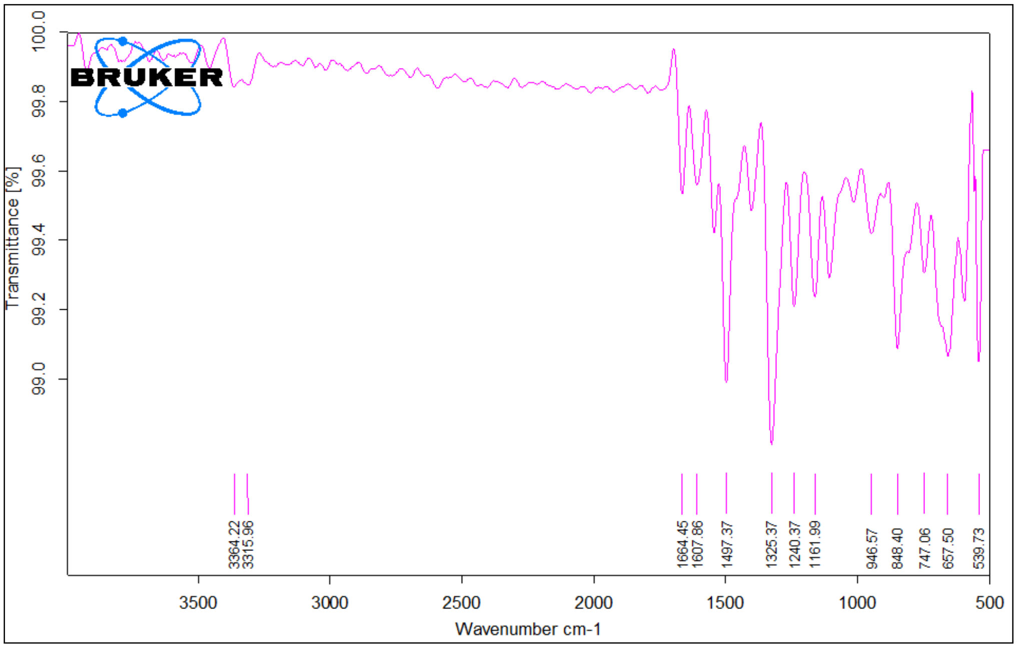

Figure S9. FTIR spectrum of 1(b)

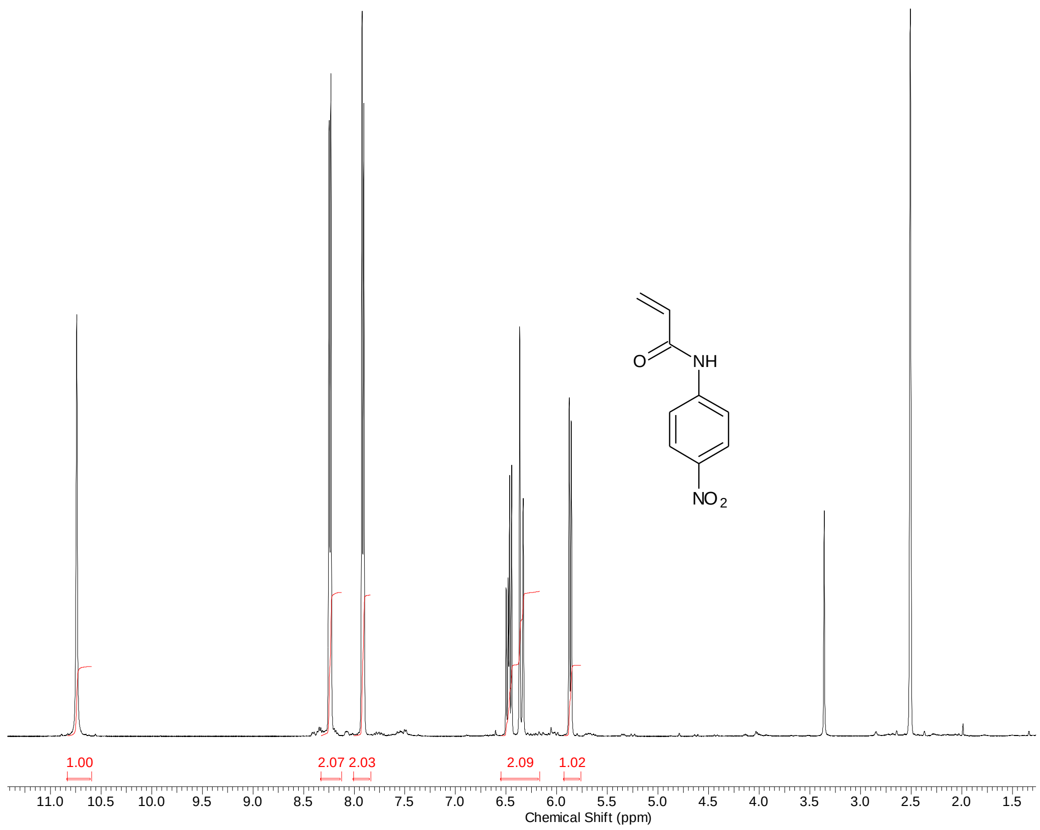

Figure S10. ^{1}H NMR spectrum of 1(b)

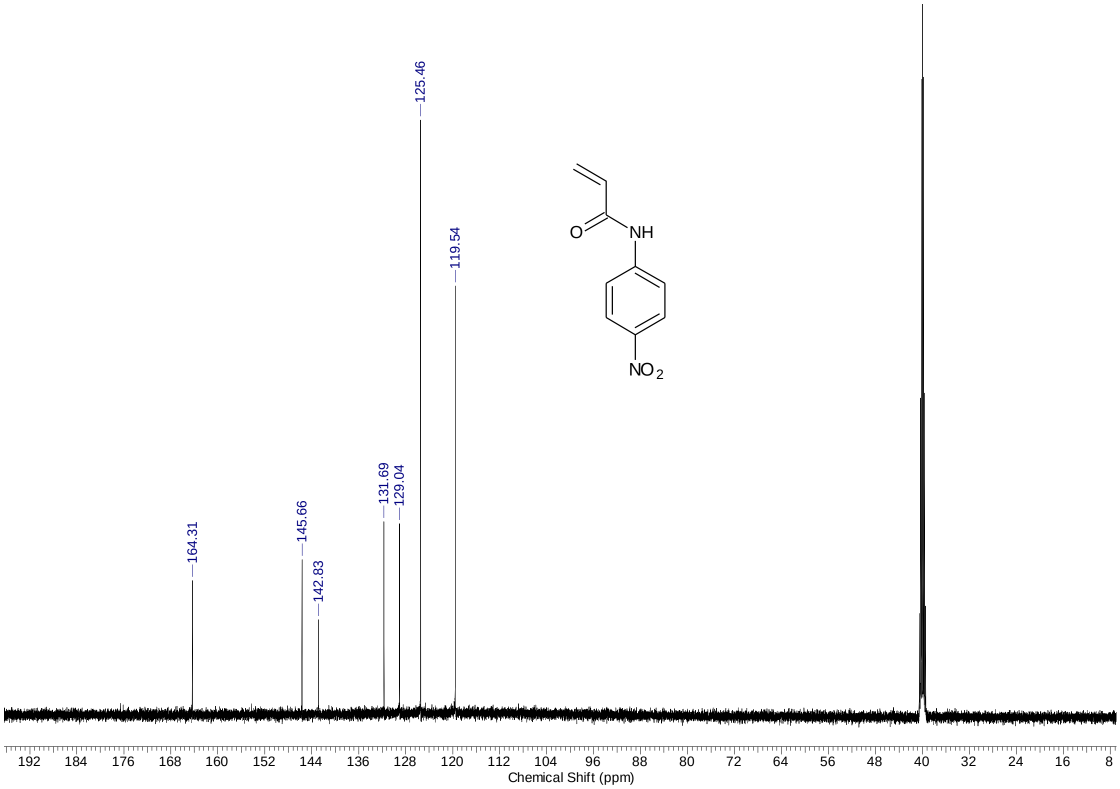

Figure S10. ^{13}C NMR spectrum of 1(b)

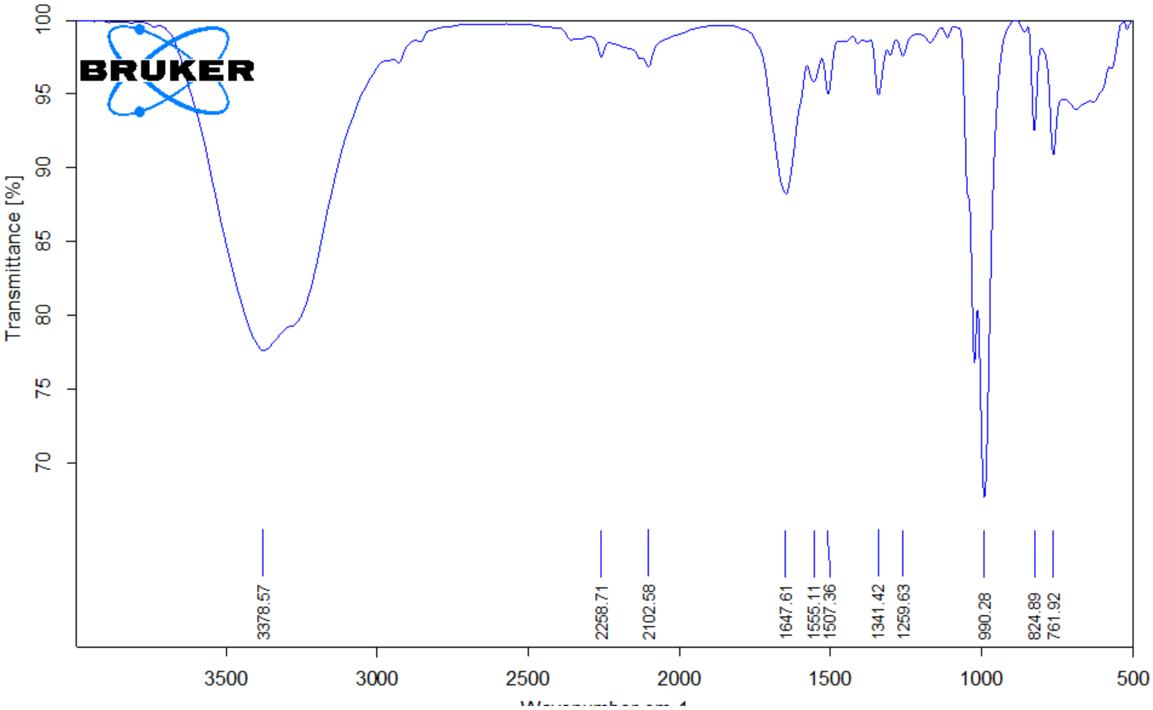

Figure S11. FTIR spectrum of 3(b)

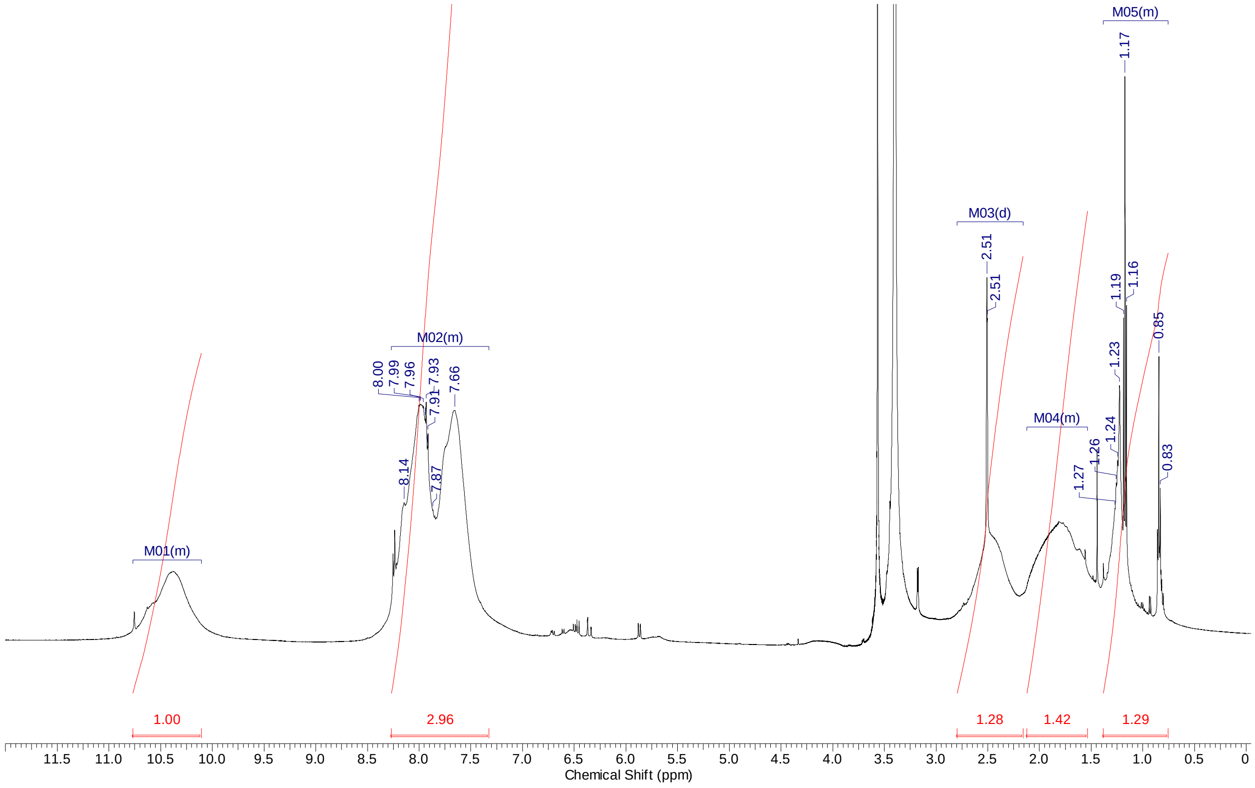

Figure S12. ^{1}H spectrum of 3(b)

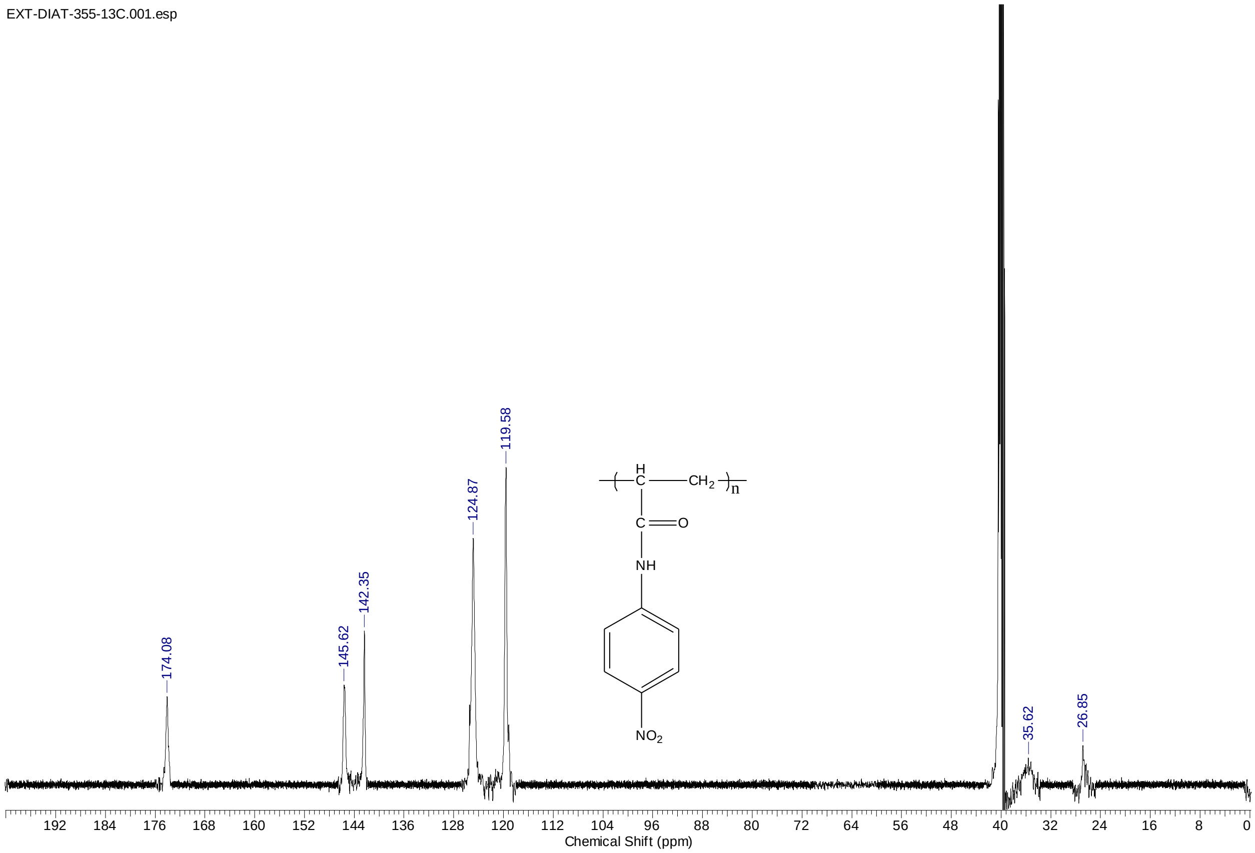

Figure S13. ^{13}C spectrum of 3(b)

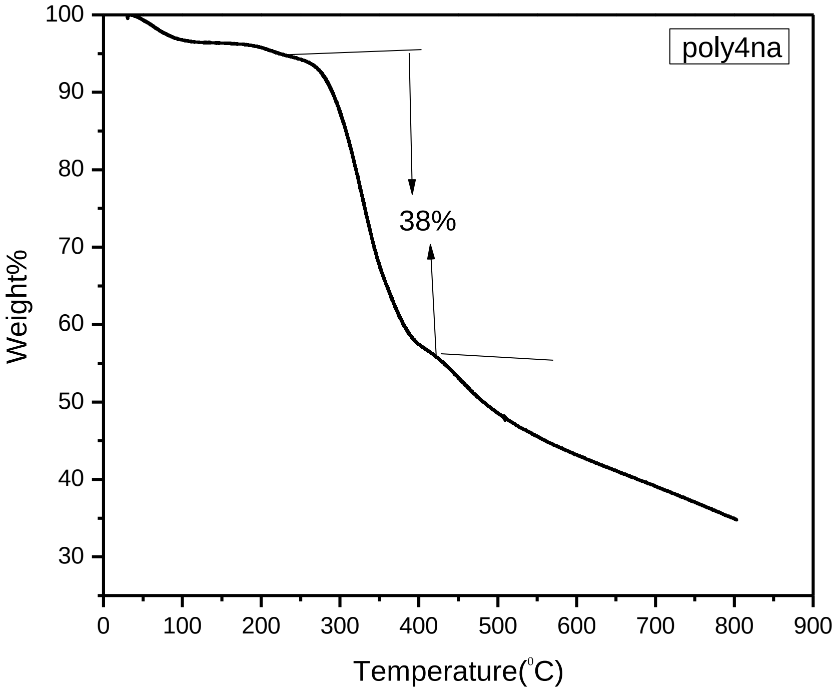

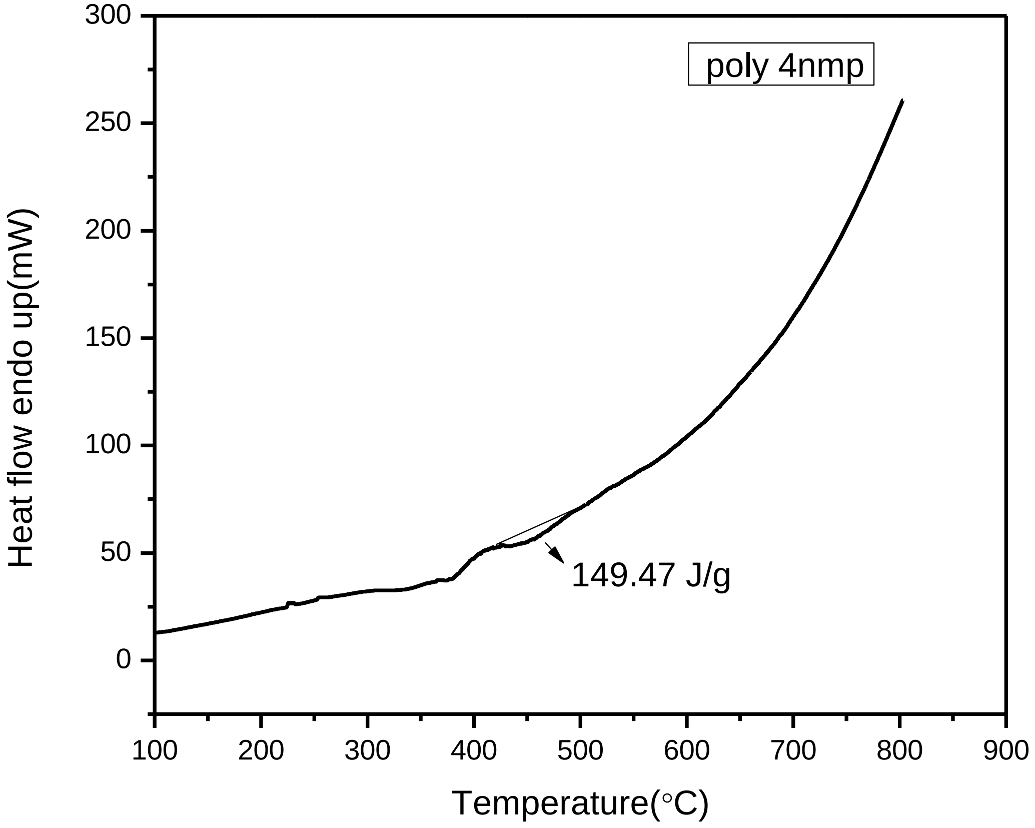

Figure S14& S15. Thermal studies of 3(b)

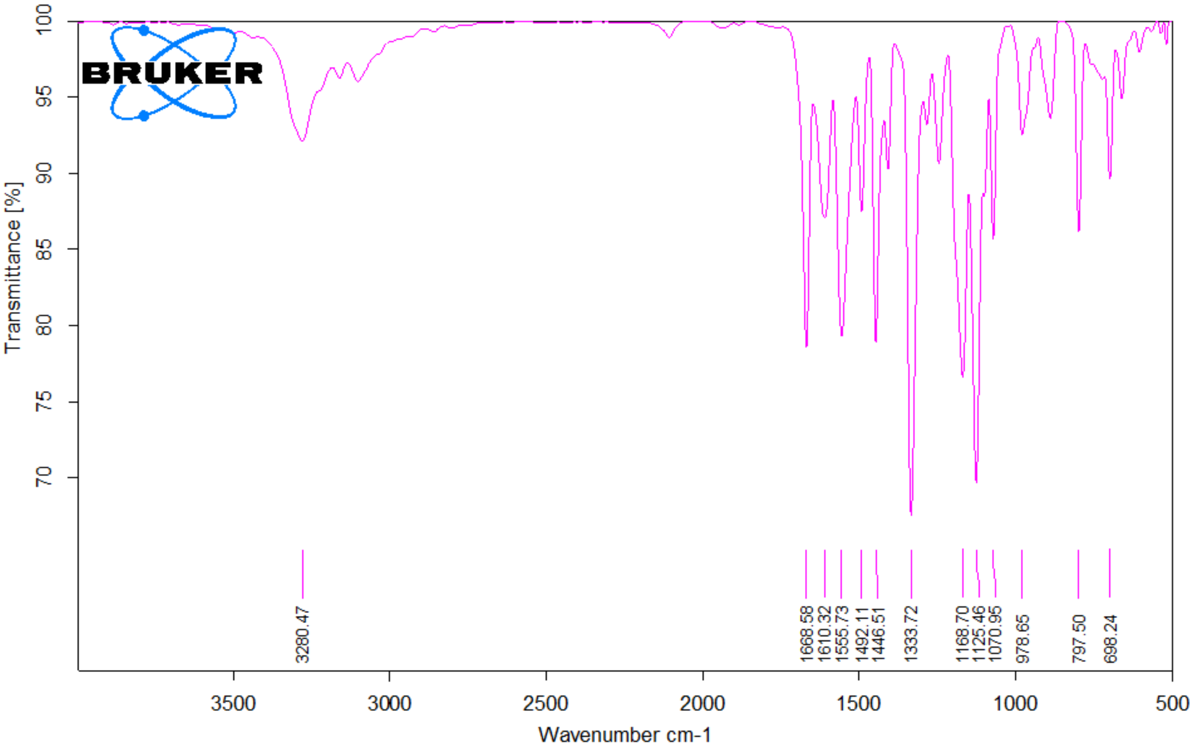

Figure S16. FTIR spectrum of 1(c)

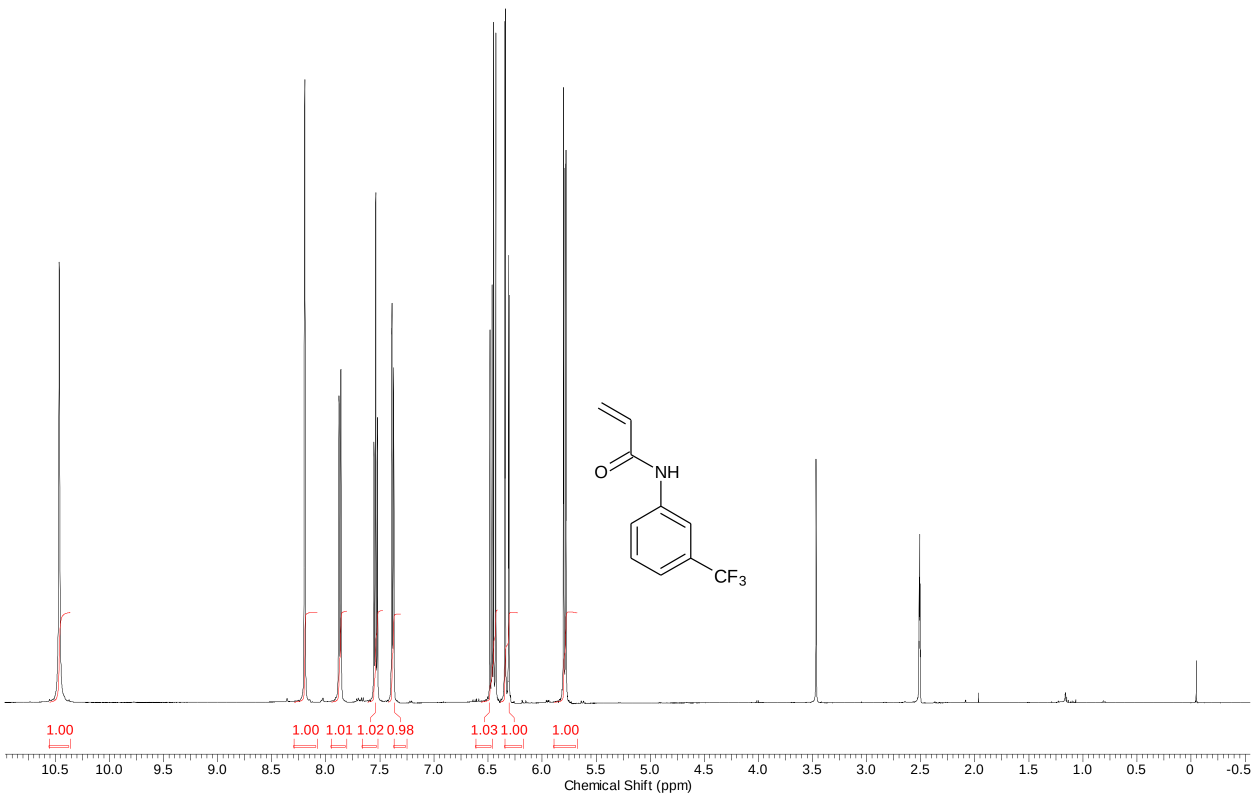

Figure S17. **^{1}H spectrum of 1(c)**

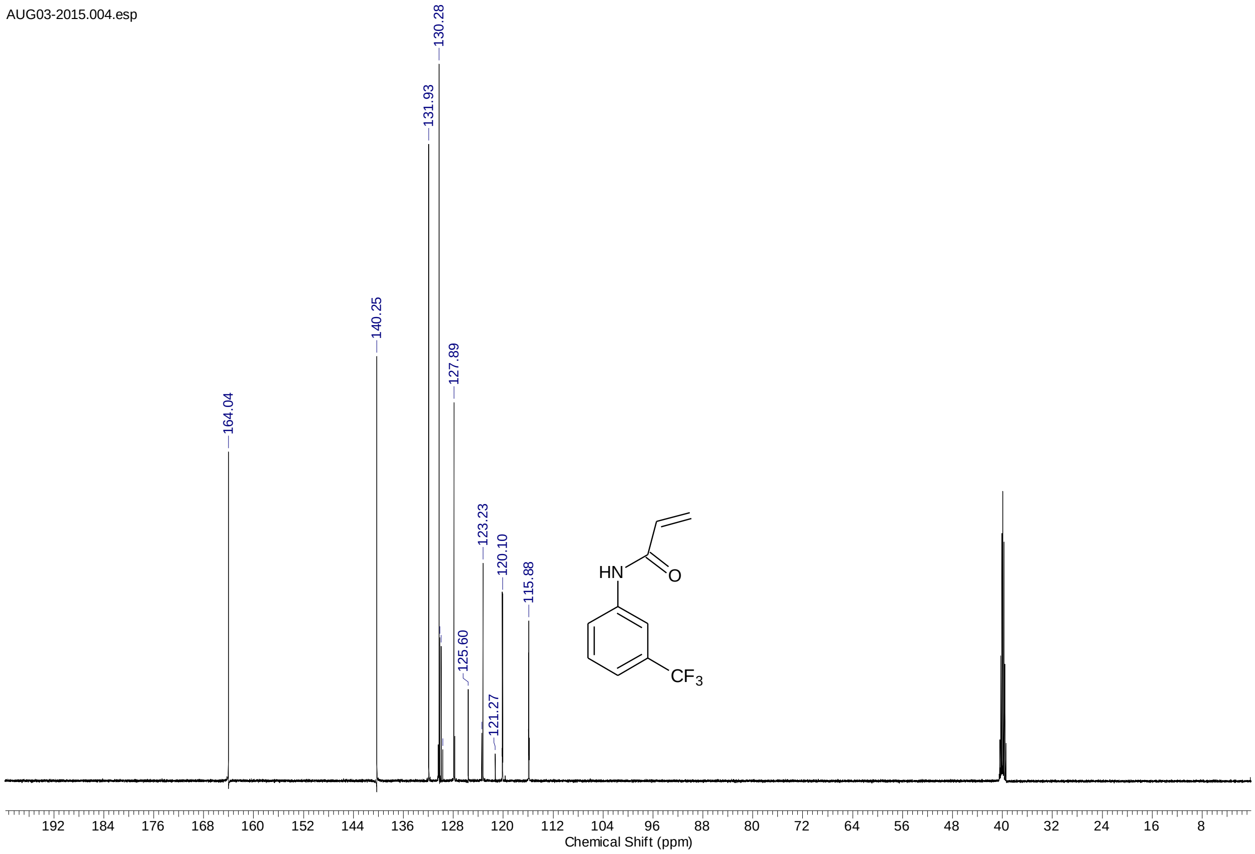

Figure S18. ^{13}**C spectrum of 1(c)**

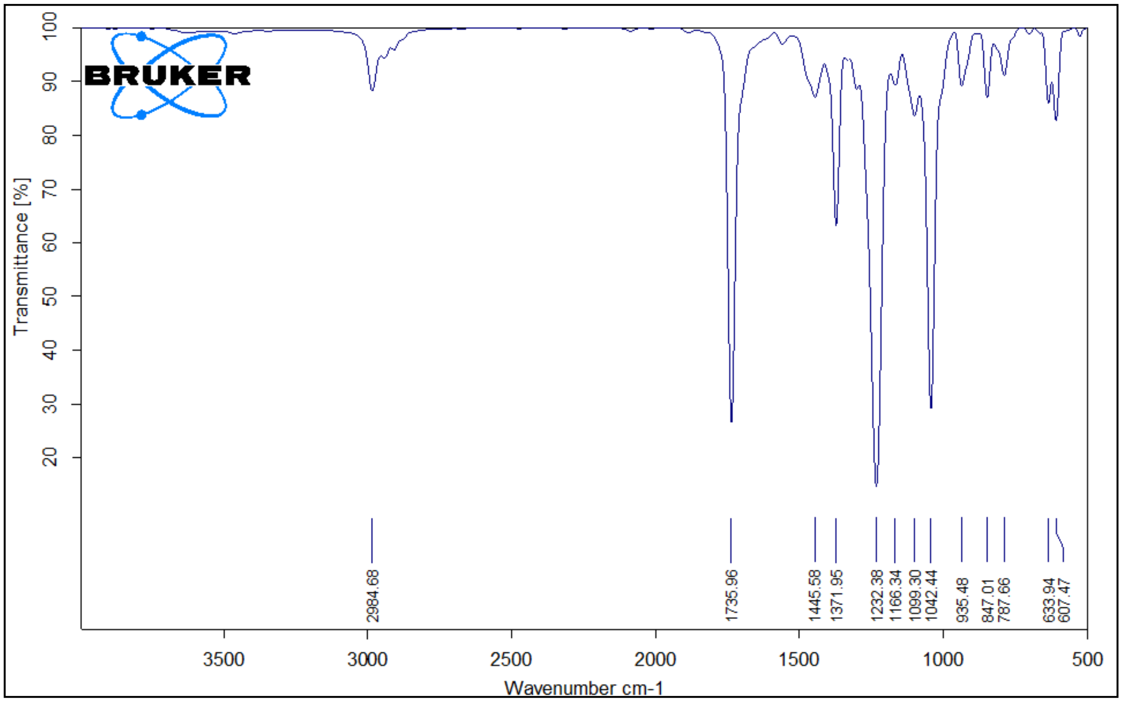

Figure S19. FTIR spectrum of 3(c)

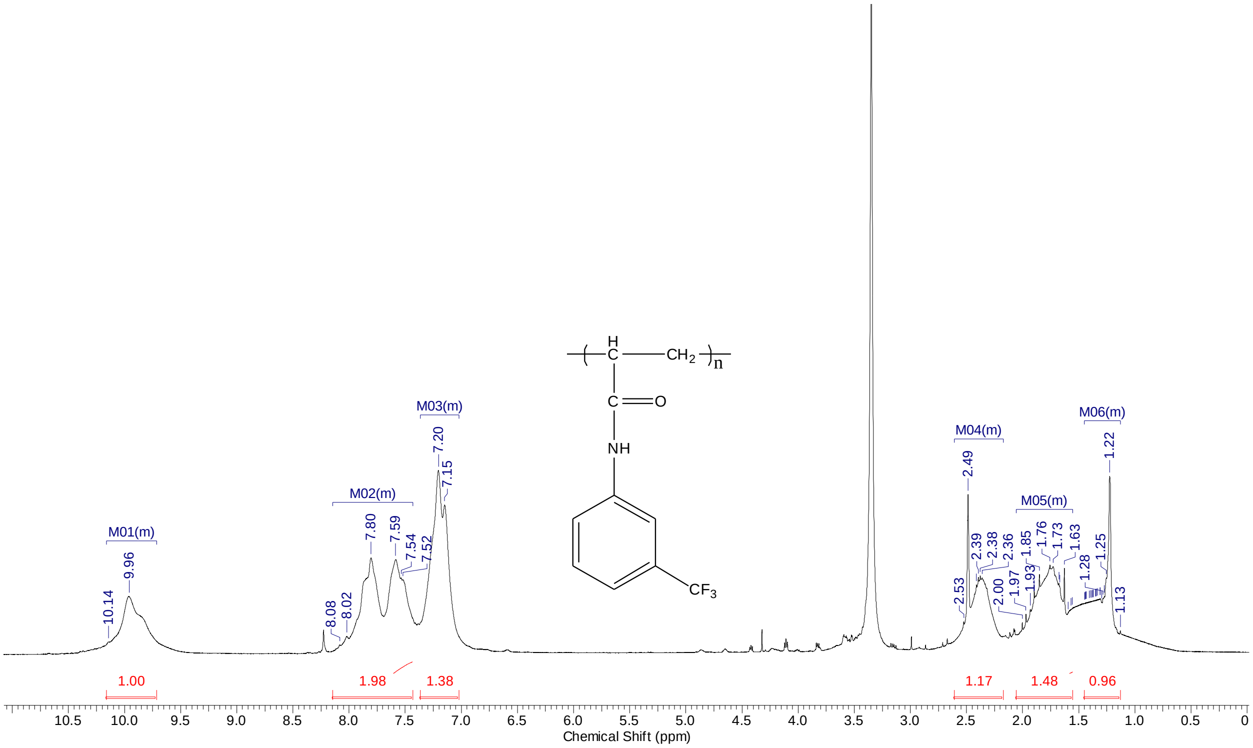

Figure S20. ^{1}H spectrum of 3(c)

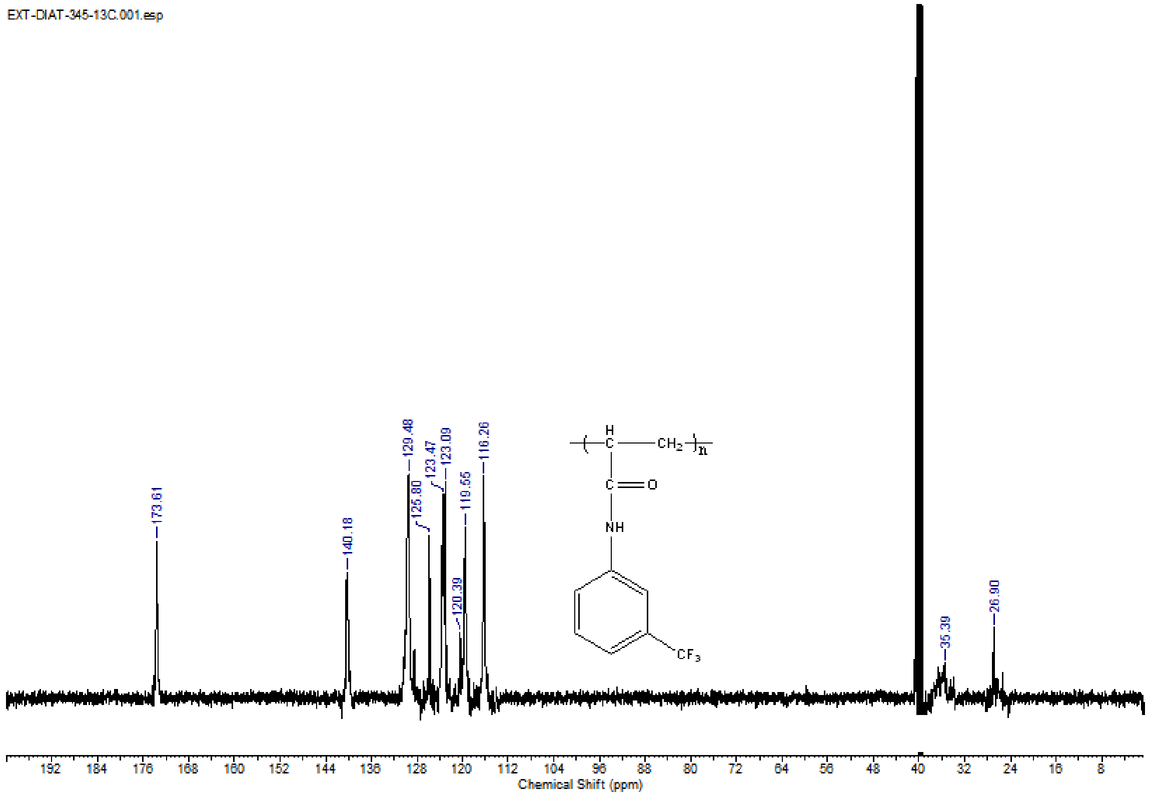

Figure S21. ^{13}C **spectrum of 3(c)**

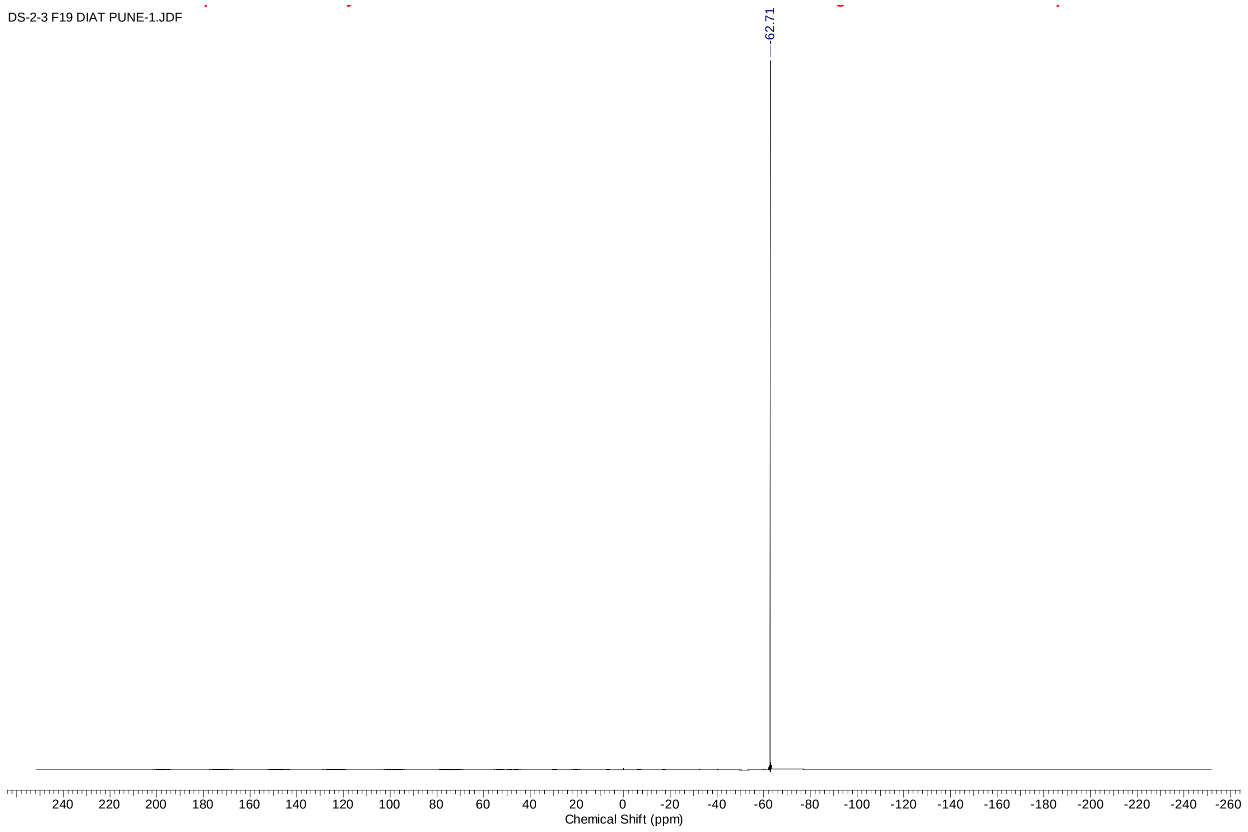

Figure S22. ^{19}F spectrum of 3(c)

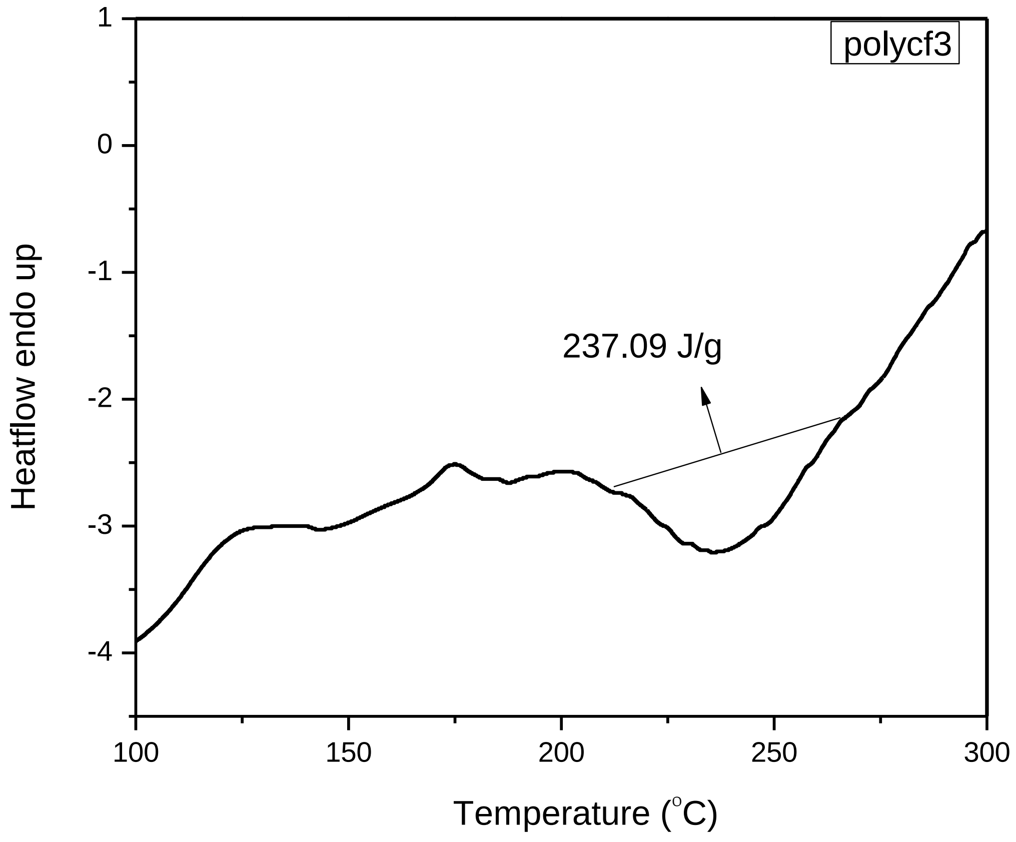

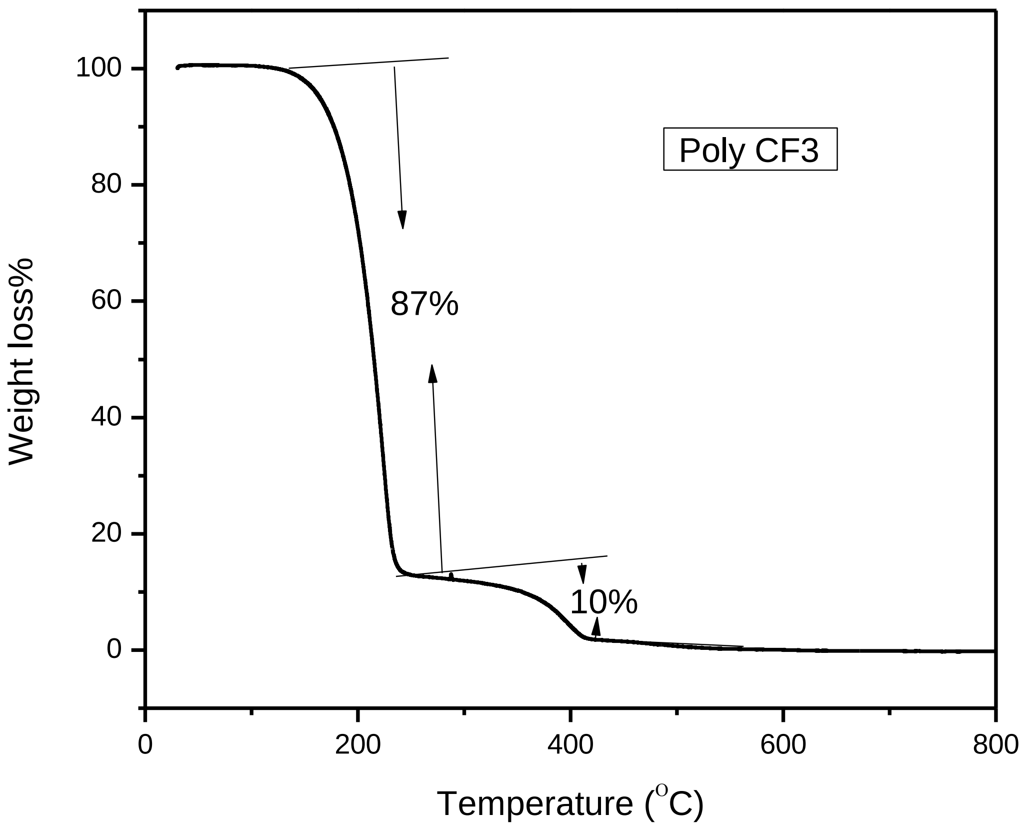

Figure S23 & S24. Thermal studies of 3(c)

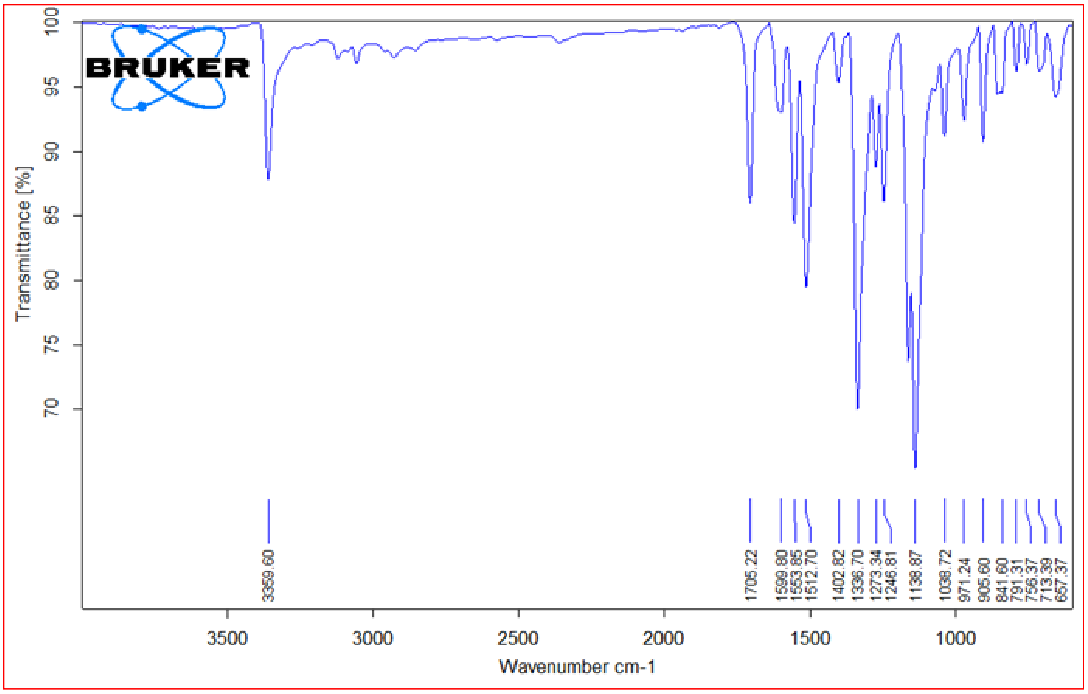

Figure S25. FTIR spectrum of 1(d)

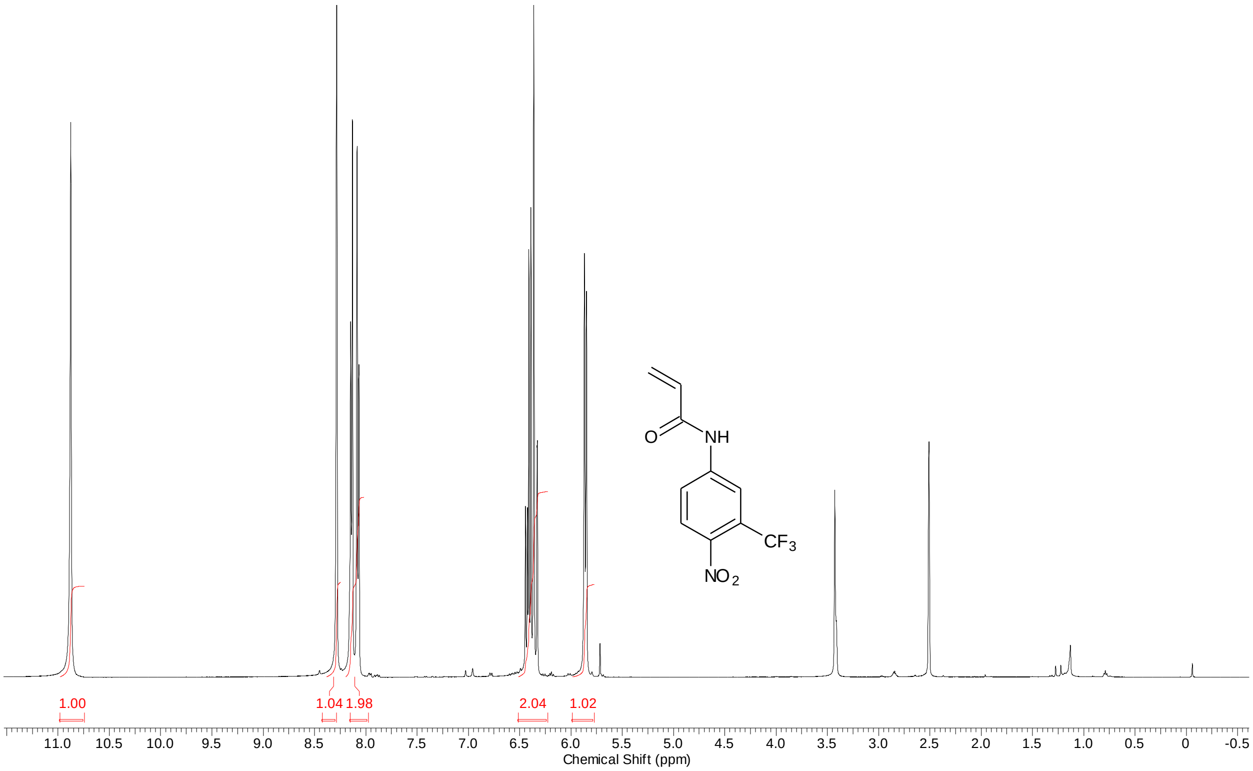

Figure S26. ^{1}H spectrum of 1(d)

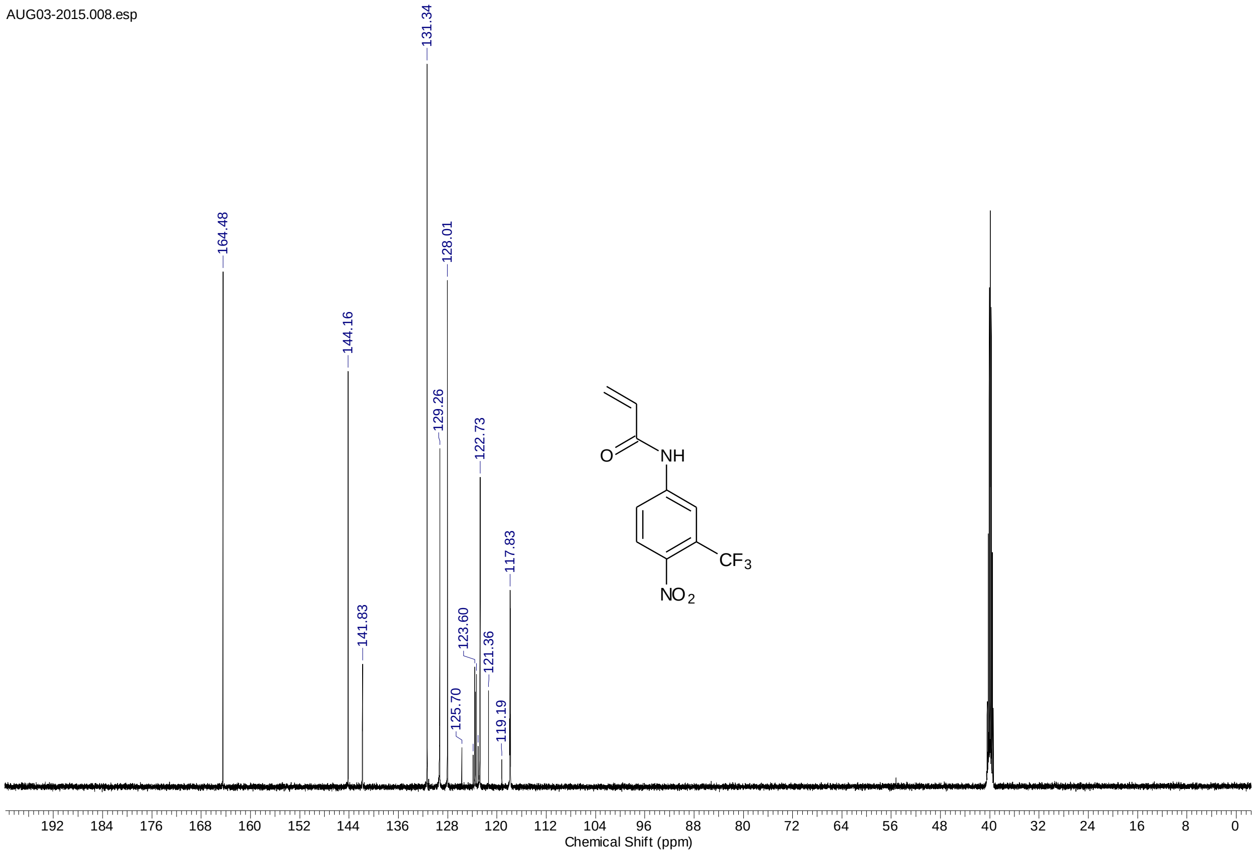

Figure S27. ^{13}C spectrum of 1(d)

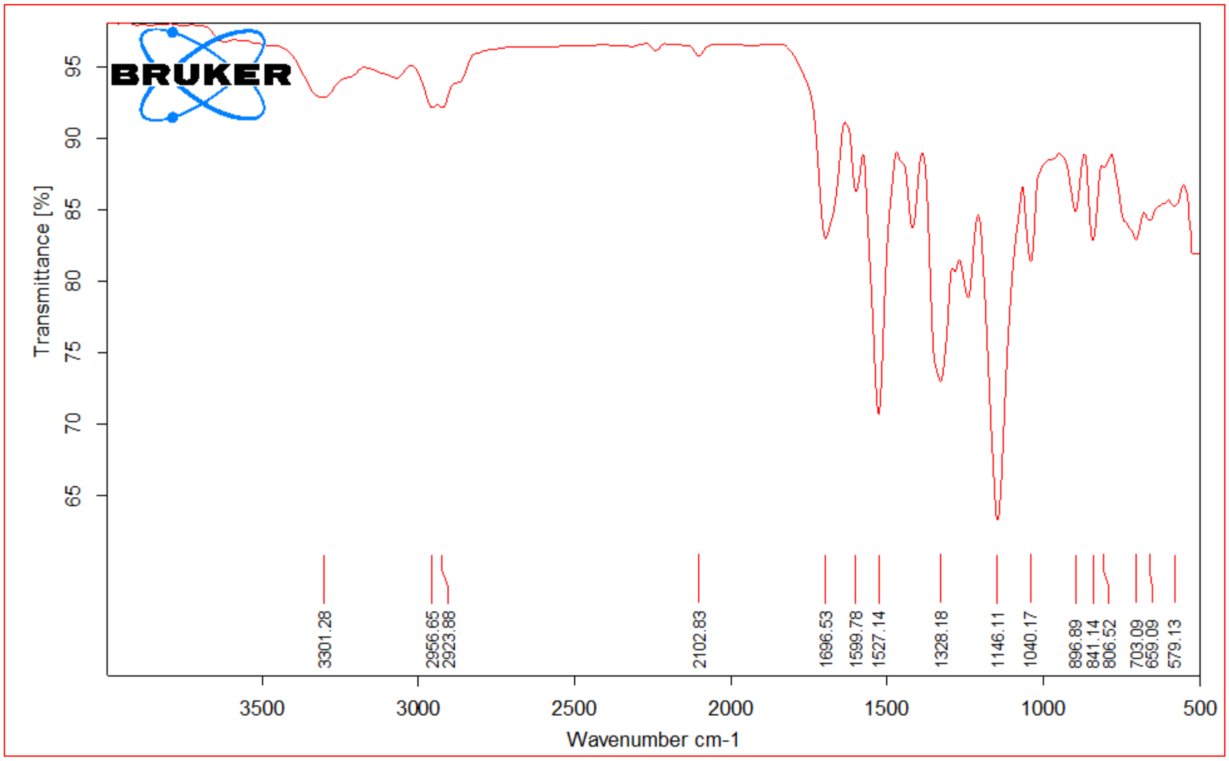

Figure S28. FTIR spectrum of 3(d)

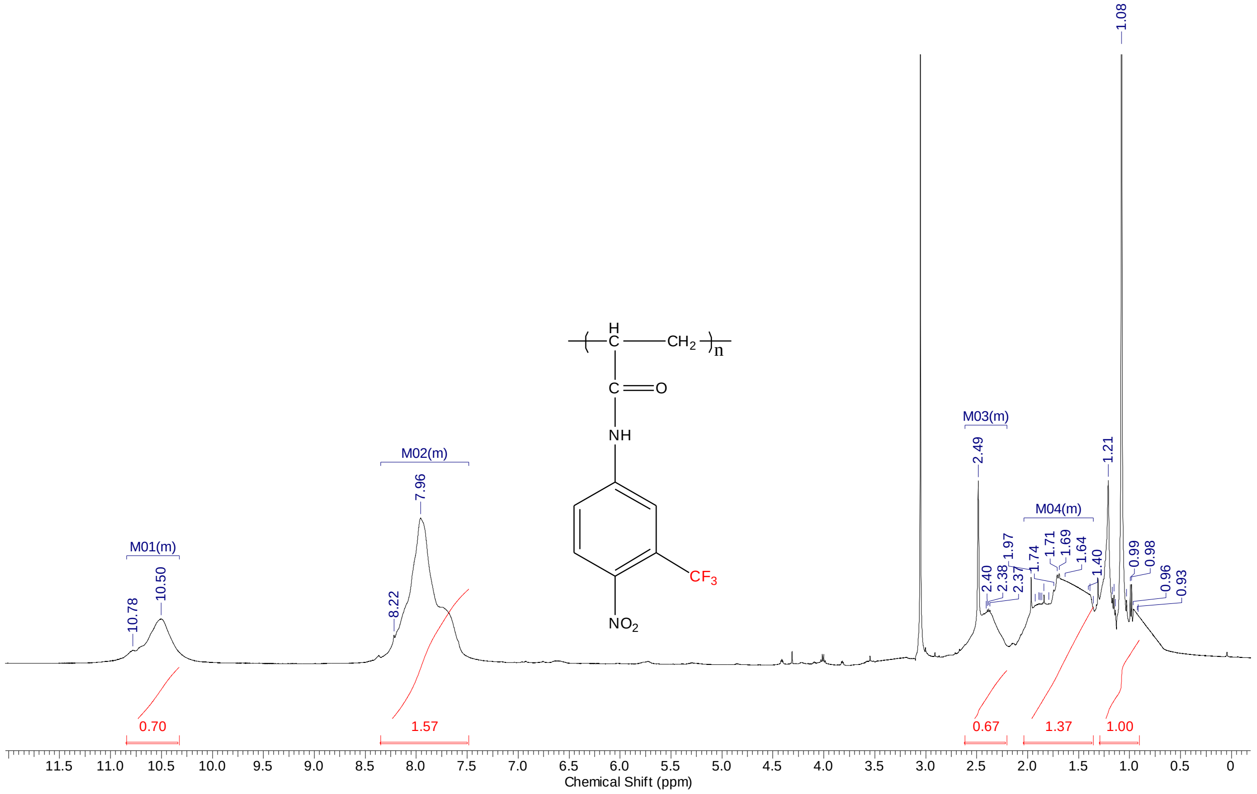

Figure S29. ^{1}H NMR spectrum of 3(d)

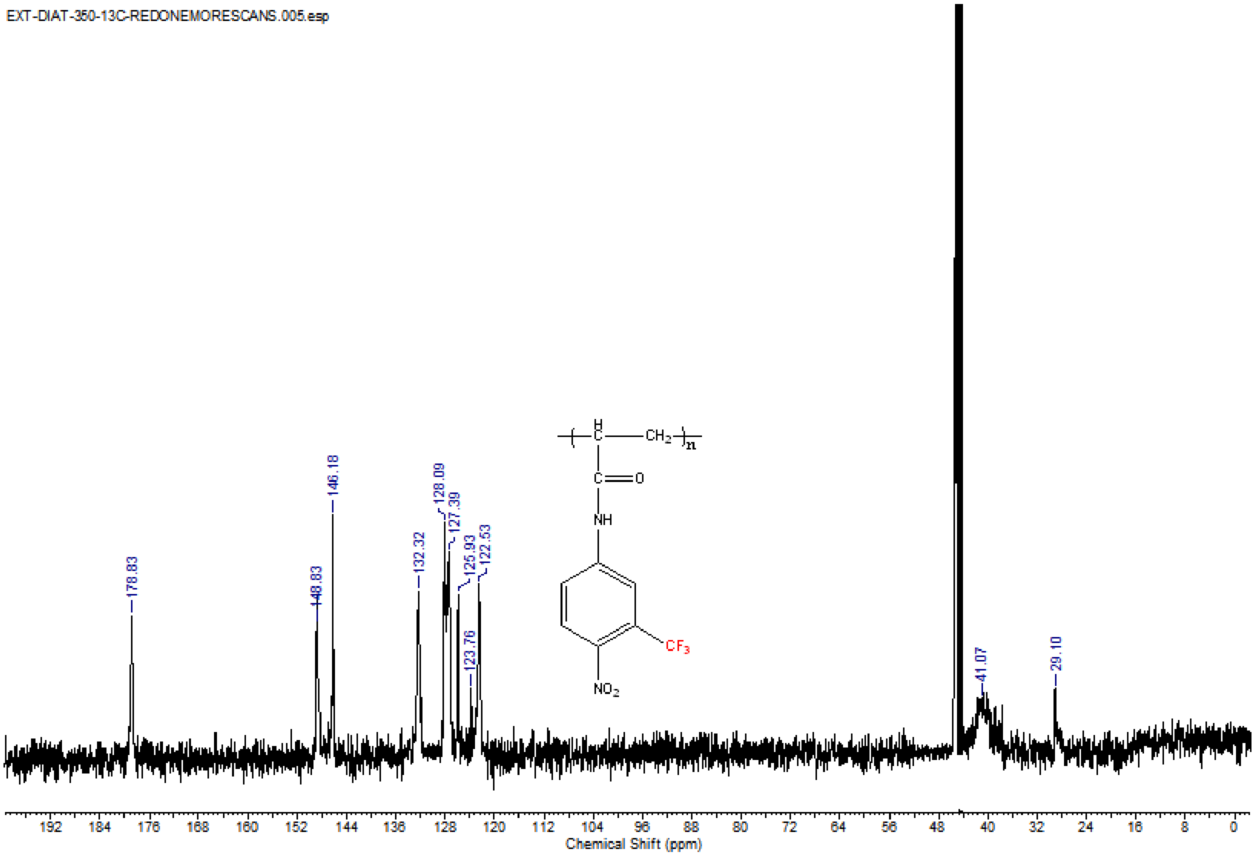

Figure S30. ^{13}**C NMR spectrum of 3(d)**

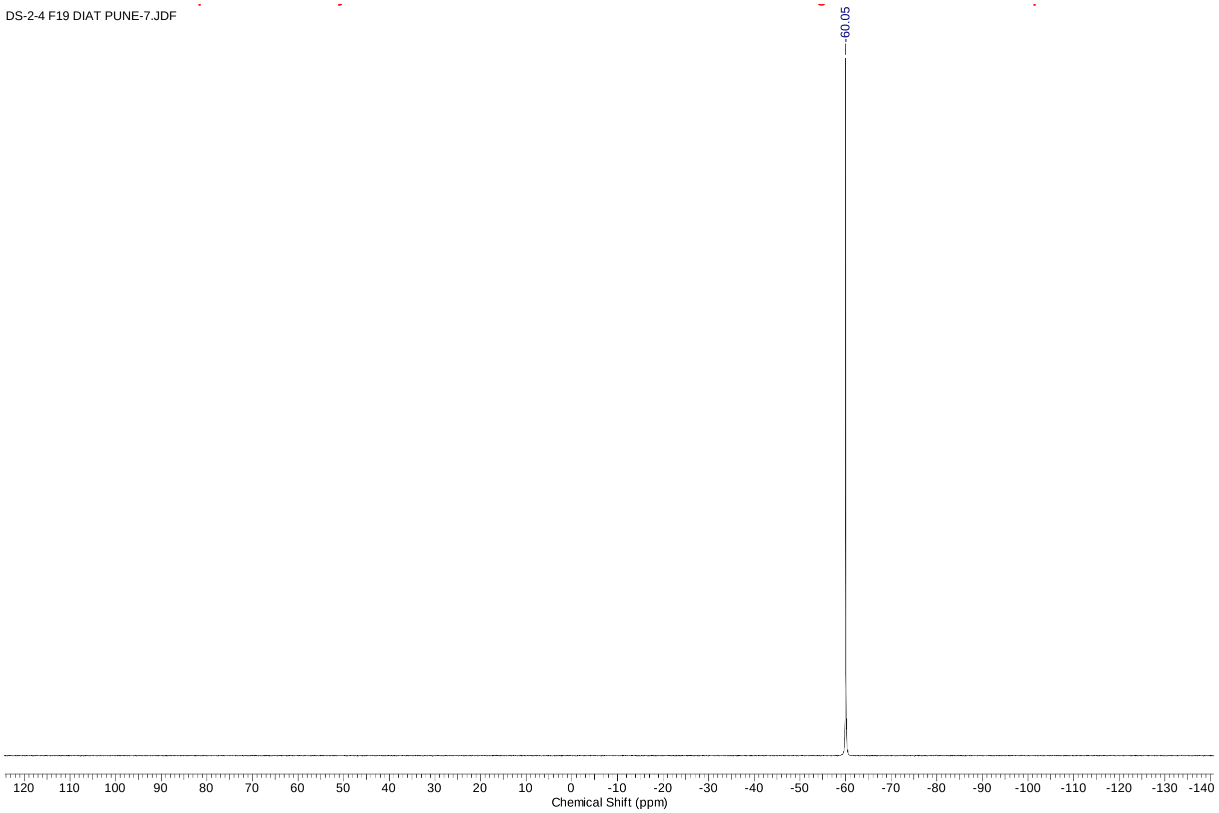

Figure S31. ^{19}F NMR spectrum of 3(d)

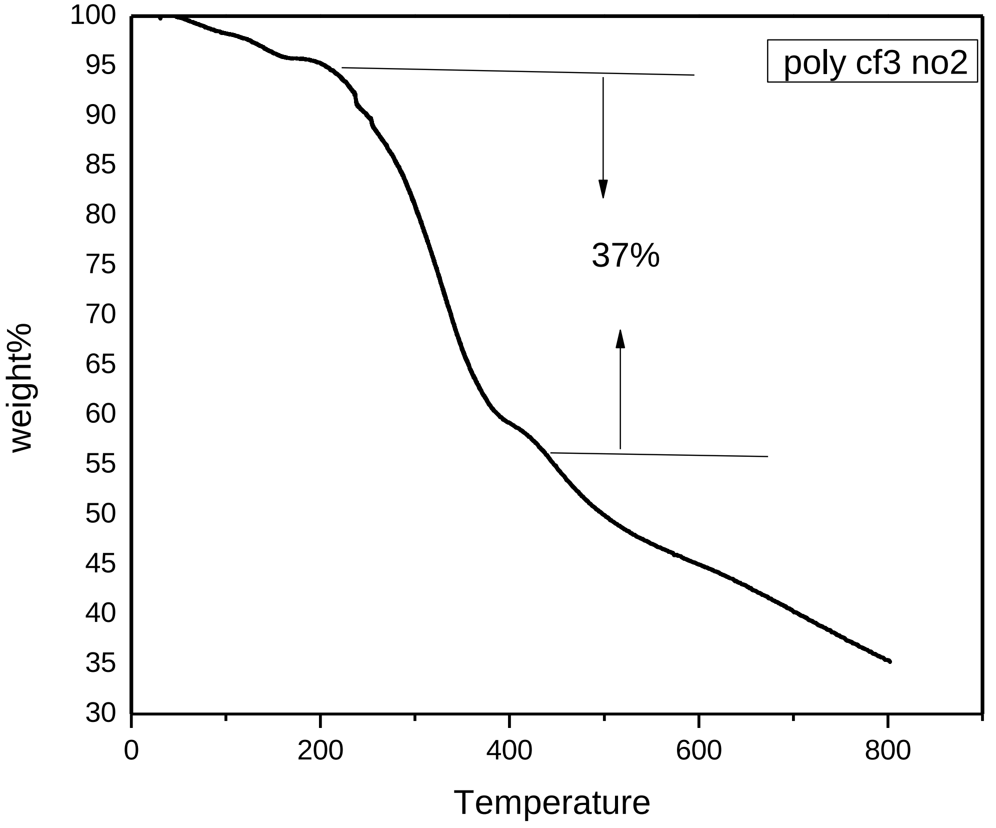
poly cf3 no2
37%
weight%
Temperature
100
95
90
85
80
75
70
65
60
55
50
45
40
35
30
0
200
400
600
800

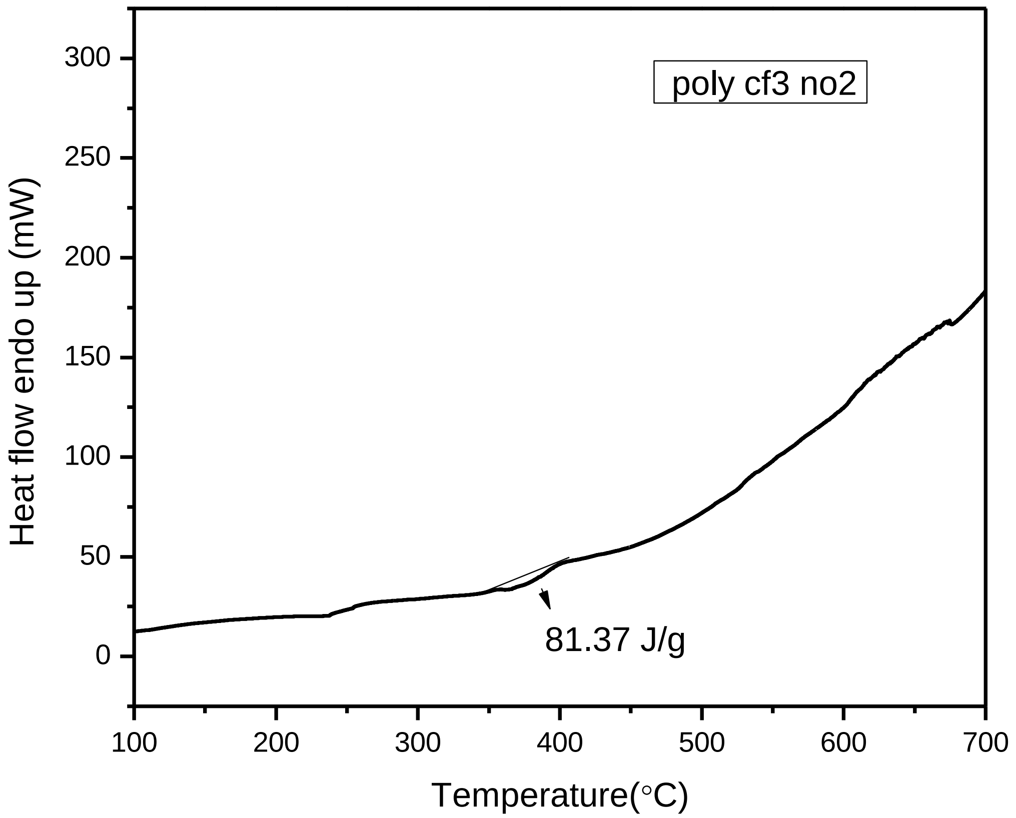
poly cf3 no2
81.37 J/g
Heat flow endo up (mW)
Temperature(°C)
300
250
200
150
100
50
0
100
200
300
400
500
600
700

Figure S31& S32. Thermal studies of 3(d)

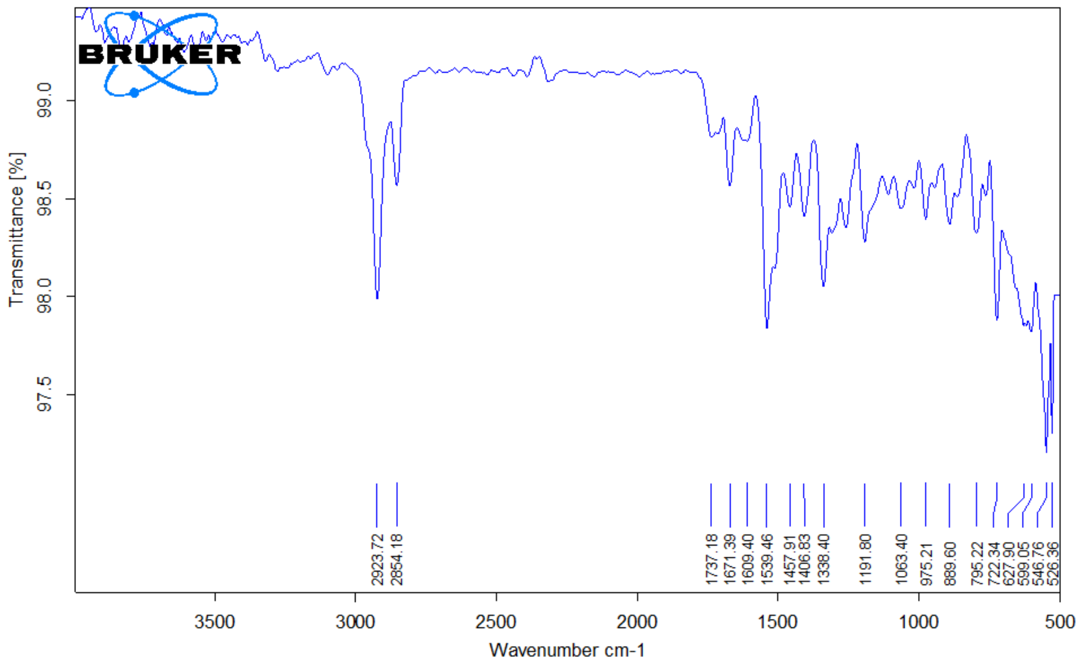

Figure S33. FTIR spectrum of 1(e)

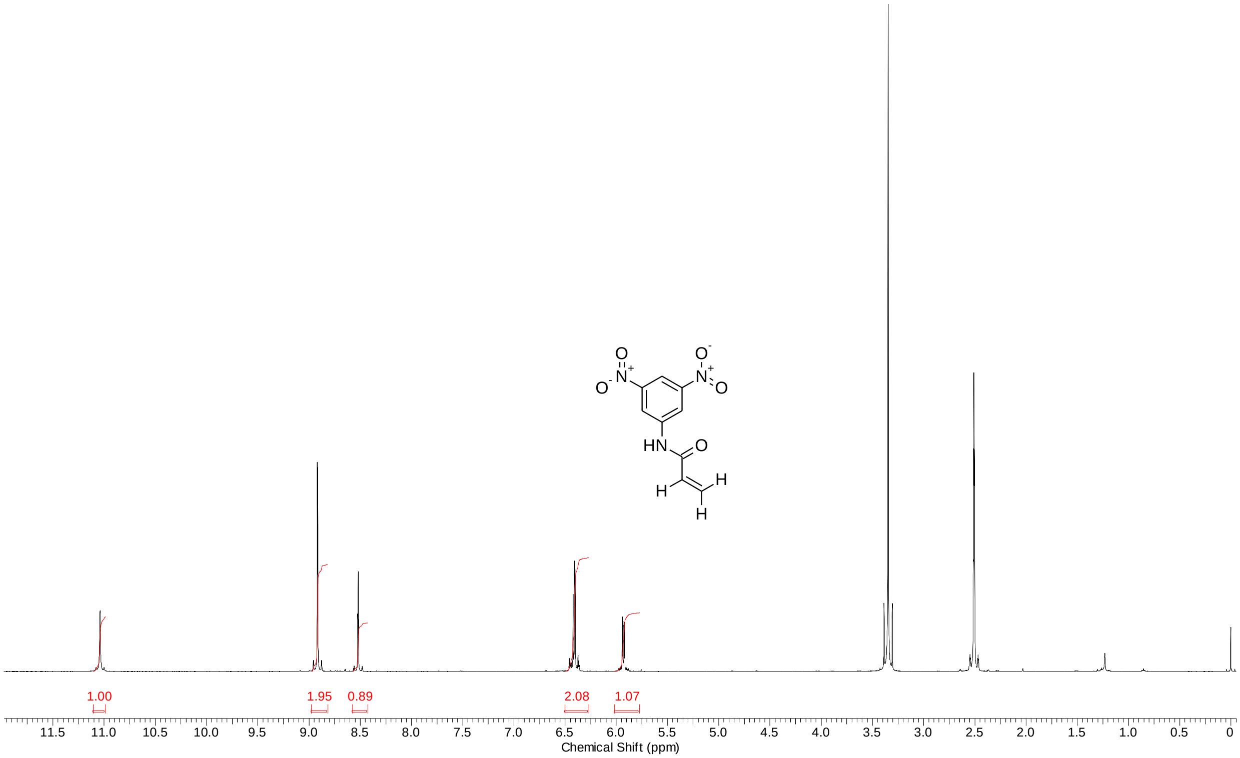

Figure S34. ^{1}H NMR spectrum of 1(e)

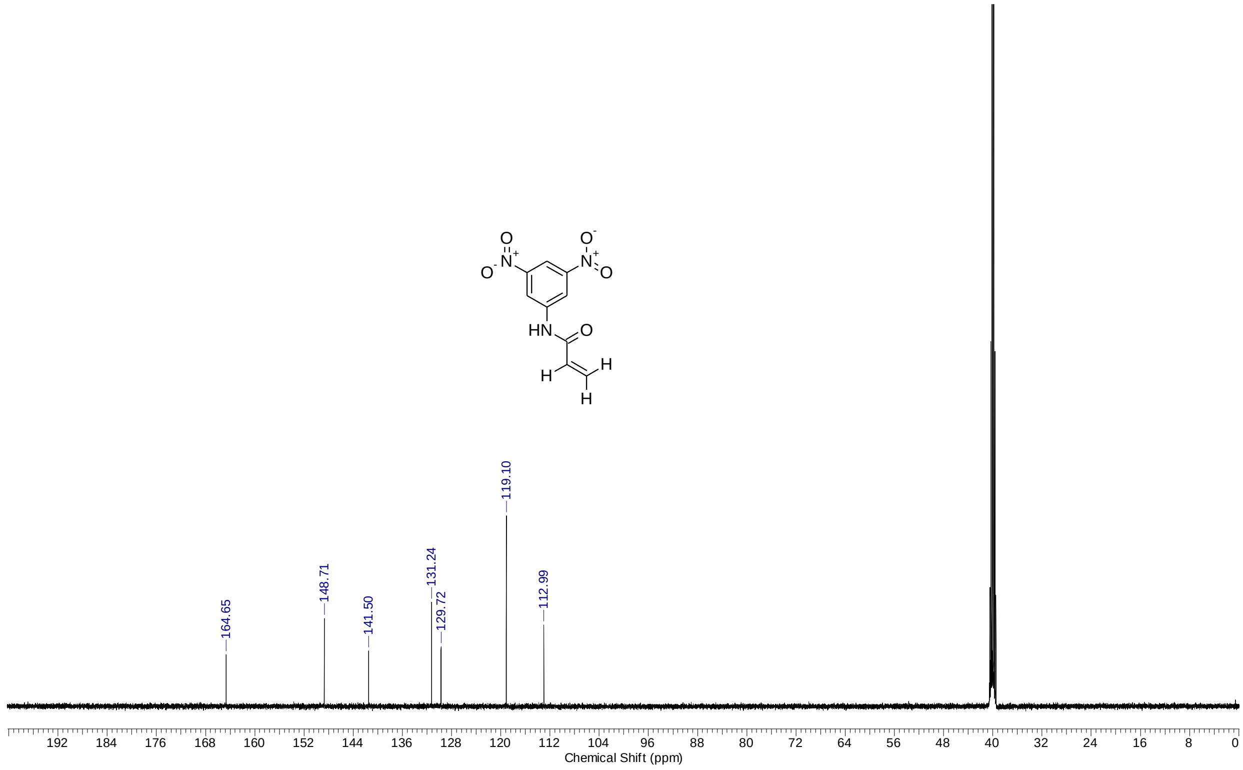

Figure S35. ^{13}C NMR spectrum of 1(e)

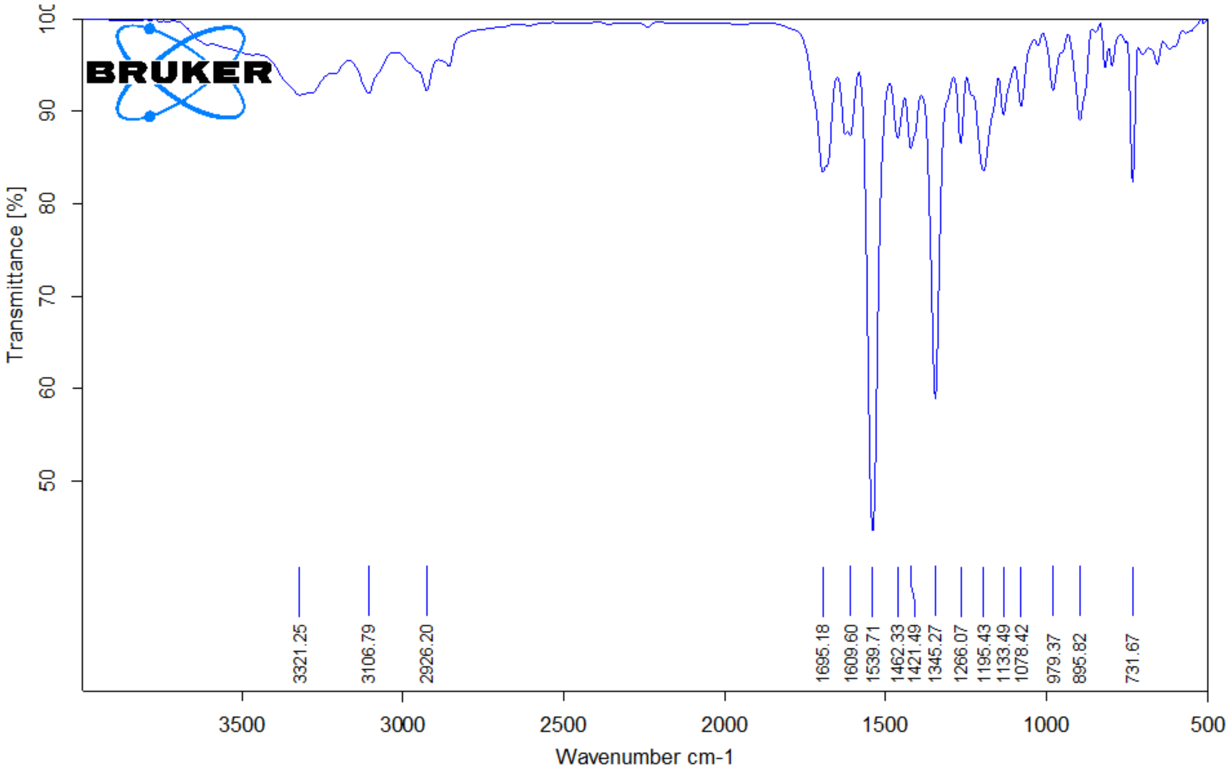

Figure S36. FTIR spectrum of 3(e)

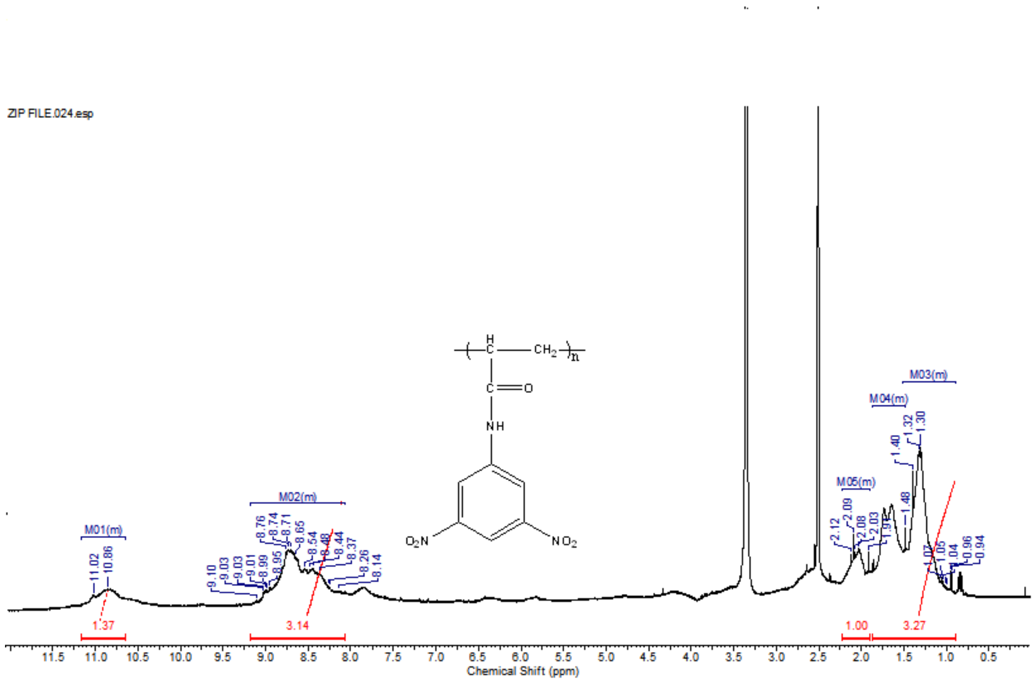

Figure S37. ^{1}H NMR spectrum of 3(e)

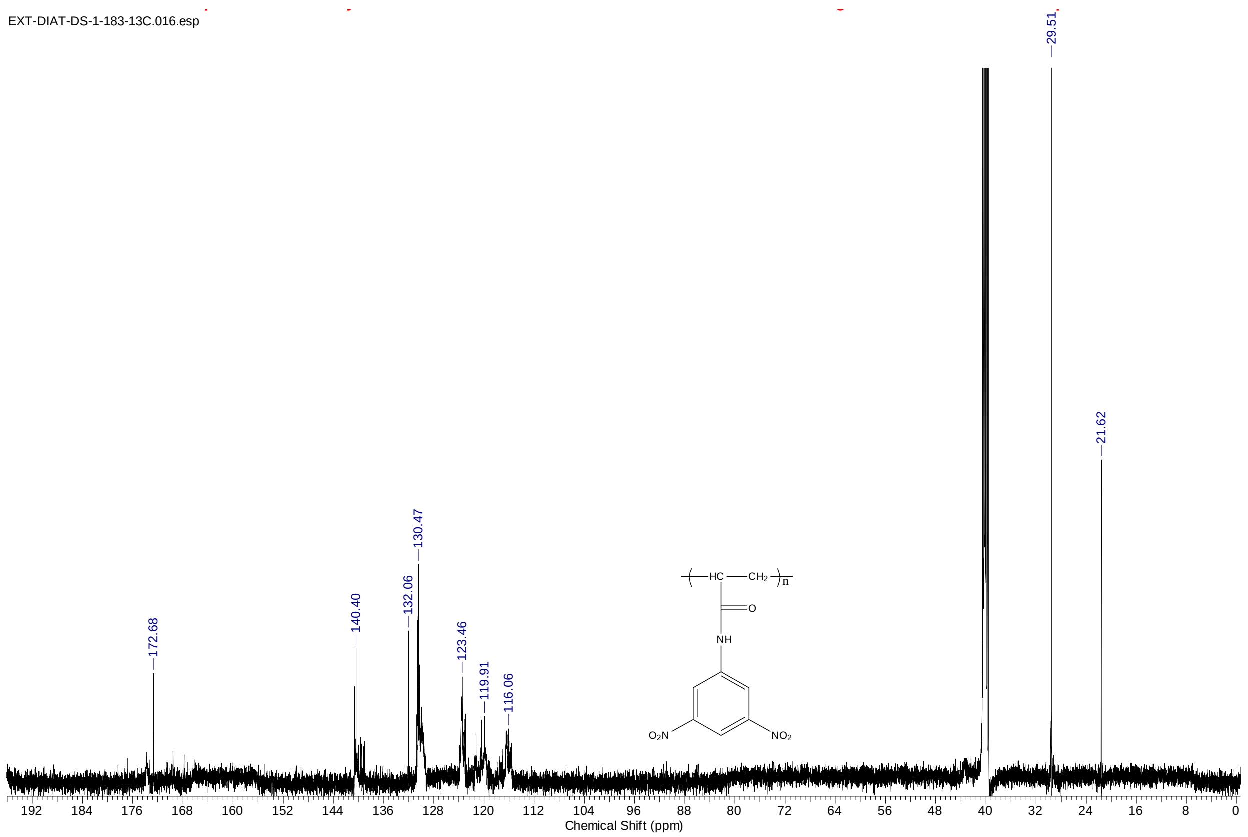

Figure S38. ^{13}C NMR spectrum of 3(e)

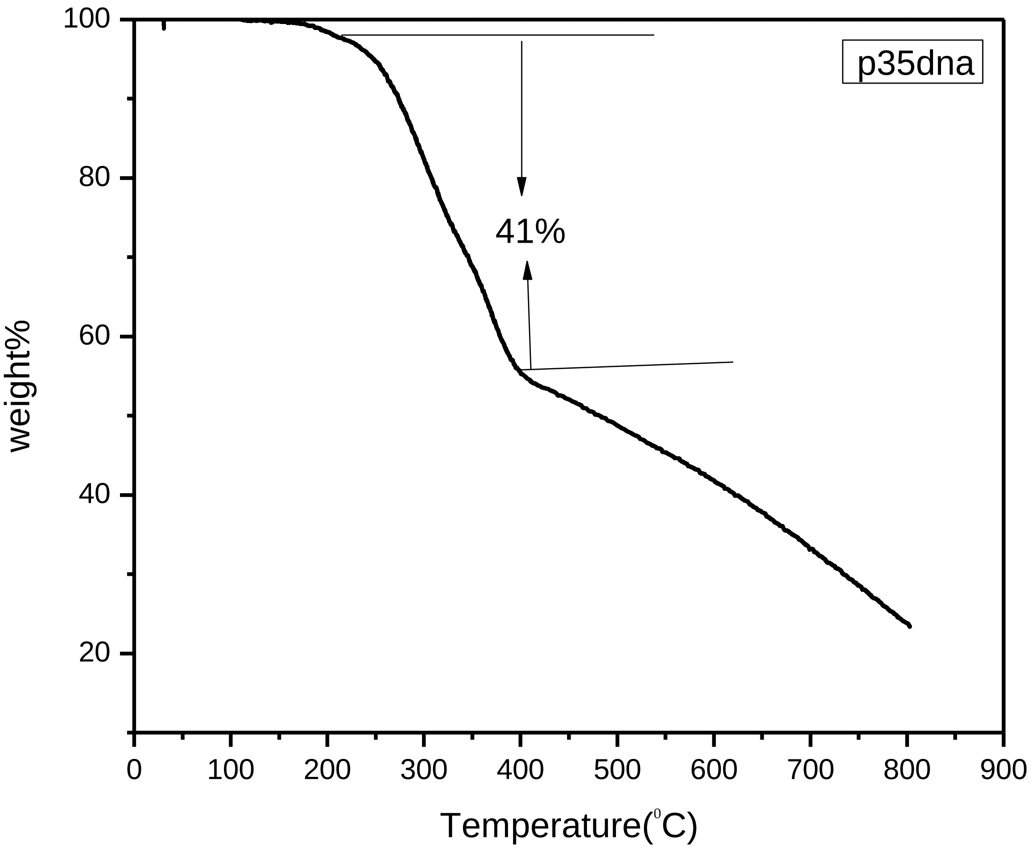

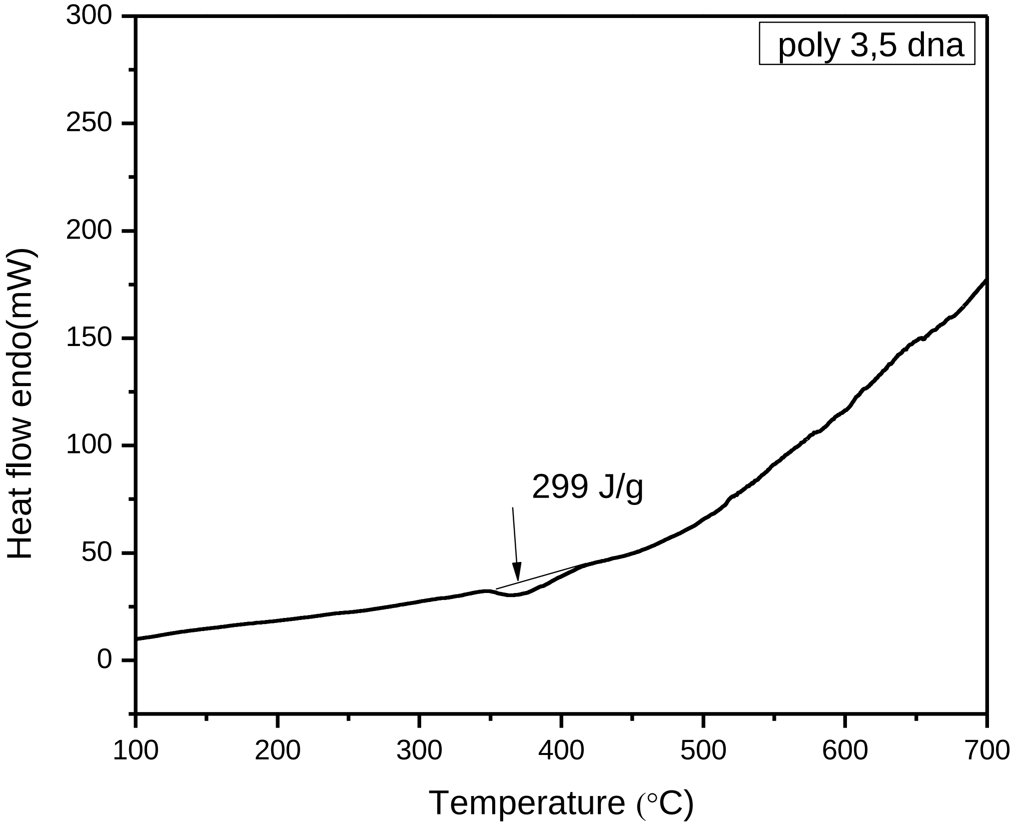

Figure S39& S40. Thermal studies of 3(e)

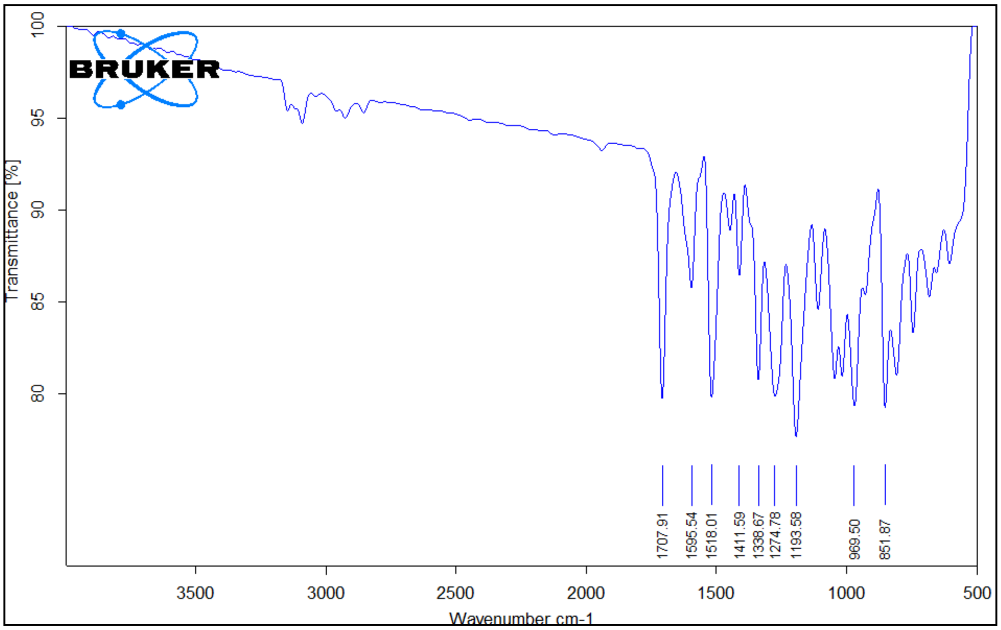

Figure S41. FTIR spectrum of 2(a)

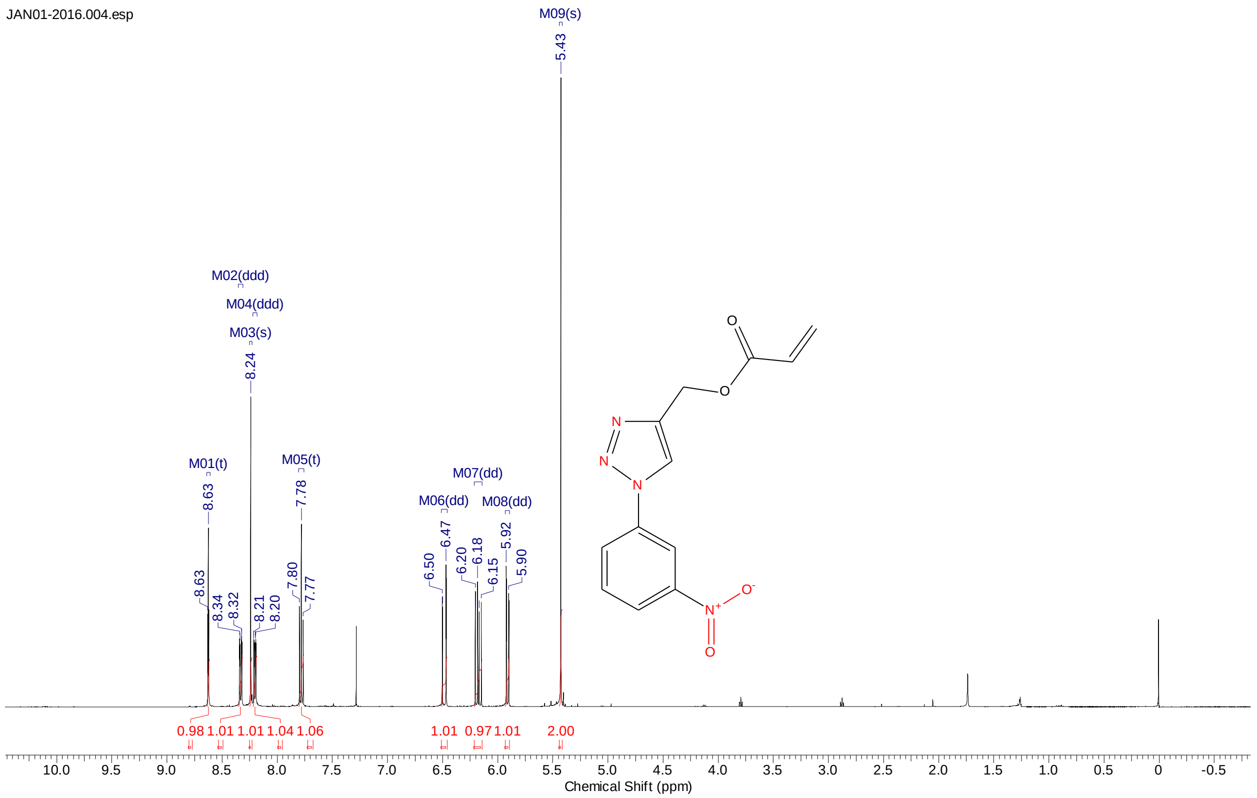

Figure S42. ^{1}H **spectrum of 2(a)**

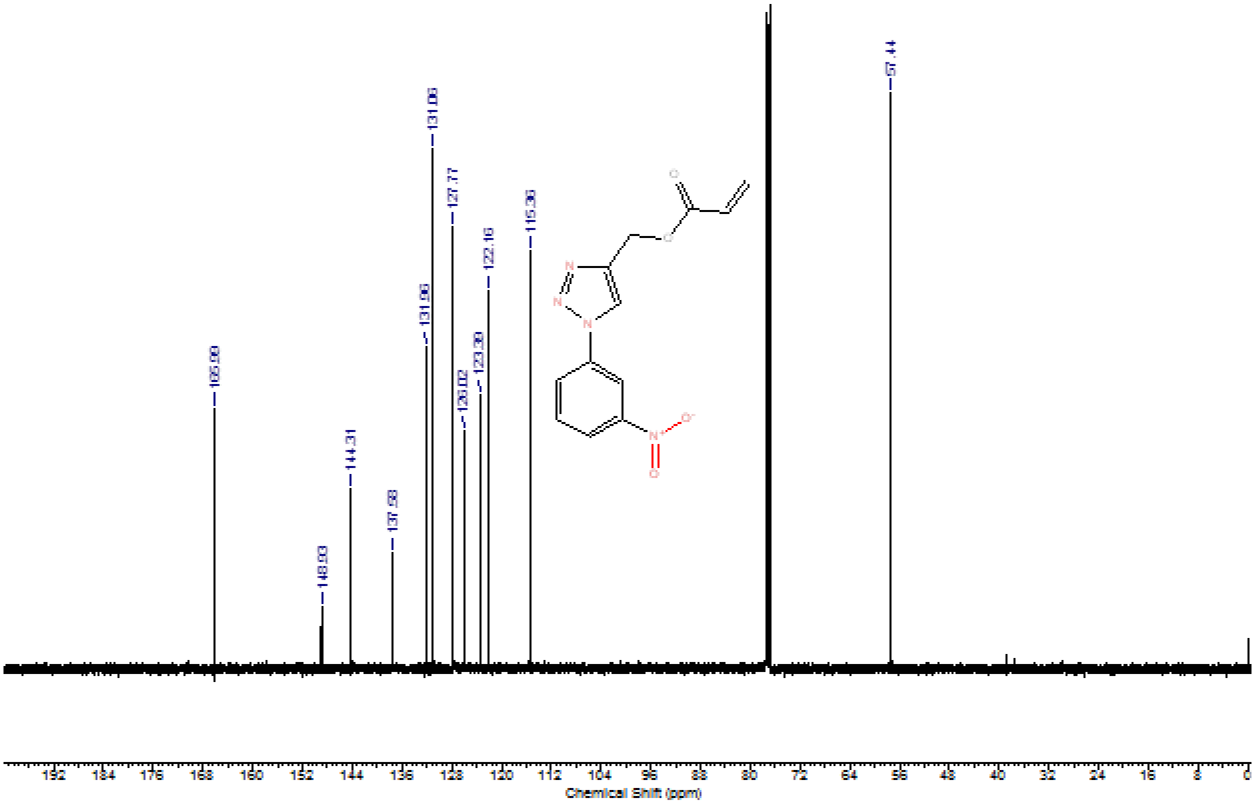

Figure S43. [13] C spectrum of 2(a)

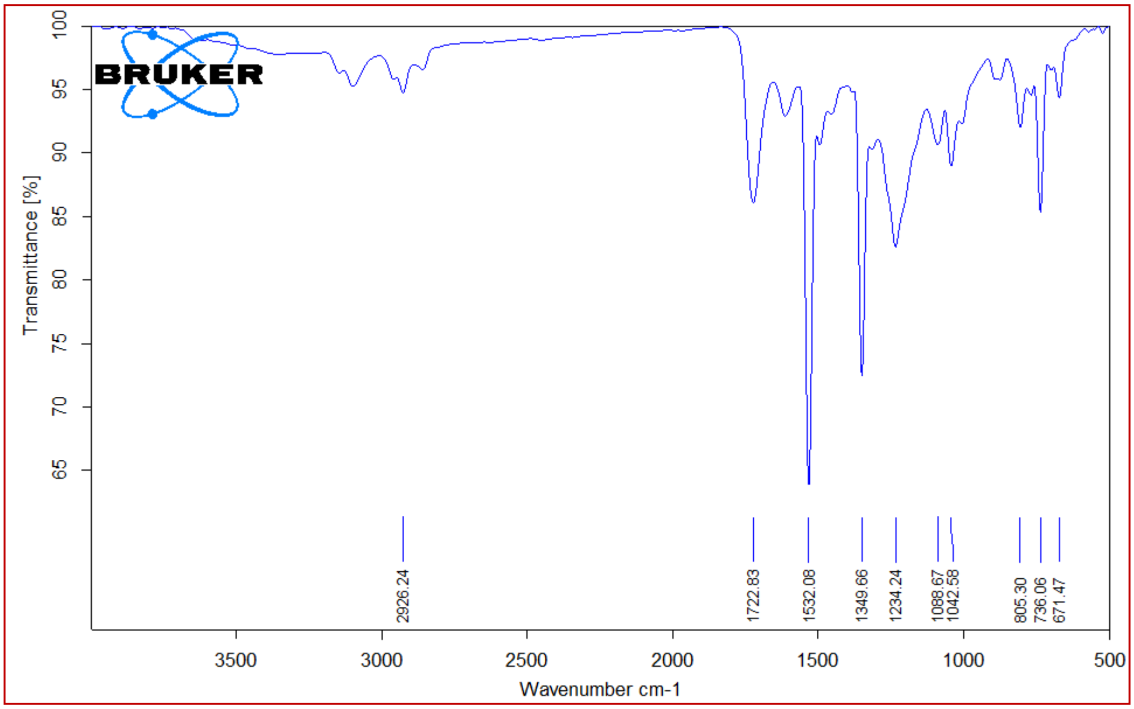

Figure S44. FTIR spectrum of 3(f)

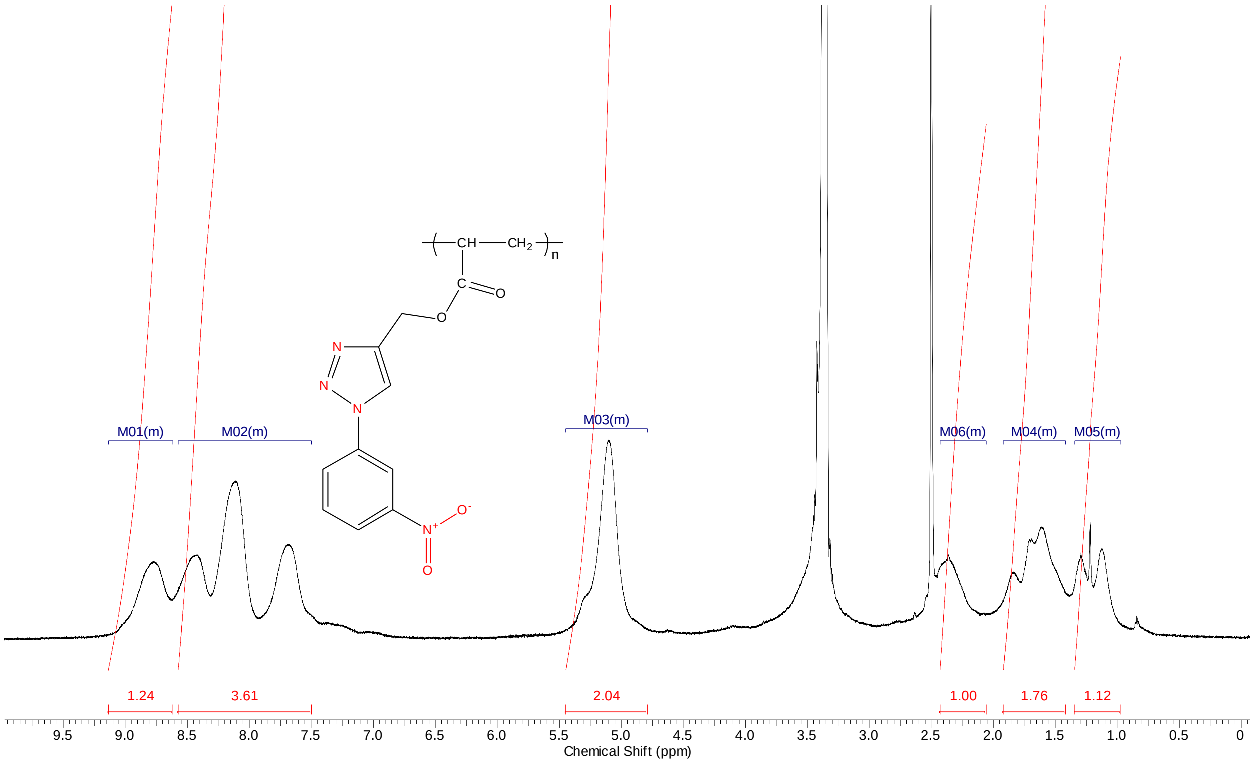

Figure S45. ^{1}H NMR spectrum of 3(f)

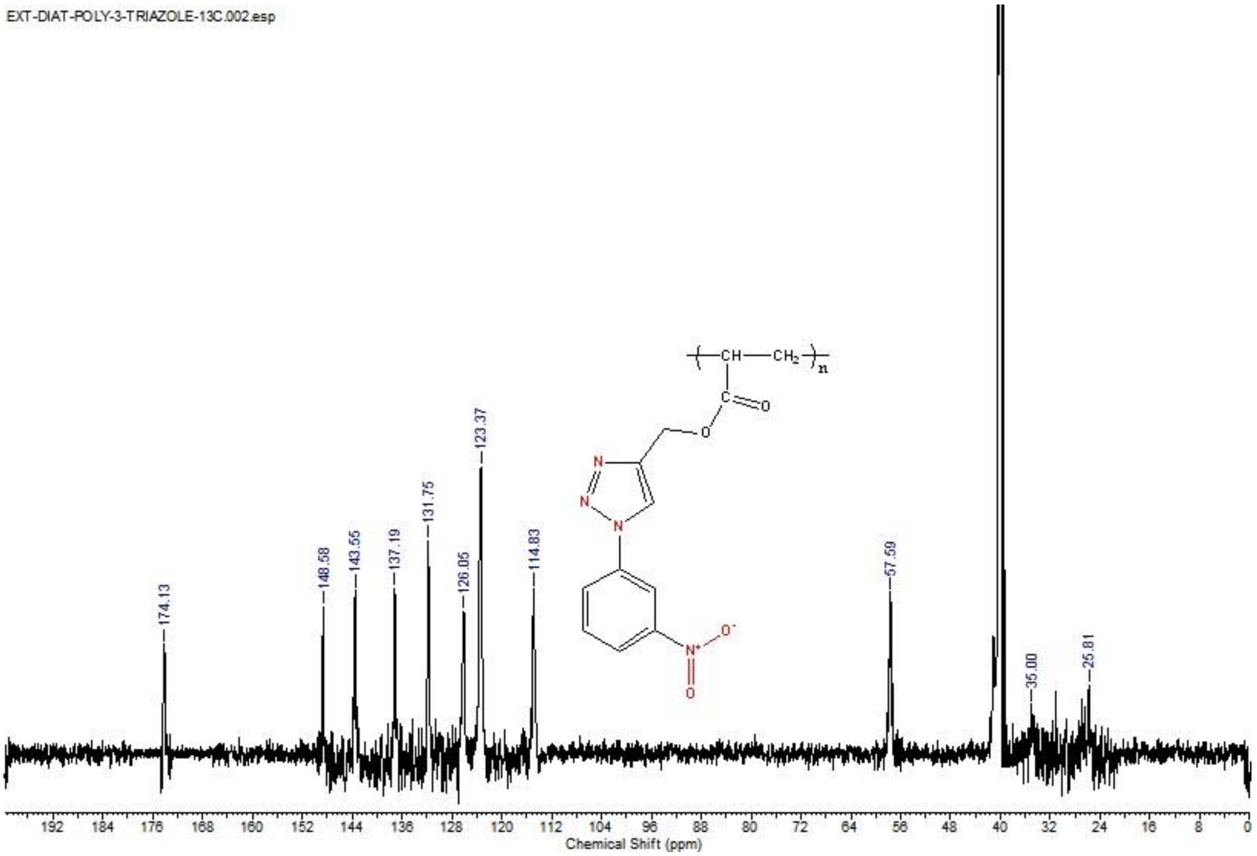

Figure S46. [13] C NMR spectrum of 3(f)

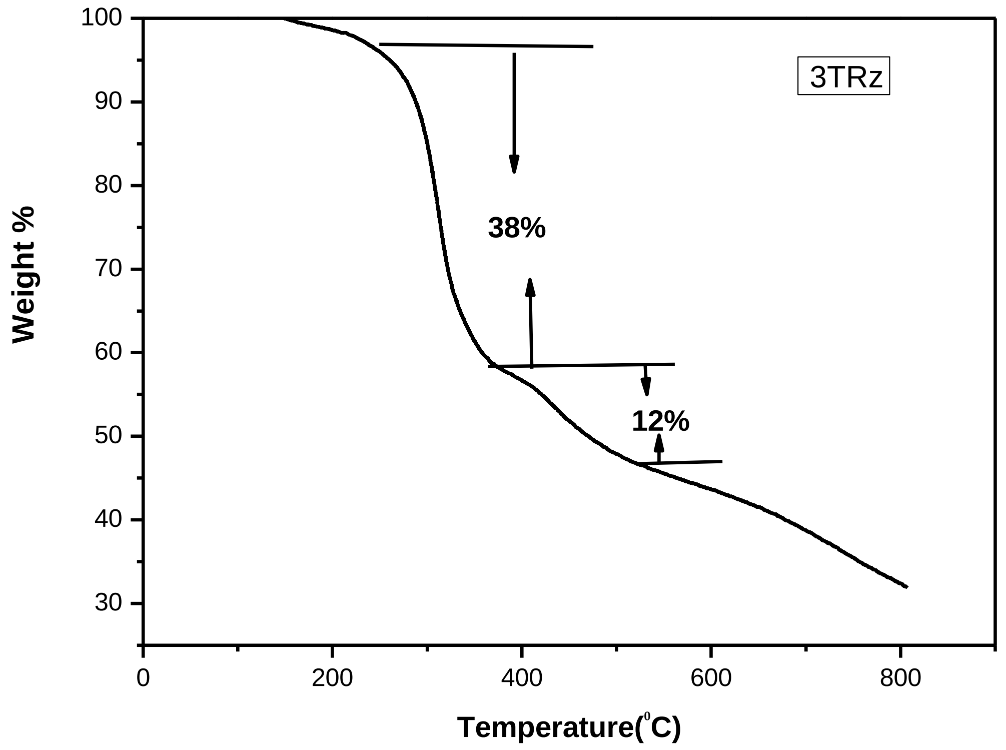

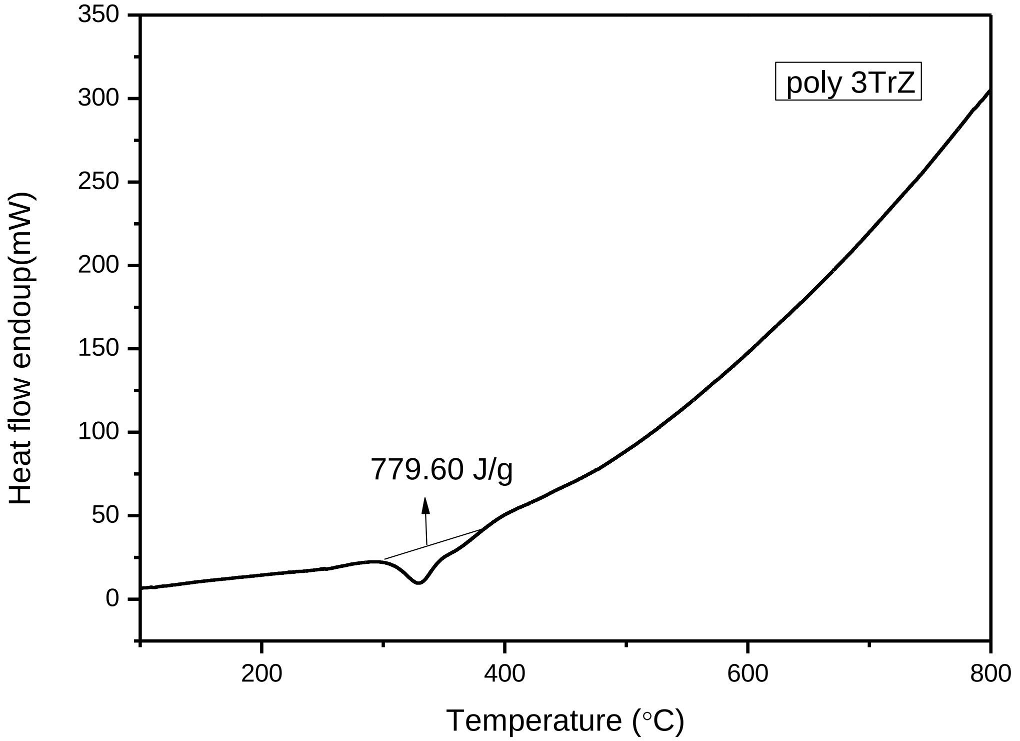

Figure S47& S48. Thermal studies of 3(f)

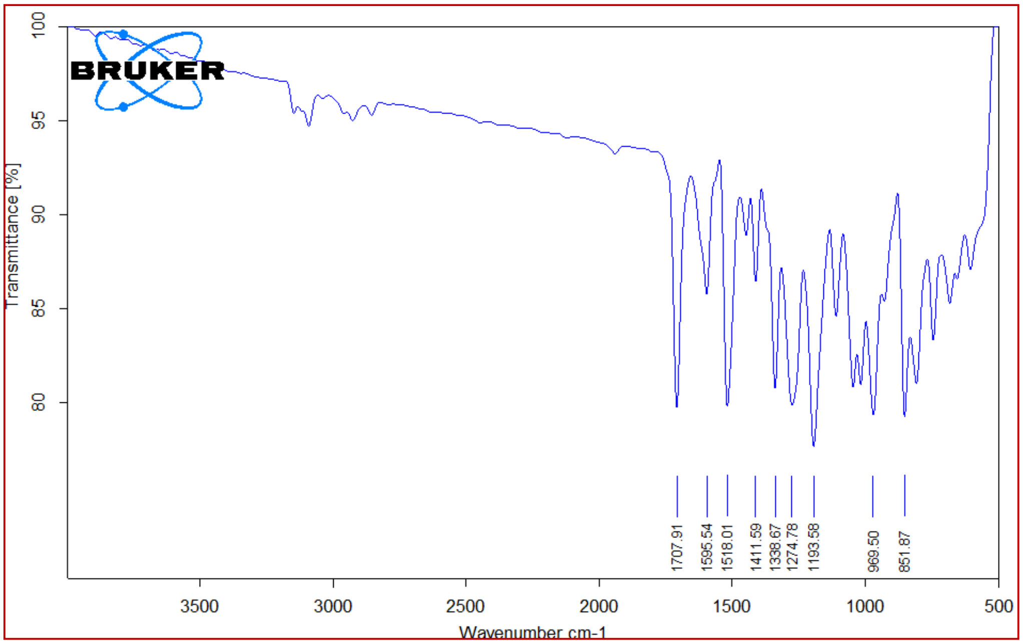

Figure S49. FTIR spectrum of 2(b)

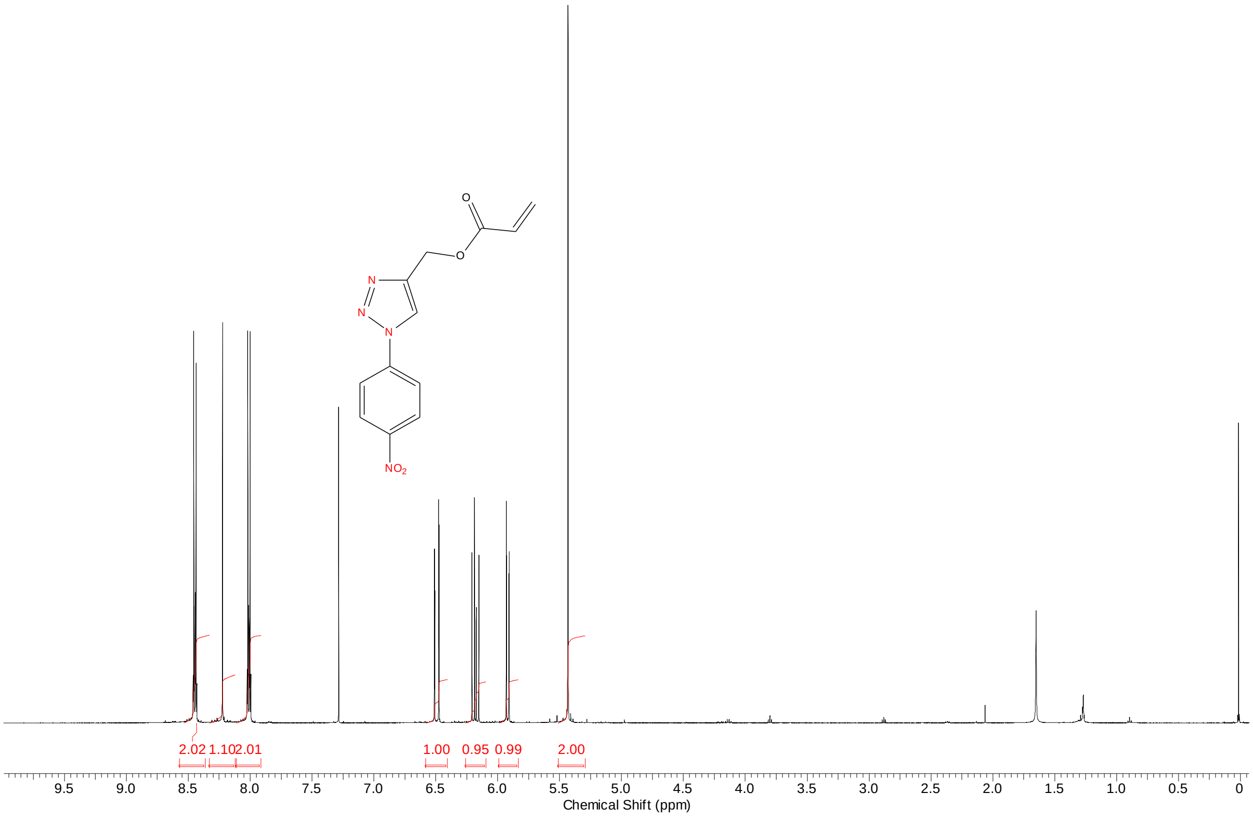

Figure S50. ^{1}H NMR spectrum of 2(b)

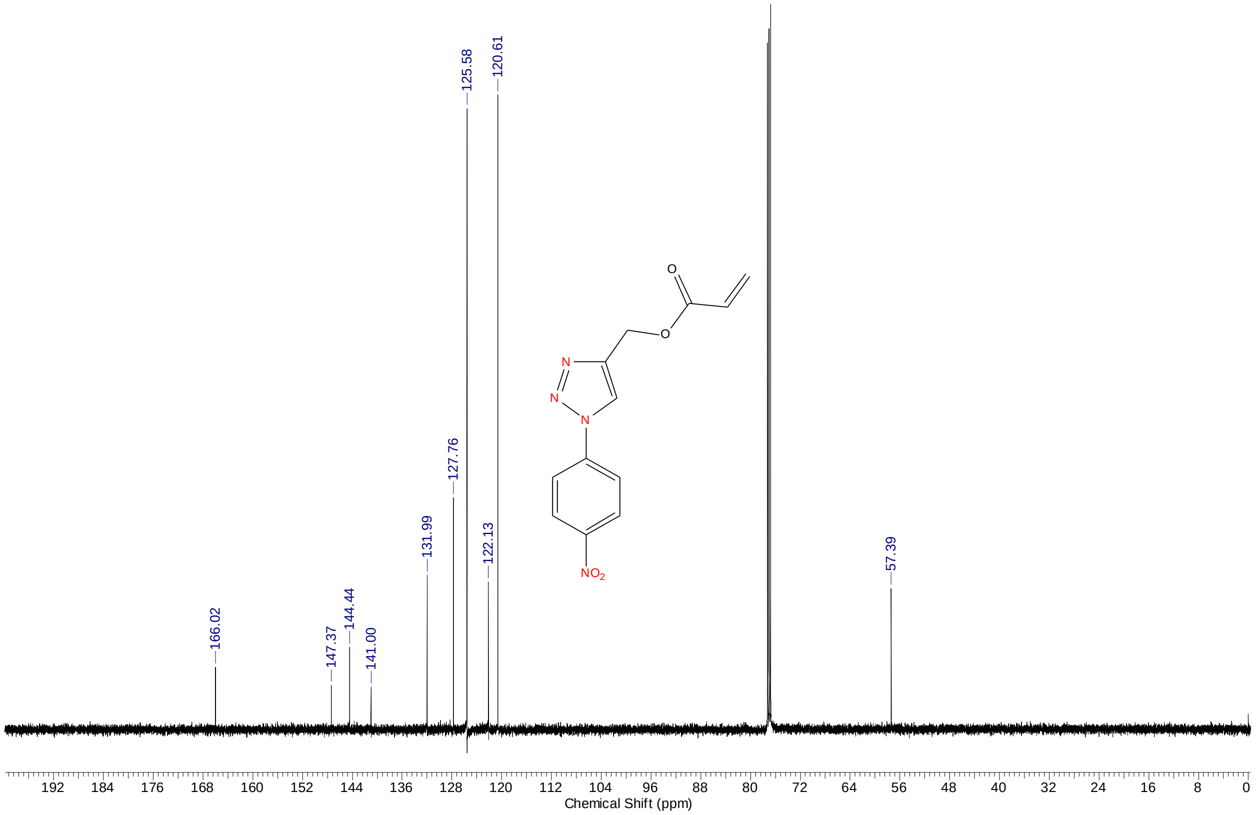

Figure S51. ^{13}C NMR spectrum of 2(b)

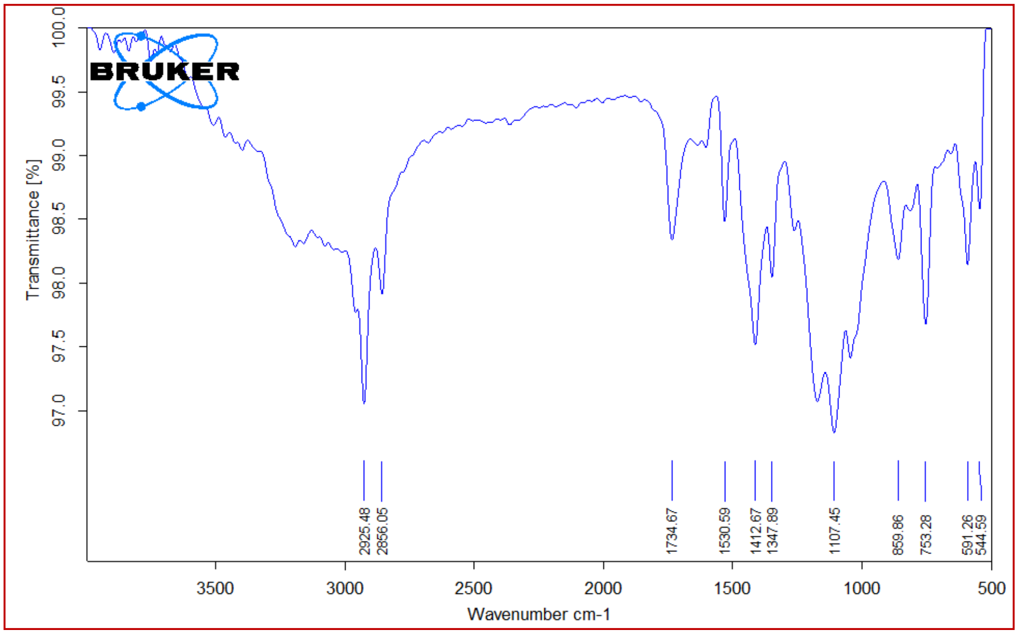

Figure S52. FTIR spectrum of 3(g)

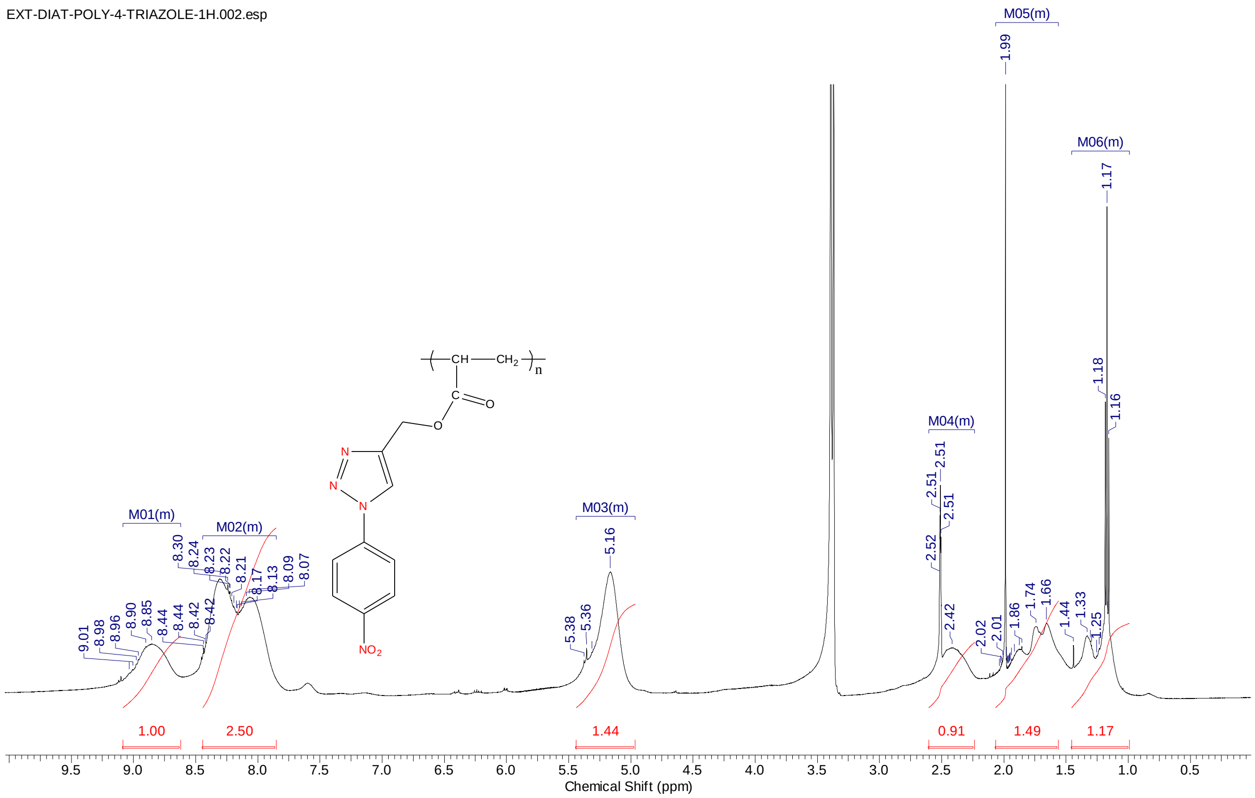

Figure S53. ^{1}H NMR spectrum of 3(g)

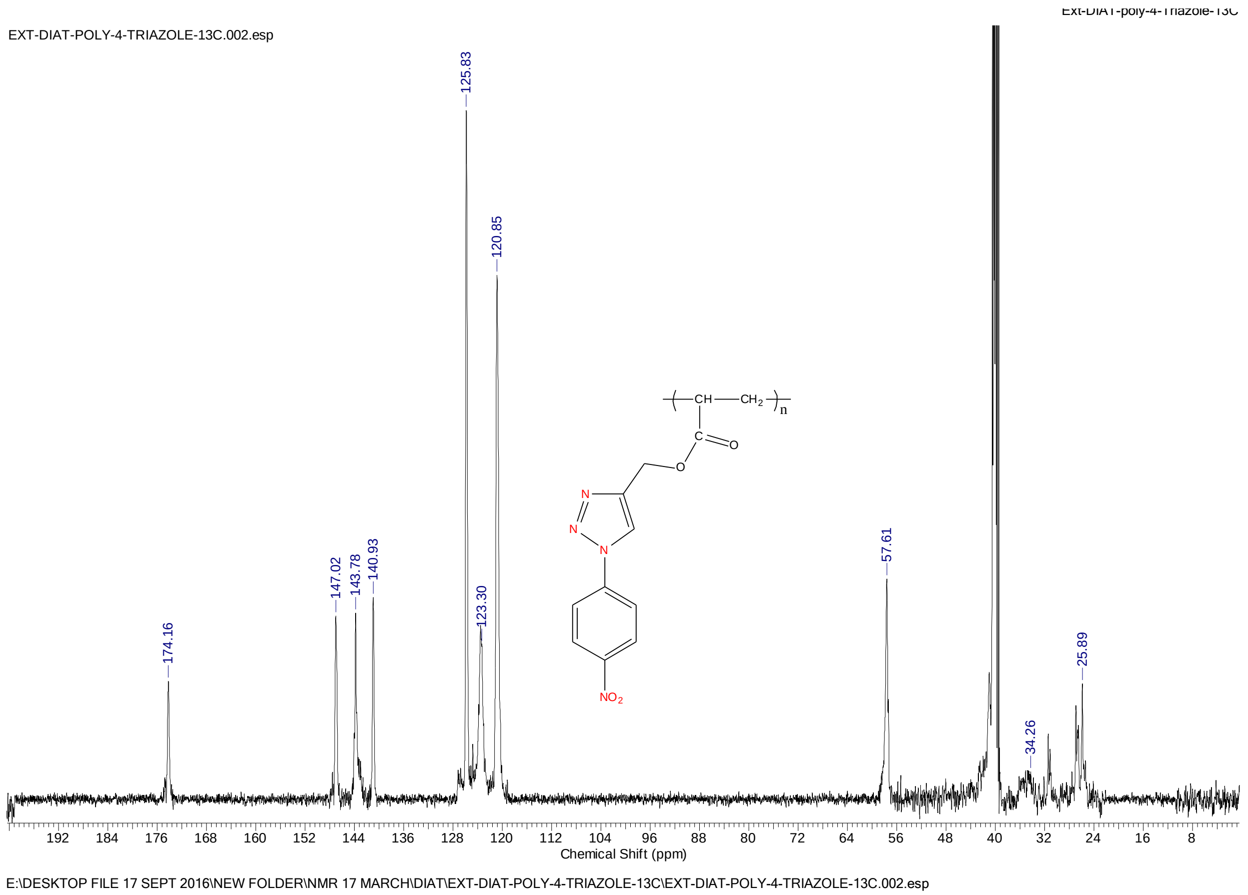

Figure S54. ^{13}C NMR spectrum of 3(g)

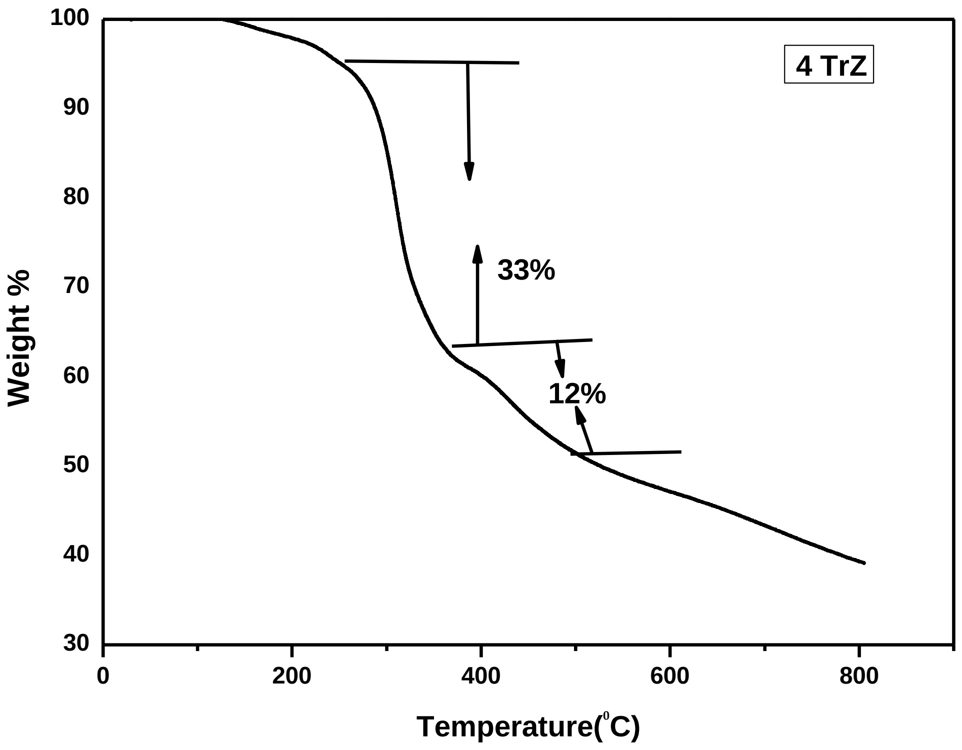
4 TrZ
33%
12%
Weight %
Temperature(⁰C)
100
90
80
70
60
50
40
30
0
200
400
600
800

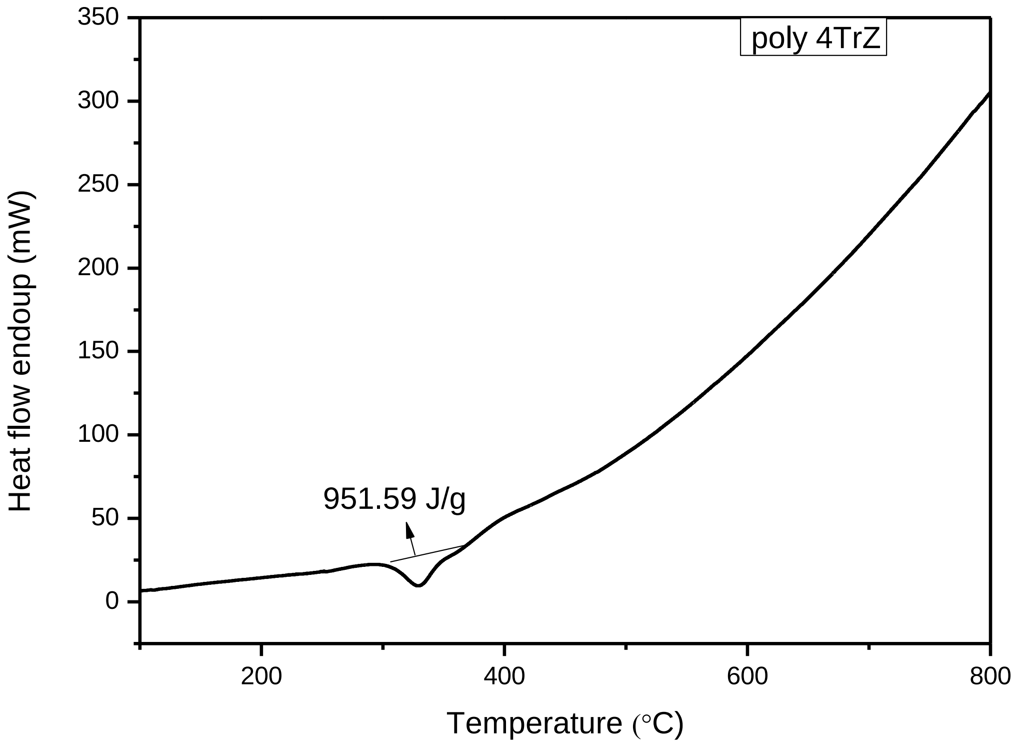

Figure S55& S56. Thermal studies of 3(g)

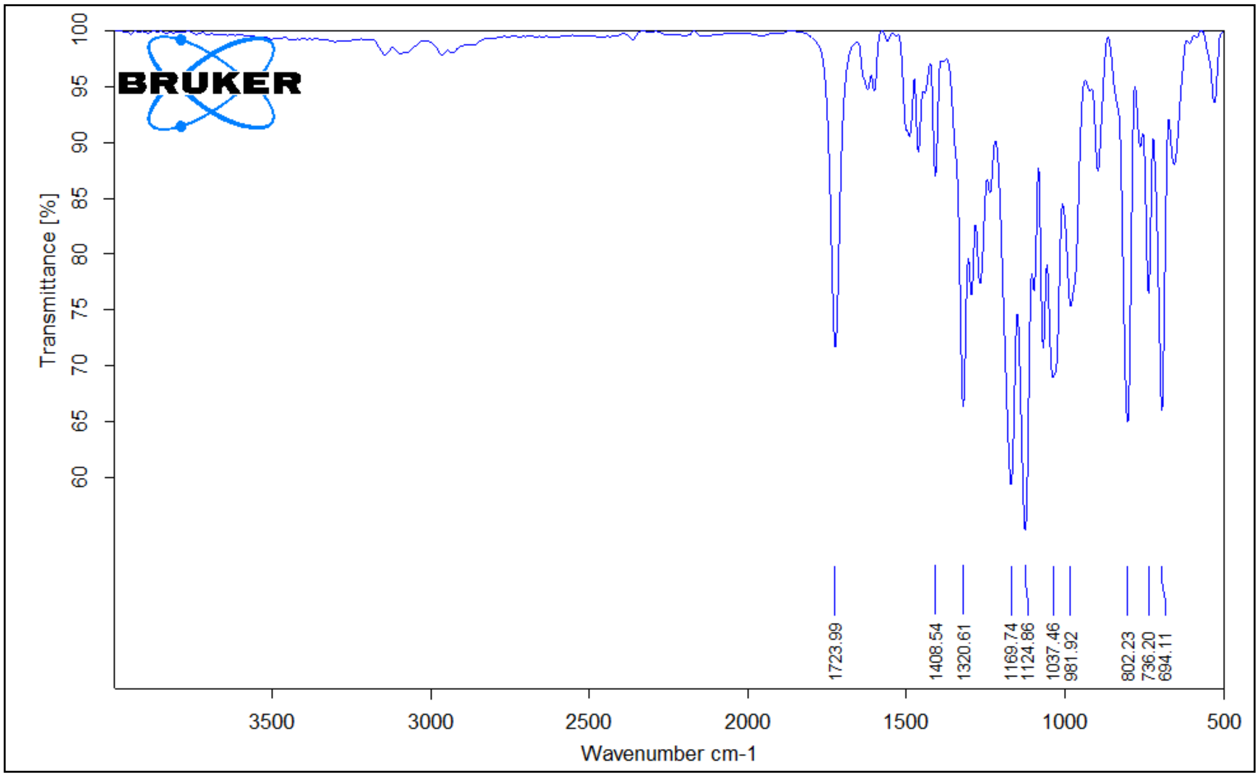

Figure S57. FTIR spectrum of of 2(c)

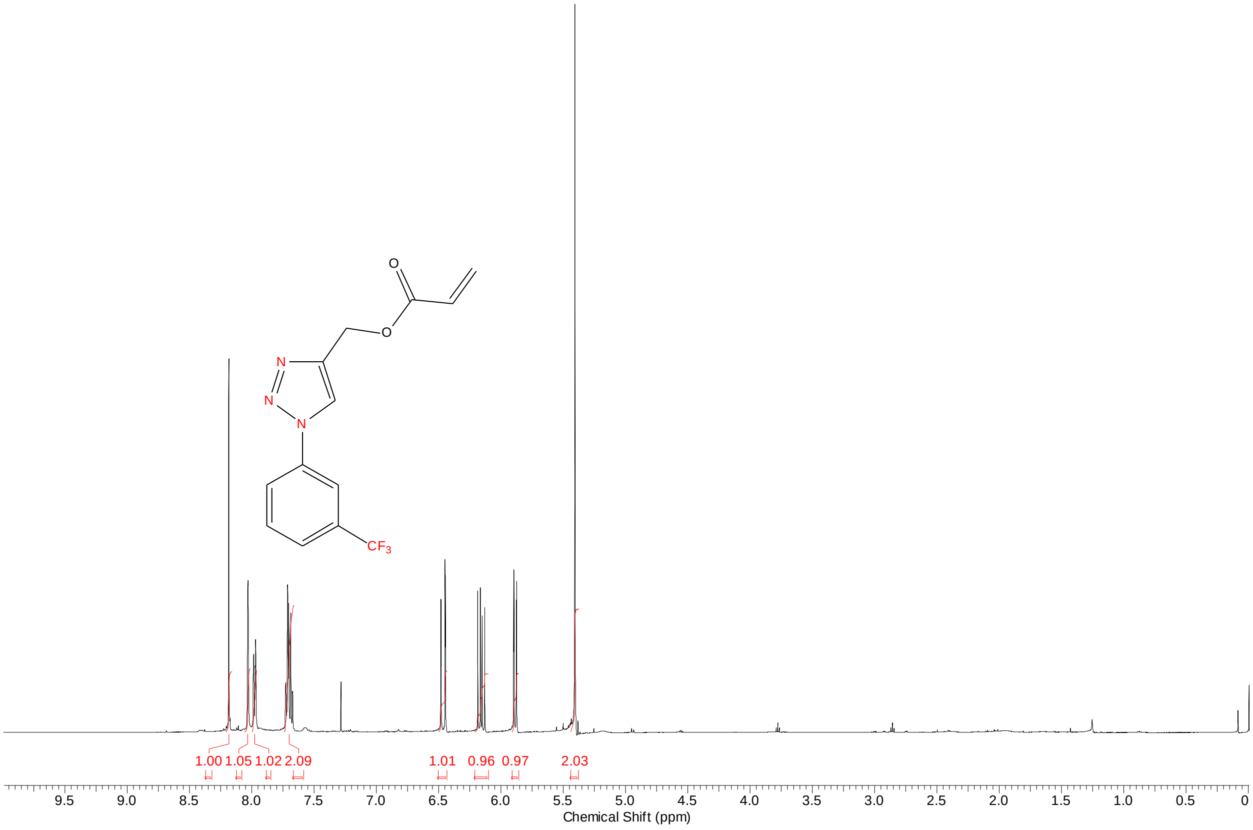

Figure S58. ^{1}H spectrum of of 2(c)

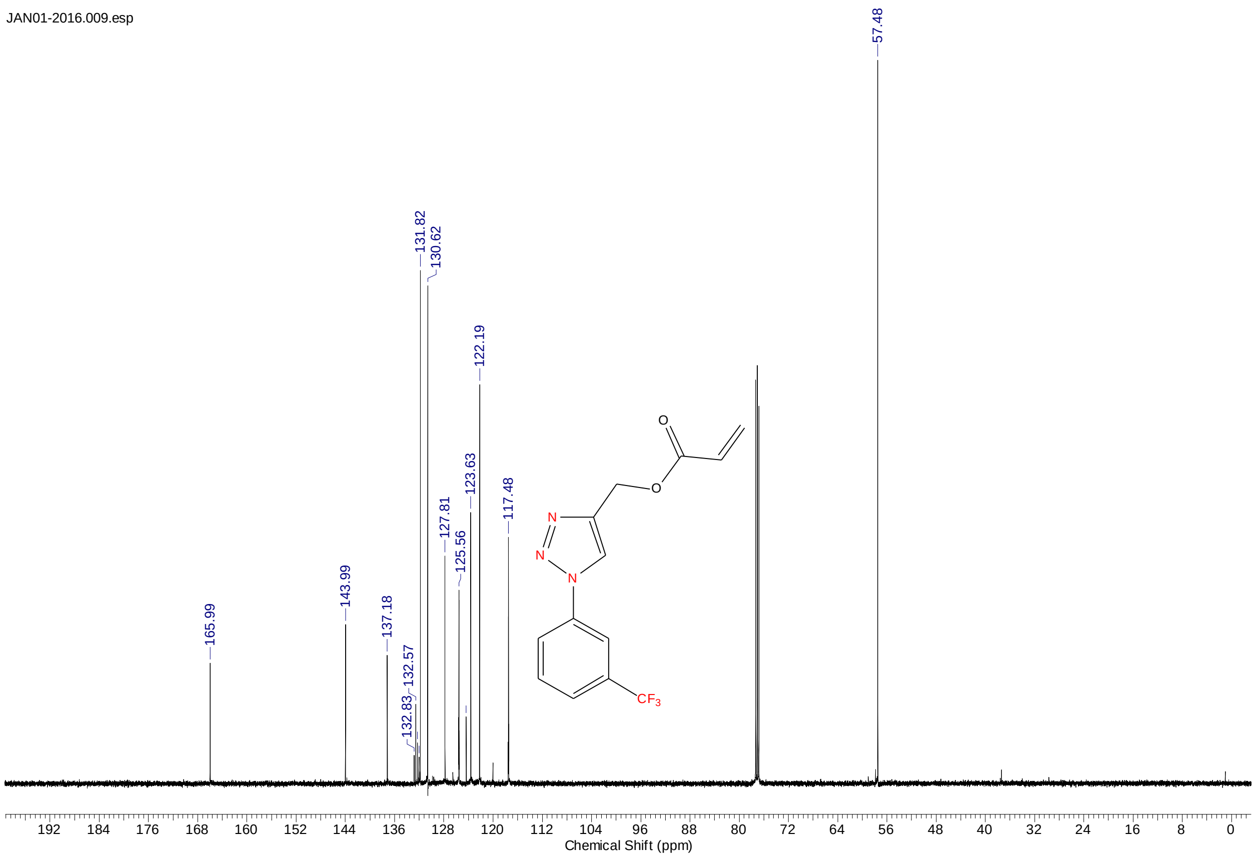

Figure S59. ^{13}C spectrum of of 2(c)

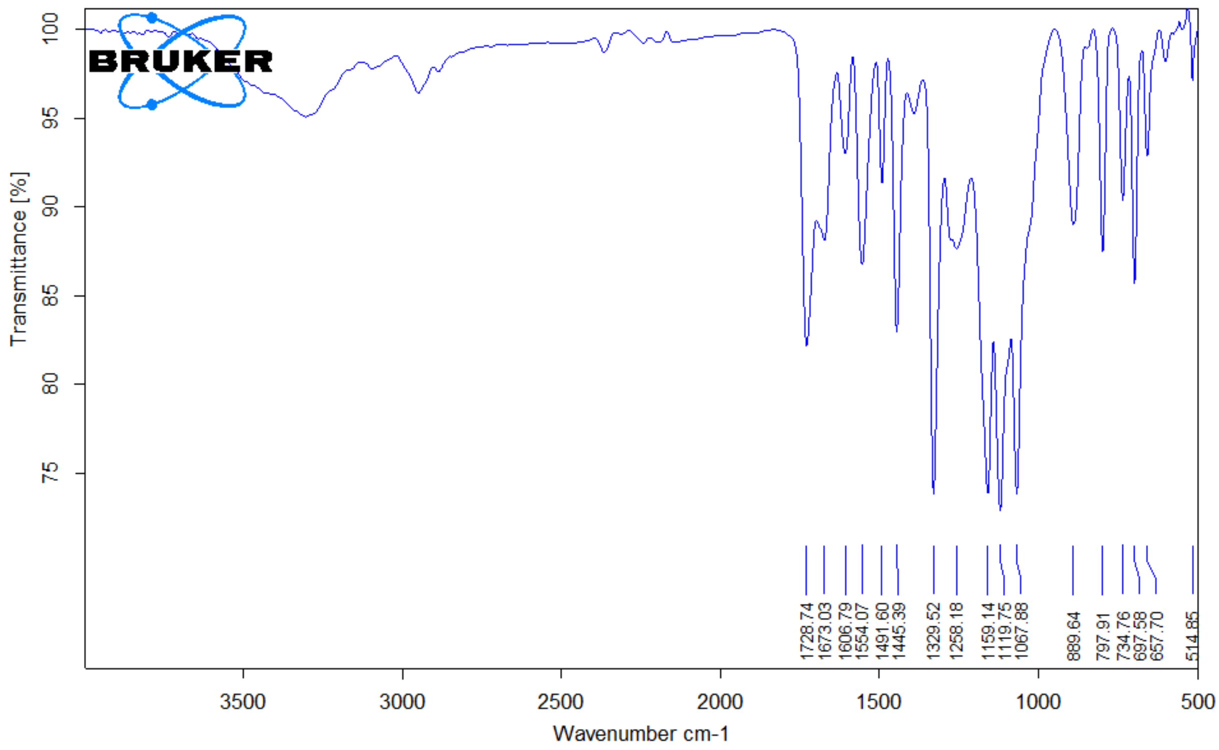

Figure S60. FTIR spectrum of of 3(h)

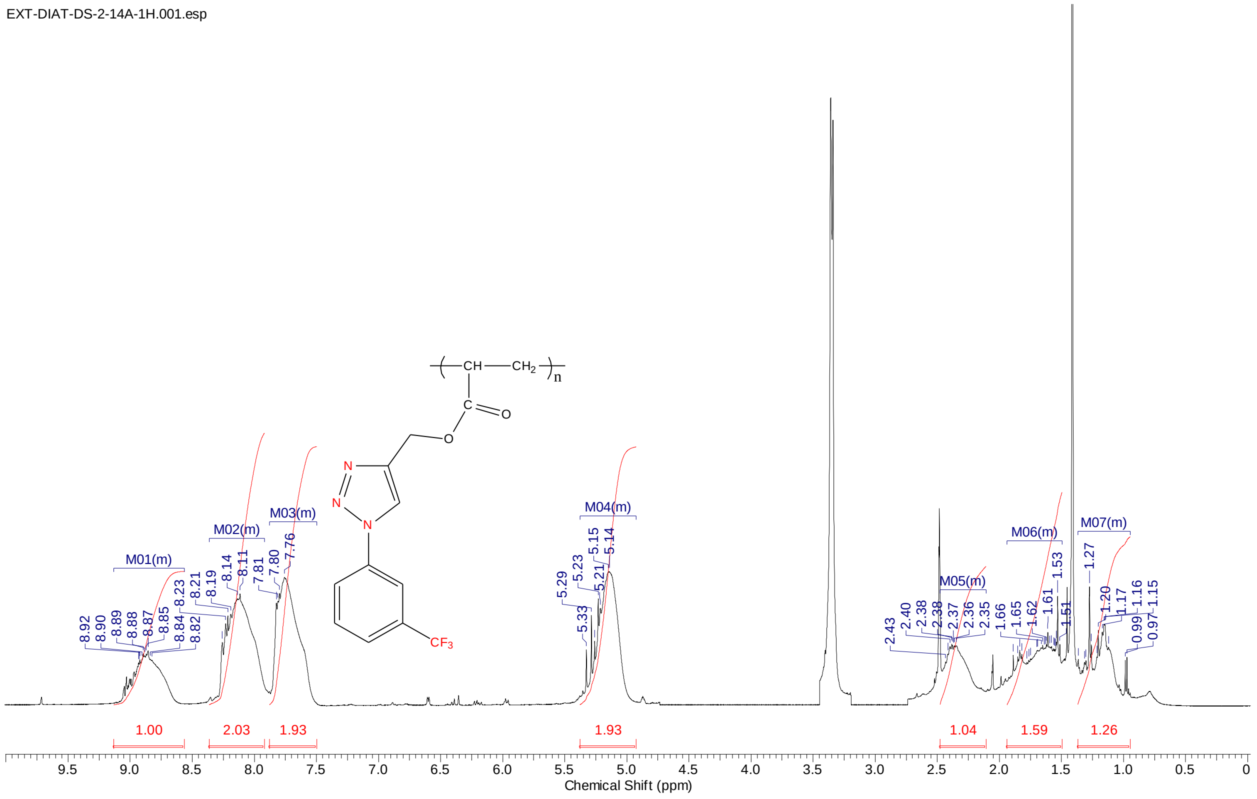

Figure S61. ^{1}H spectrum of of 3(h)

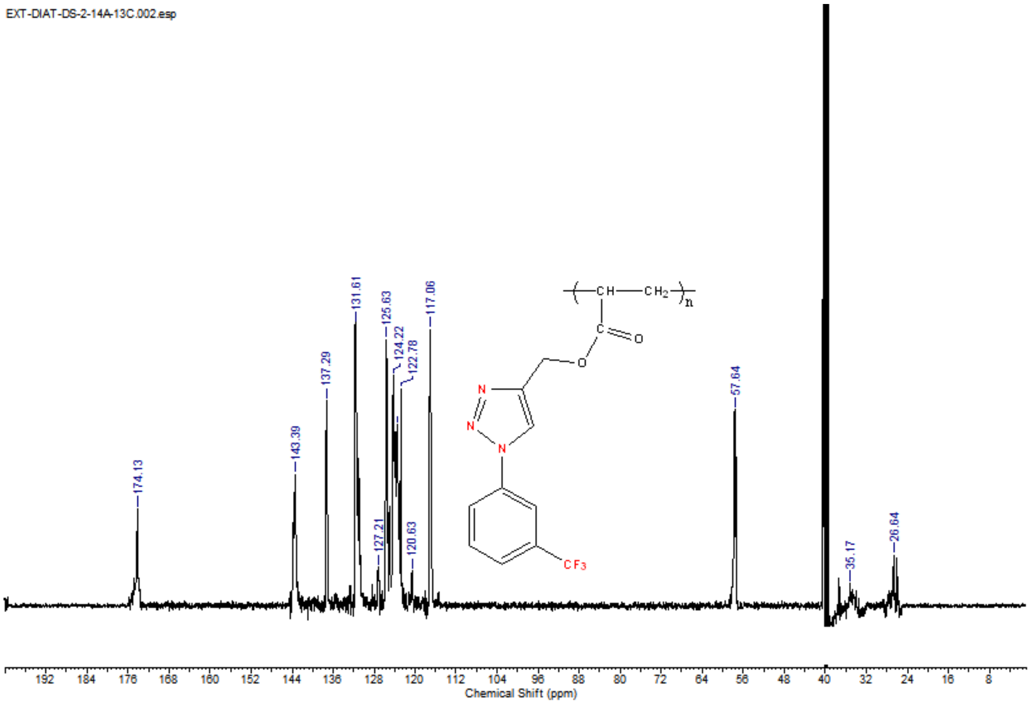

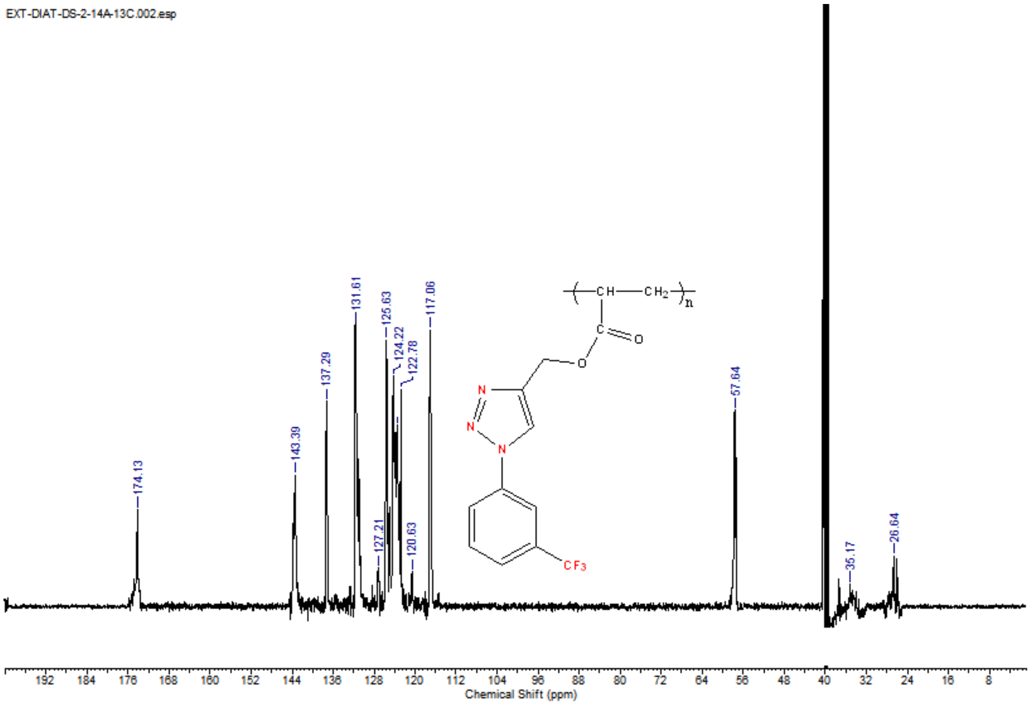

Figure S62. ^{13}C spectrum of of 3(h)

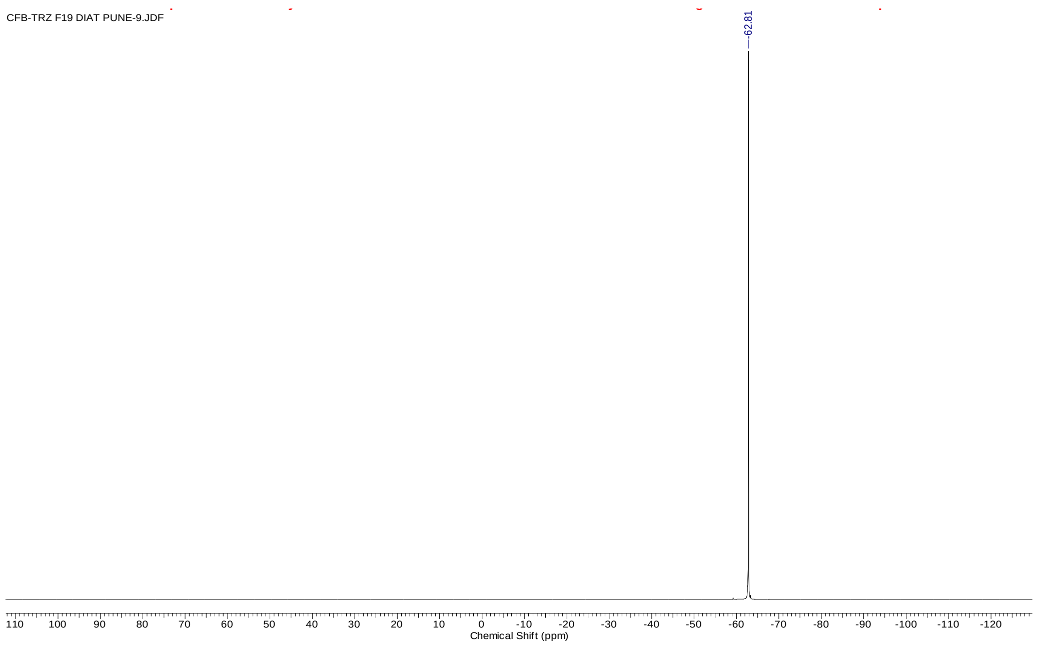

Figure S63. ^{19}F spectrum of 3(h)

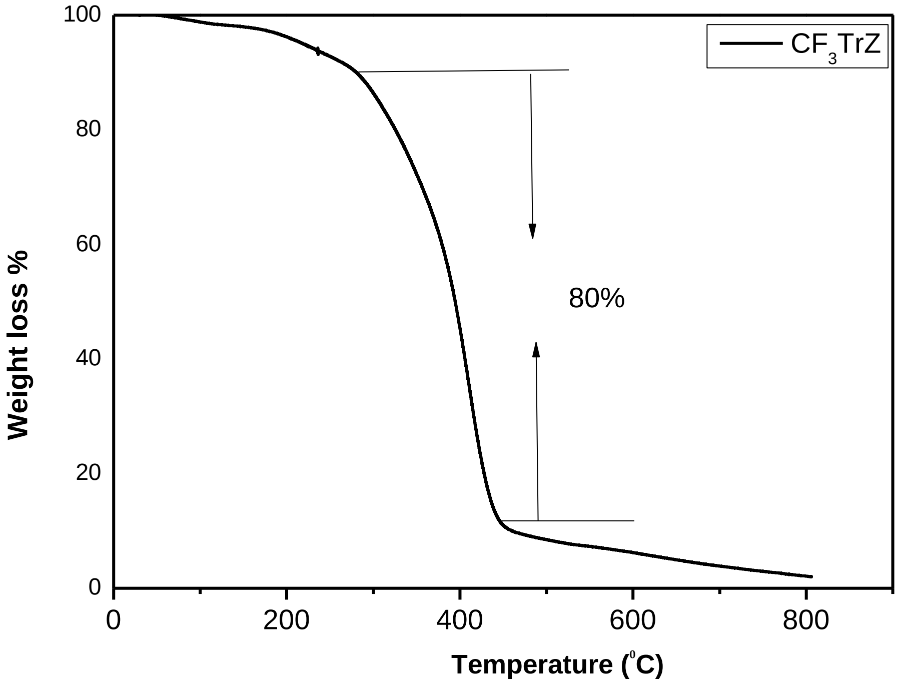

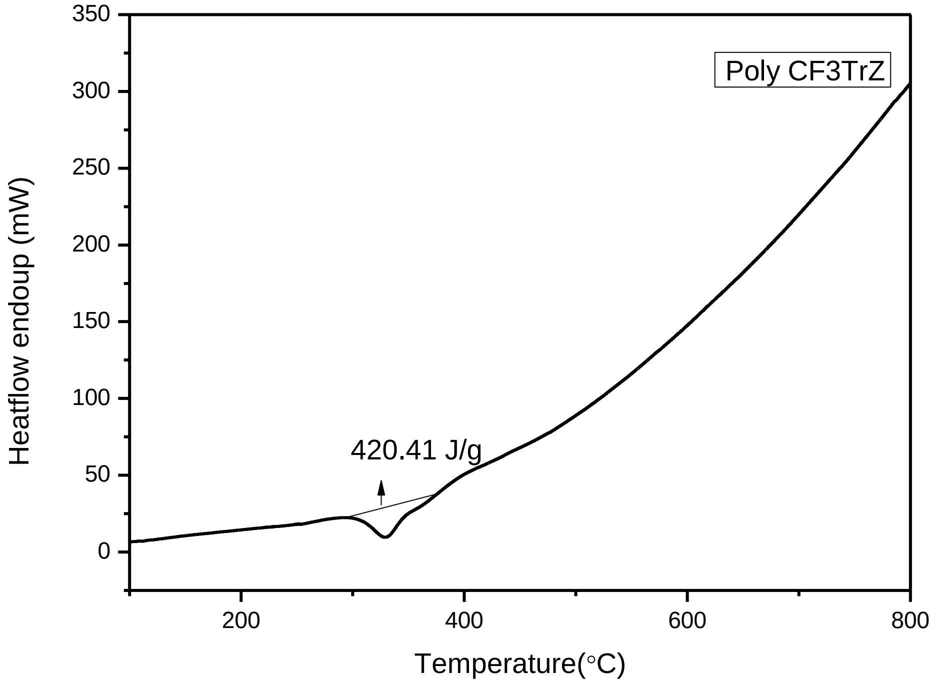

Figure S64 & S65. Thermal studies of 3(h)
